# Co-facial π–π Interaction Expedites Sensitizer-to-Catalyst Electron Transfer for High-Performance CO_2_ Photoreduction

**DOI:** 10.1021/jacsau.2c00073

**Published:** 2022-04-07

**Authors:** Jia-Wei Wang, Hai-Hua Huang, Ping Wang, Guangjun Yang, Stephan Kupfer, Yanjun Huang, Zizi Li, Zhuofeng Ke, Gangfeng Ouyang

**Affiliations:** † KLGHEI of Environment and Energy Chemistry, School of Chemistry, Sun Yat-sen University, Guangzhou 510275, China; ‡ School of Materials Science & Engineering, PCFM Lab, Sun Yat-sen University, Guangzhou 510275, China; § Institute of New Energy Materials and Low Carbon Technology, School of Material Science and Engineering, Tianjin University of Technology, Tianjin 300384, China; ∥ Friedrich Schiller University Jena, Institute of Physical Chemistry, Helmholtzweg 4, Jena 07743, Germany; ⊥ Instrumental Analysis and Research Center, Sun Yat-sen University, Guangzhou 510275, China; @ Chemistry College, Center of Advanced Analysis and Gene Sequencing, Zhengzhou University, Zhengzhou 450001, China; ¶ Guangdong Provincial Key Laboratory of Emergency Test for Dangerous Chemicals, Guangdong Institute of Analysis (China National Analytical Center Guangzhou), Guangzhou 510070, China

**Keywords:** π interaction, CO_2_ reduction, homogeneous catalysis, dynamic interaction, non-covalent interaction, electron transfer, dual emission

## Abstract

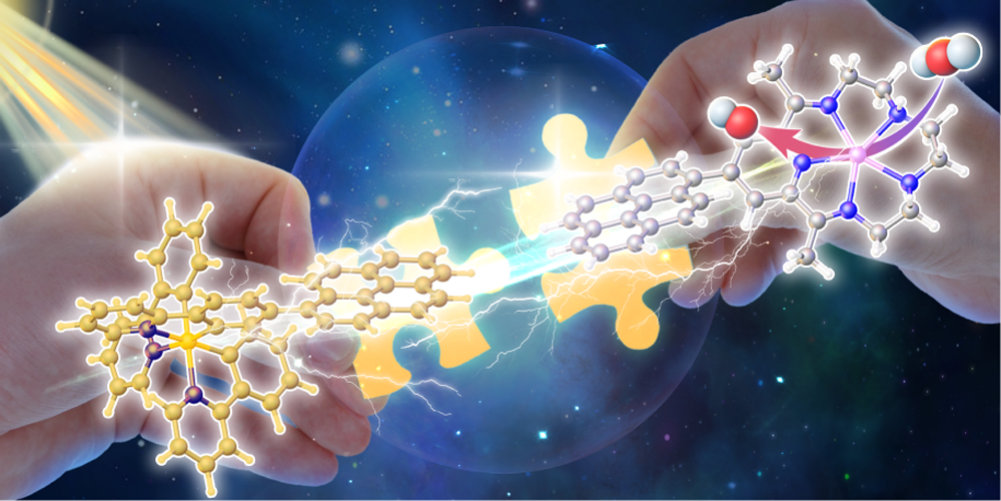

The sunlight-driven reduction of CO_2_ into carbonaceous fuels can lower the atmospheric CO_2_ concentration and provide renewable energy simultaneously, attracting scientists to design photocatalytic systems for facilitating this process. Significant progress has been made in designing high-performance photosensitizers and catalysts in this regard, and further improvement can be realized by installing additional interactions between the abovementioned two components, however, the design strategies and mechanistic investigations on such interactions remain challenging. Here, we present the construction of molecular models for intermolecular π–π interactions between the photosensitizer and the catalyst, via the introduction of pyrene groups into both molecular components. The presence, types, and strengths of diverse π–π interactions, as well as their roles in the photocatalytic mechanism, have been examined by ^1^H NMR titration, fluorescence quenching measurements, transient absorption spectroscopy, and quantum chemical simulations. We have also explored the rare dual emission behavior of the pyrene-appended iridium photosensitizer, of which the excited state can deliver the photo-excited electron to the pyrene-decorated cobalt catalyst at a fast rate of 2.60 × 10^6^ s^–1^ via co-facial π–π interaction, enabling a remarkable apparent quantum efficiency of 14.3 ± 0.8% at 425 nm and a high selectivity of 98% for the photocatalytic CO_2_-to-CO conversion. This research demonstrates non-covalent interaction construction as an effective strategy to achieve rapid CO_2_ photoreduction besides a conventional photosensitizer/catalyst design.

## Introduction

Visible-light-stimulated reduction of CO_2_ continues to attract attention as it may serve as a bifunctional pathway to convert sunlight into carbonaceous fuels and to impair the greenhouse effect concurrently.^1−3^ The process is nonetheless sluggish without a catalyst because of the inertness of the CO_2_ molecule and also suffers from the potentially low selectivity caused by competitive hydrogen evolution.^4^ In this context, diverse strategies to establish high-performance photocatalytic systems for CO_2_ reduction have been exploited. Major efforts have been devoted to designing potent catalysts and photosensitizers (PSs). Metal complexes as molecular catalysts are appealing for their high efficiency and selectivity, well-defined redox properties and accessible mechanistic studies for rational optimization.^5,6^ Some pioneering families of molecular catalysts for the photoreduction of CO_2_ include Ir-^7^/Re-^8^/Ru-^9^/Cu-^10^/Ni-^11^/Co-^1,2^/Fe-^12^/Mn-based^13^ polypyridine complexes, Co-^14,15^/Fe-based^16,17^ porphyrins, and so forth. In most cases, the molecular catalysts were applied in cooperation with metal complexes as PSs which often feature precious metals such as Re,^18^ Ru,^19,20^ or Ir,^17,21^ and so forth. Organic dyes^22^ or earth-abundant metal complexes^23^ have been documented as the more economical PSs. Recently, the use of semiconducting materials as PS alternatives have also received increasing attention, such as Cu_2_O,^24^ graphitic carbon nitride (*g*-C_3_N_4_),^25,26^ perovskites,^27^ and so forth.

**Scheme 1:**
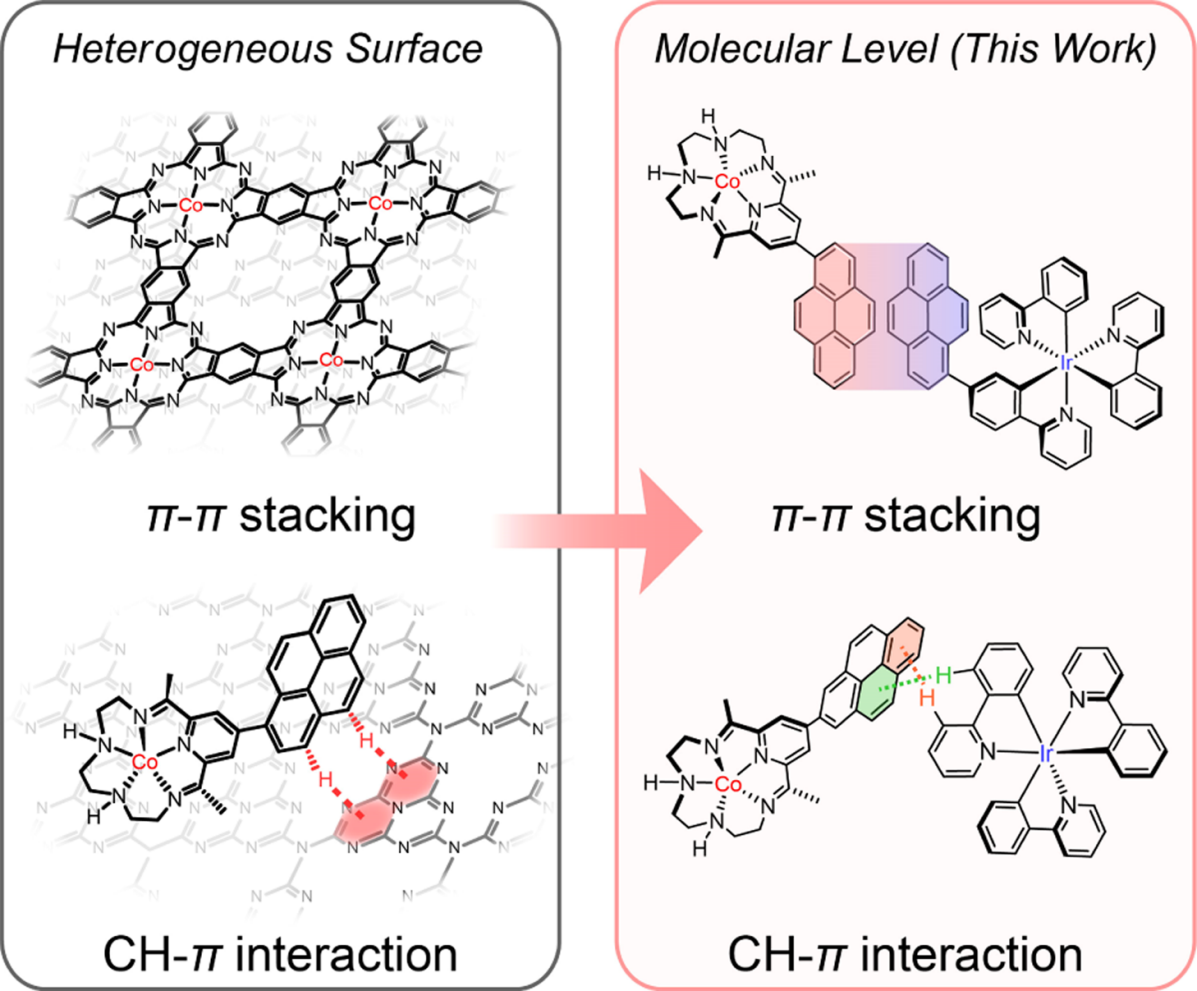
Demonstration of the Research Concept The molecular systems designed for the exploration of presumable π–π interactions which are reminiscent of molecule-heterogenized catalysts.

With the above significant progress in optimizing each component in a molecule-based photocatalytic system for CO_2_ reduction, further improvement of the photocatalytic efficiency desires new design strategies, as the rate-determining factor may no longer be the photophysical properties of PSs or the catalytic kinetics of catalysts. In this context, the strengthening of interactions between catalysts and PSs is promising to raise the performances to a higher level. It has been found that the addition of a redox mediator as electron relay can speed up electron delivery from the excited PS to the catalyst.^28,29^ Besides, the covalent or non-covalent attachment between the PS and the catalyst has been of intensified interest recently. For the examples of covalent connection, Ishitani et al. have connected Ru/Ir/Os PSs with Re/Mn catalysts via covalent bonds to achieve ultrafast intramolecular electron transfer and boost apparent quantum efficiency (Φ) for CO_2_ reduction.^30−32^ On the other hand, for the heterogeneous systems, the covalent linking between *g*-C_3_N_4_ and diverse molecular catalysts, including several metal porphyrins^25,33,34^ or metal quaterpyridine catalysts,^25,26^ has also achieved good activity in the photoreduction of CO_2_ to CO. However, the conventional covalent linkers, such as acetylene or amide bonds, are vulnerable and can be cleaved during photocatalysis, leading to catalytic performances below expectation.^35,36^ Also, the back electron transfer via the covalent linkers^37,38^ may take place to induce charge recombination and diminished activity. The additional synthetic steps of covalent linkers will also inevitably increase the efforts and expense.^21,39^ In contrast, the dynamic binding between the PS and the catalyst can be more advantageous to offer a self-adaptable binding for sustained photocatalysis.^40^ We have utilized reversible, dynamic coordinative interaction to facilitate the electron transfer between a pyridine-appended Ir PS and various molecular catalysts for CO_2_ photoreduction, reaching an impressive Φ of 27.9%.^21^ The non-covalent interactions have also been applied in molecule-immobilized heterogeneous catalysts,^41−43^ especially the utilization of π–π interactions between planar molecular catalysts and two-dimensional functional surfaces (Scheme 1).^44−48^ A pioneering instance^42^ for photocatalytic CO_2_ reduction is the π–π stacking of 2D cobalt polyphthalocyanine on mesoporous *g*-C_3_N_4_. We have also tried to establish CH−π interactions between *g*-C_3_N_4_ and a non-planar, pyrene-appended cobalt macrocycle, [Co^II^(PYN5) (CH_3_CN)_2_](ClO_4_)_2_, [**Co-PYN5**; PYN5 = (2*E*,12*E*)-2,13-dimethyl-14-(pyren-1-yl)-3,6,9,12-tetraaza-1(2,6)-pyridinacyclotridecaphane-2,12-diene].^43^ However, the characterization of such non-covalent binding between the catalyst and semiconductor at the molecular level remains elusive, mostly limited in the indirect experimental evidence and computational modeling rather than direct instrumental evidences. The main difficulties are that the molecule–material interface is complicated owing to the non-uniform material surface and that diverse interactions are involved in molecule immobilization besides π–π interaction, such as electrostatic and van der Waals forces. These problems lead to challenges in investigating the functions of interaction in catalytic performances and mechanisms and thus preclude further optimization.

**Figure 1:**
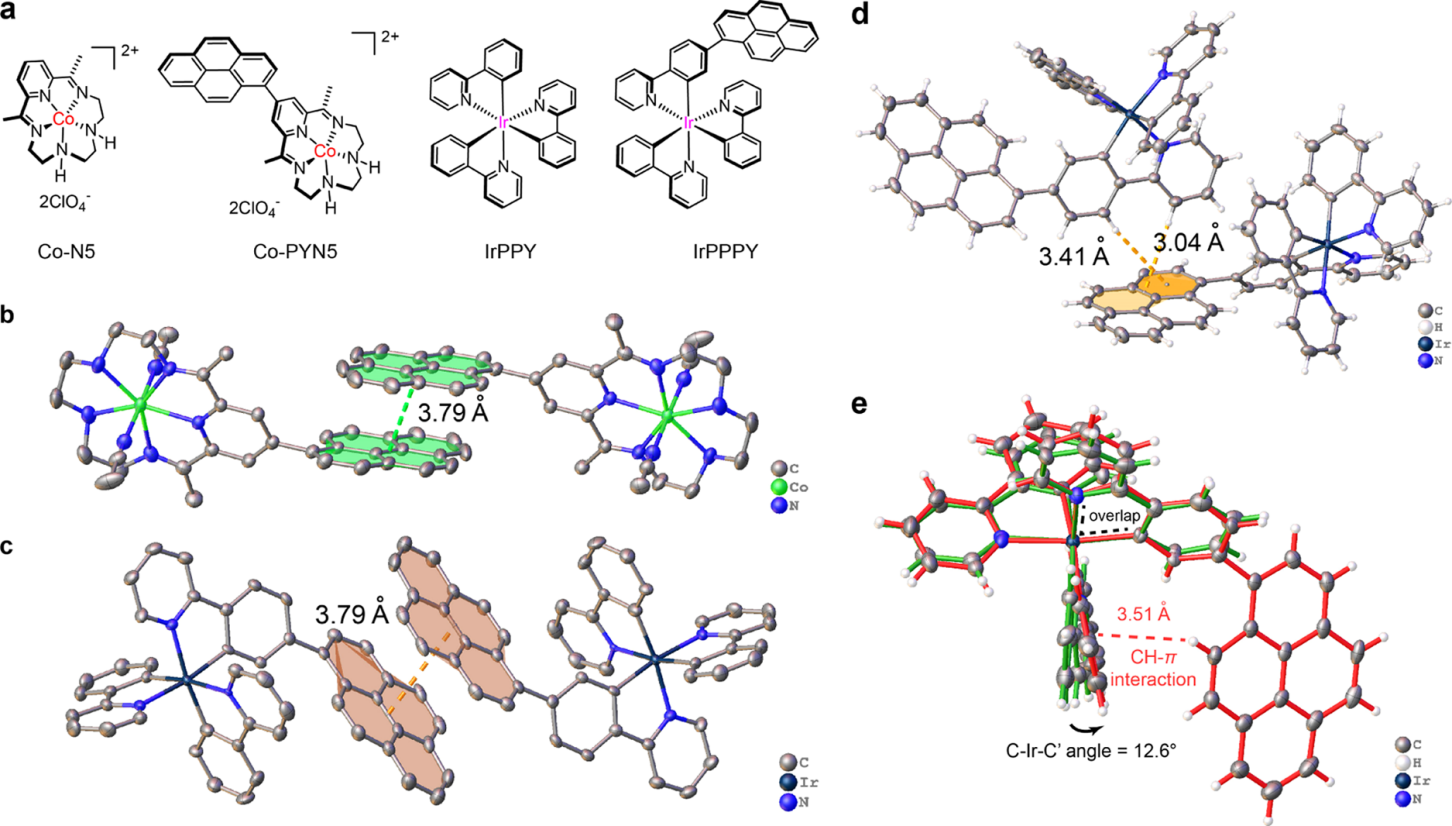
Structures. (a) Schematic structures of **Co–N5**, **Co-PYN5**, **IrPPY**, and **IrPPPY**. Co-facial π–π interactions in the crystal structures of (b) **Co-PYN5** and (c) **IrPPPY**. (d) Intermolecular CH−π interaction between **IrPPPY** molecules. (e) Structural variations between **IrPPY** (green-bonded) and **IrPPPY** (red-bonded), showing the CH−π interaction. The centroids of the whole/partial pyrenyl rings are employed for the definition of atom-to-plane or interplane distances in Olex2 program.^49^ The counteranions and solvent molecules are omitted for clarity. Probability is 50% for the crystal structures.

Against this backdrop, we anticipate the construction of fully molecular systems with deliberately designed non-covalent interactions between PSs and catalysts, which can build molecular-level models for mechanistic studies on the molecule-heterogenized systems and achieve high-performance photocatalytic CO_2_ reduction simultaneously. For the above purposes, as shown in Figure 1a, we selected *fac*-Ir(ppy)_3_ (**IrPPY**; ppy = 2-phenylpyridine) and [Co^II^(N5) (CH_3_CN)_2_](ClO_4_)_2_ (**Co–N5**; N5 = (2*E*,12*E*)-2,13-dimethyl-3,6,9,12-tetraaza-1(2,6)-pyridinacyclotridecaphane-2,12-diene) as the prototypes of the PS and the catalyst, respectively, which were decorated with pyrene modules, affording a new PS, *fac*-Ir(pppy) (ppy)_2_ (**IrPPPY**; pppy = 2-(4-(pyren-1-yl)phenyl)pyridine), and the **Co-PYN5** catalyst. We first investigated the structural, redox, and photophysical properties of these PSs and catalysts. Next, the pair-wise combinations of the two PSs and two catalysts revealed different π–π interaction modes, as investigated by ^1^H NMR titration and DFT calculations. The diverse binding modes have been found to be crucial to differentiate the photocatalytic performances for visible-light-driven CO_2_ reduction to CO, accomplishing an optimized Φ(CO) of 14.3 ± 0.8% with the coupling between **IrPPPY** and **Co-PYN5**. The combined in-situ spectroelectrochemical steady-state and time-resolved spectroscopic measurements have elucidated that the co-facial π–π interaction significantly accelerates the electron transfer from the excited-state **IrPPPY** to **Co-PYN5**. Ultimately, we have successfully demonstrated the characterization, function, and mechanism of π–π interaction in photocatalytic CO_2_ reduction by utilizing a purely molecular system, which, to our knowledge, has seldom been reported before.

## Results and Discussion

### Synthesis and Structures

**Co-PYN5** was synthesized by condensation with triethylenetetramine and the pyrene-substituted 2,6-diacetylpyridine, in which Co^2+^ serves as the metal center template.^43^*Caution!* Perchlorate salts of metal complexes with organic ligands are potentially explosive and should be handled in small quantities with care. The crystal structure of **Co-PYN5** is similar to that of **Co–N5**, with a seven-coordinated Co(II) center with two axial CH_3_CN ligands and the equatorial macrocyclic ligand, forming a distorted pentagonal bipyramid geometry. The aromatic hydrogens near the C–C bond between pyrene and pyridine cause steric repulsion to prevent the planar configuration of the **Co-PYN5** molecule, giving a dihedral angle of 61.6 ± 0.2° between the pyrenyl group and the main macrocycle. More notably, co-facial π–π stacking between of two pyrene rings from adjacent **Co-PYN5** cations can be observed with a pyrene–pyrene distance of 3.79 ± 0.03 Å (between the centroids, Figure 1b).

On the other hand, the heteroleptic **IrPPPY** was prepared by the reaction between [Ir(ppy)_2_]^+^ and the pppy ligand (see the Experimental Section for details) in ethanol. The facial feature of **IrPPPY** can be clearly identified from its crystal motif. Like **Co-PYN5**, a dihedral angle of 47.8 ± 0.3° can be found between the pyrenyl and phenyl groups in the pppy ligand due to the steric effect of those adjacent protons. Interestingly, more than one π interaction modes can be noted, including the co-facial π–π stacking between pyrene moieties, also a 3.79 ± 0.02 Å separation (Figure 1c), as well as CH−π interactions between phenyl protons from the pppy ligand and the pyrene ring from another **IrPPPY** molecule, with point-to-plane distances of 3.04 ± 0.04 and 3.41 ± 0.01 Å, respectively (Figure 1d). Moreover, an intramolecular CH−π interaction with 3.51 ± 0.02 Å can be observed within **IrPPPY**, between its pyrenyl H atom and the phenyl ring of an adjacent ppy ligand, leading to the more distorted octahedral geometry relative to **IrPPY** (Figure 1e). The above multiple π interactions in **IrPPPY** structure can be reasons for the easy crystallization of **IrPPPY** in a stationary CH_2_Cl_2_ or CH_3_CN solution.

### NMR Studies

NMR studies were carried out to investigate the structural information. We first measured the ^1^H NMR spectra of **Co–N5** and **Co-PYN5** (Figure S1). The paramagnetic nature of Co(II) in **Co–N5** leads to the vanished proton signals in 0 ∼ 15 ppm range, whereas the spectrum of **Co-PYN5** shows clear peaks from the nine protons in the pyrenyl group (Figure S2). In addition, the 10-times increment of the concentration of **Co-PYN5** induced a slight but visible shift in its ^1^H NMR spectrum, suggesting the interaction between each **Co-PYN5** molecule.^50^

On the other hand, the ^1^H NMR spectra of **IrPPY** and **IrPPPY** display highly overlapped proton signals. Thus, 2D correlation spectroscopy and nuclear Overhauser effect spectroscopy techniques were further carried out to identify the proton positions for subsequent studies, and the results are shown in Figures S3–S8. It can be noticed that the chemical shifts vary significantly with different concentrations of **IrPPPY** (Figure S9), consistent with the multiple π–π interactions within its structure.^50^

### Redox Properties

We then compared the redox properties of Co catalysts and Ir PSs by cyclic voltammetry in anhydrous CH_3_CN. All potentials were referenced against ferrocenium/ferrocene (Fc^+^/Fc) as an internal standard. Under a N_2_ atmosphere, the cyclic voltammograms (CVs; Figure S10) of the two Co complexes are similar, both revealing two irreversible reduction waves, assignable to Co^II/I^ reduction (at ca. −1.4 V) and ligand-centered reduction (at ca. −2.0 V), respectively.^5^ The first Co^II/I^ redox couples are irreversible due to the structural changes and axial ligand dissociations.^51^ Upon the introduction of CO_2_ atmosphere, catalytic currents were observed at the second reduction waves in the CVs of the two Co complexes. Further addition of proton source, 2,2′,2″-trifluoroethanol (TFE), which would be used for photocatalysis (vide infra), could significantly enhance the catalytic currents with initiation at ca. −1.7 V for both Co complexes. More notably, the catalytic current of **Co-PYN5** at the ligand reduction waves (at ca. −2.0 V) is higher than that of **Co–N5** under the same conditions. Overall, the above electrochemical results indicate the relatively high catalytic activity of **Co-PYN5**, which is attributable to the enhanced reducing force of the redox-active moiety by pyrene attachment.

For the Ir PSs, we recorded their CVs under N_2_ to obtain their one-electron oxidative/reduction potentials (Figures S11 and S12). The values are summarized in Tables 1 and S1, which are quite close between the two Ir PSs, indicating that the pyrene decoration on the **IrPPY** prototype negligibly changes the redox properties.

**Table 1:** Selected Redox Properties and Photophysical Properties of Ir PSs^a^

complex	*E*_red_ (V)	*E*_ox_ (V)	λ_em_ (nm)	ε_450_ (M^–1^ cm^–1^)	Φ_em_ (%)	τ_0_ (μs)	*E*_q,red_ (V)^b^	*E*_q,ox_ (V)^b^	Δ*E*_s_^c^(cm^–1^)
**IrPPY**	–2.42	0.31	529	5000	54.3	1.39	0.10	–2.21	534
**IrPPPY**	–2.41	0.33	525 (λ_em,1_)	3300	2.5	0.82 (λ_em,1_)	0.17	–2.25	447 (λ_em,1_)
			636 (λ_em,2_)			27.3 (λ_em,2_)			150 (λ_em,2_)
			690 (λ_em,3_)			26.7 (λ_em,3_)			58 (λ_em,3_)

a The values were measured in deaerated, dry CH_3_CN at 298 K unless otherwise noted. *E*_red_ and *E*_ox_ are the first reduction and oxidation wave potentials, respectively, which were determined by CVs (Figures S11 and S12).

b *E*_q,red_ and *E*_q,ox_ are the reductive and oxidative quenching potentials of the excited-state PSs, respectively, which were determined according to ref (58).

c The thermally induced Stokes shift (Δ*E*_s_) values were measured in deaerated, dry DCM.^59^

### Photophysical Properties

**Figure 2:**
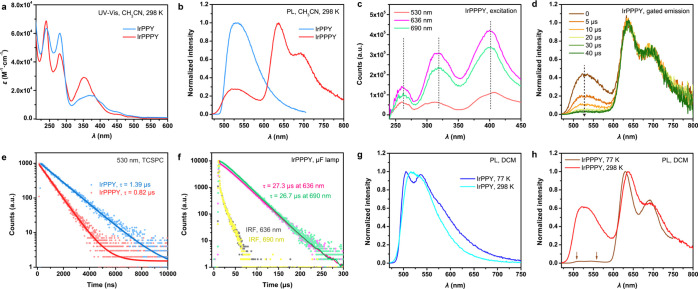
Photophysical properties of Ir PSs. (a) UV–vis and (b) steady-state fluorescence spectra of **IrPPY** (blue) and **IrPPPY** (red) in CH_3_CN. (c) Excitation spectra for the emission peaks of **IrPPPY** at 530 (red), 636 (pink), and 690 nm (green). (d) Gated emission with increasing gating with 5 μs increment for 50 μM CH_3_CN solution of **IrPPPY**. Lifetime measurements of 50 μM CH_3_CN solution of **IrPPY** (blue) and **IrPPPY** (red) for (e) ^3^MLCT emission, as well as (f) **IrPPPY** for ^3^IL emission (pink for 636 nm, green for 690 nm). The instrument response functions (yellow and gray) as background signals are shown. (g) Normalized emission spectrum of 50 μM DCM solution of **IrPPY** at 77 K (navy) or 298 K (cyan). (h) Normalized emission spectrum of 50 μM DCM solution of **IrPPPY** at 77 K (crimson) or 298 K (red).

**Figure 3:**
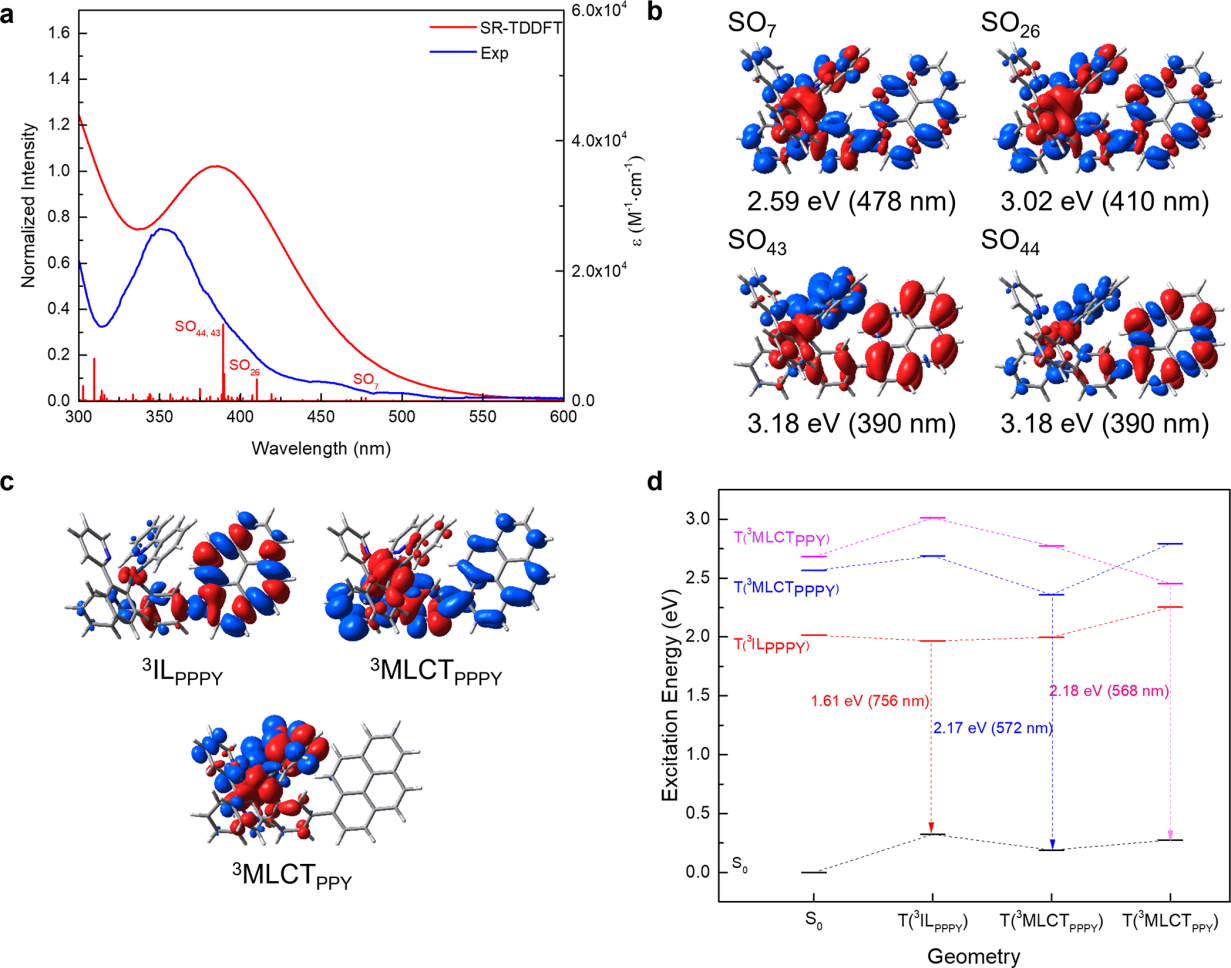
Simulated photophysics of IrPPPY. (a) Experimental absorption spectrum (blue) and simulated (red) absorption of **IrPPPY** obtained by means of SR-TDDFT. (b) Prominent electronic transitions contributing to the electronic absorption are visualized by charge density differences; charge transfer takes place from red to blue. (c) Emissive triplet states, ^3^IL_PPPY_, ^3^MLCT_PPPY_, and ^3^MLCT_PPY_ and respective charge density differences within their fully optimized equilibrium structures. (d) Predicted energy diagram highlighting the three radiative decay pathways, that is, via ^3^IL_PPPY_, ^3^MLCT_PPPY_, and ^3^MLCT_PPY_ emissions.

UV–vis absorption and fluorescence spectroscopies were carried out to verify the photophysical properties. The results of UV–vis spectra between two Co complexes indicate that both of their absorbance cease at wavelengths below 470 nm (Figure S13). The comparison also shows that the absorption of **Co-PYN5** is much stronger, in which the pyrene module may enhance the Co(II) *d*–*d* transition absorbance.^52^ On the other hand, a main difference of the UV–vis spectra of both Ir complexes (Figure 2a) is the enhanced absorbance at ca. 350 nm in that of **IrPPPY**, contributed by the extended conjugation which promotes the spin-allowed singlet metal-to-ligand charge transfer (^1^MLCT) involved in this MLCT/ILCT transition.^53^ Further insights into the nature of the electronic transitions underlying the absorption features of **IrPPPY** were obtained using the (scalar relativistic) time-dependent density functional theory (SR-TDDFT)—employing the scalar relativistic zeroth-order regular approximation (SR-ZORA; Figures 3a and S14).^54^ These simulations reveal mainly three excitations, that is, the spin–orbit (SO) states, SO_26_, SO_43_, and SO_44_ centered at 410 and 390 nm (3.02 and 3.18 eV), respectively, contributing to the main absorption feature centered at approximately 350 nm (Figure 3b). It is noteworthy that the weakly absorbing states, SO_26_ (mixed MLCT_PPPY_/ILCT_PPPY_) and SO_43_ (mixed MLCT_PPY_/LLCT_PPY_), feature significant triplet character, whereas the strongly dipole-allowed excitation into SO_44_ is mainly of singlet intra-ligand (IL_PPPY_) nature. Furthermore, and typically for structurally related Ir complexes, the visible-light absorption at around 450 nm shows a weakly absorbing spin-forbidden ^1/3^MLCT transition,^55^ which can be assigned according to the simulations to an excitation into SO_7_ (at 478 nm, 2.59 eV)—a mixed MLCT transition to both ppy/pppy ligand spheres. The underlying spin-free states, for example, S_1_, T_5_, and T_6_, feature pronounced spin–orbit coupling (SOC) of up to nearly 1000 cm^–1^ (Tables S2–S7), indicating that the triplet states in **IrPPPY** are rapidly accessible upon ultrafast intersystem crossing.^56^

Next, steady-state fluorescence experiments for the emission of Ir PSs were implemented at room temperature in Ar-saturated anhydrous CH_3_CN (Figure 2b). We noticed that the presence of ambient or even traces of air can efficiently quench the fluorescence intensity of both Ir complexes, demanding the degassing of measured solutions. The steady-state fluorescence spectra first provide the calculation basis of their photo-redox potentials with the assistance of the above electrochemical and UV–vis spectroscopic data. As summarized in Table 1, the photo-redox potentials of the two Ir PSs do not vary much, with the variations within 0.05 V, showing their similar redox abilities for photocatalysis. For the structures of their fluorescence spectra, **IrPPY** shows a characteristic ^3^MLCT emission at 529 nm and that of **IrPPPY** also displays a qualitatively similar ^3^MLCT emission at 525 nm, albeit with a much lower fluorescence intensity. Interestingly, additional emission peaks were found at 636 and 690 nm. These observations suggest that the population of the ^1^MLCT excited state of **IrPPPY** is transferred to and thus shared between ^3^MLCT emission and population of the long-wavelength excited states. We also observed that the additional emission of **IrPPPY** was diminished by air more significantly than its ^3^MLCT emission (Figure S15a). The above characteristic fluorescent structure and the high oxygen sensitivity infer that the additional emission peaks most possibly come from the triplet intra-ligand excited state (^3^IL),^57^ which can be supported by the following control experiments and quantum chemical calculations.

To further verify the presence of dual emission, the excitation spectra corresponding to the three emission peaks were first collected (Figure 2c). The excitation spectra for the emissions at 636 and 690 nm are almost superimposable, indicating that the two emission peaks originate from the same excited state. In contrast, the peaks in the excitation spectrum for emission at 525 nm are notably shifted in contrast to the above, which manifests that the excited state for 636 and 690 nm emissions differs from ^3^MLCT, proving the dual emission from **IrPPPY**.

We then detected the different excited states of **IrPPPY** under the gated excitation of a microsecond flash lamp.^59^ As shown in Figure 2d, the ^3^MLCT emission at 525 nm generally faded with the lamp pulse increasing from 0 to 40 μs, whereas that of ^3^IL emission was retained even at the 40 μs gating. The observation further indicates the presence of dual emission and the long-lived ^3^IL-based phosphorescence.

The outcome of gated emission inspired us to estimate the lifetimes of dual emission by the combined measurements with laser-based time-correlated single-photon counting method and the flash lamp, in which the former was used for ^3^MLCT lifetimes, generally shorter than 10 μs and the latter was for phosphorescence lifetimes longer than 1 μs. On the one hand, as determined by the time-correlated single-photon counting technique, the ^3^MLCT lifetime of **IrPPPY** is relatively short-lived in contrast to that of **IrPPY** (0.82 vs 1.39 μs; Figure 2e), further demonstrating the less populated ^3^MLCT emission. On the other hand, the phosphorescence lifetime at 636 nm was evaluated as 27.3 μs, more long-lived than the ^3^MLCT ones, which is consistent with the abovementioned gated emission results and thus confirms the dual emission (Figure 2f). It can also be perceived that the lifetime of the shoulder near 690 nm (26.7 μs) is close to the one at 636 nm, further supporting that this red shoulder belongs to the same emissive state as the one at 636 nm.

To further prove the assignment of the long-lived excited state for the emission at 636 and 690 nm as ^3^IL, we also carried out the fluorescence measurements under 77 K in CH_2_Cl_2_ solutions, in which CH_2_Cl_2_ was chosen for low-temperature fluorescence measurements.^53^ The steady-state fluorescence spectra at 77 K were recorded and the detailed data are shown in Tables 1 and S1. The ^3^MLCT emission peaks in both the steady-state spectra of Ir PSs display a doubly split structure for different triplet substrates (Figure 2g,h).^53^ In contrast, the other emission of **IrPPPY** was only slightly narrowed and shifted. Further quantitative comparison can be achieved by estimating the thermally induced Stokes shifts (Δ*E*_s_; Table 1).^59^ The Δ*E*_s_ value of **IrPPY** is close to that of the first emission of **IrPPPY** (534 vs 447 cm^–1^), which are both significantly larger than the value for the latter emission peaks of **IrPPPY**. The larger Δ*E*_s_ of the emission at ca. 520 nm indicates its more polar feature, a characteristic of ^3^MLCT emission.^53^ In contrast, the much smaller Δ*E*_s_ from the emission at over 600 nm reveals a non-polar feature, most presumably the ^3^IL emission.^59^

Finally, the fully relaxed equilibrium structures of the lowest three triplet states within the Franck–Condon point were obtained using pysisyphus^60^—our recently introduced external optimizer that is aware of excited states. Within these structures, the emission wavelengths were obtained at the TDDFT level of theory. Thereby, the ^3^MLCT_PPY_ and ^3^MLCT_PPPY_ excited-state emissions are predicted at 568 and 572 nm, respectively, as well as the ^3^IL_PPPY_ emission at 756 nm (Figure 3c,d), in agreement with the abovementioned experimental assignments. Further details and results with respect to the excited-state properties are collected in the Supporting Information.

Additionally, we observed that **IrPPY** is strongly emissive in the degassed CH_3_CN with an emission quantum yield (Φ_em_) of 54.3% at 450 nm excitation, whereas Φ_em_ of **IrPPPY** is significantly lower (2.5%). Many reported pyrene-appended PSs show very low Φ_em_ values because their ^3^IL or ^3^ILCT states are not highly emissive.^55^ It is interesting to note that the fluorescence intensity increases linearly with respect to the concentration of **IrPPPY** (Figure S15b,c) and that the MLCT excited-state lifetime remains almost identical at different concentrations of **IrPPPY** (Figure S15d). These observations demonstrate that the multiple π interactions between each **IrPPPY** molecule (Figure 1) do not lead to a significant aggregation-caused quenching effect^61^ to diminish its luminescence performances.

Overall, the abovementioned spectroscopic measurements elucidate the photophysical properties of the two Ir PSs and especially the unexpected dual emission behavior of **IrPPPY**. To our knowledge, the Ir-based PSs exhibiting dual emission behaviors are rare,^58,62^ especially for those pyrene-modified ones,^52,55,63^ and only a similar case was documented for the Ru-based PSs.^59^

### π–π Interaction Modes

With a clear understanding of the respective properties of Ir PSs and Co catalysts, we used the NMR titration method to characterize the π–π interaction between Ir and Co complexes (see the Experimental Section for details). At the beginning, the titration of **Co–N5** into the CDCl_3_ solution of **IrPPY** did not induce observable changes (Figure S16), indicating that no interaction exists between them and that the increasing Co(II) complex in the mixed solution will not alter the chemical shifts by its paramagnetic nature. In notable contrast, all the results of titration experiments for **IrPPPY**/**Co–N5** (Figure S17), **IrPPY**/**Co-PYN5** (Figure S18), and **IrPPPY**/**Co-PYN5** (Figure S19) showed shifted proton signals. It was unexpected to observe interactions between **IrPPPY**/**Co–N5** and **IrPPY**/**Co-PYN5** because one of their components has no pyrene moiety. With previous identifications on the proton positions, we recorded the shifted proton signals possible for interaction and subjected them to the calculation of binding constants (see the Experimental Section and Tables S8–S13 for detailed information). It can be seen that **IrPPPY**/**Co–N5** and **IrPPPY**/**Co-PYN5** both follow the 1:1 binding model, and the 1:1 binding constant (*K*_11_) of the former is much smaller than the latter (60 ± 2 vs 199 ± 7 M^–1^). Meanwhile, **IrPPY**/**Co-PYN5** was found to follow a non-cooperative 2:1 binding mode with a *K*_11_ of 115 ± 1 M^–1^. The magnitudes of these binding constants are reasonable as a reported H-bond-interacted photocatalytic system^8^ displays a non-covalent interaction binding constant of 300 M^–1^, which suggests that all these interactions should belong to the weak non-covalent interaction forces. The comparatively high *K*_11_ value of **IrPPPY**/**Co-PYN5** suggests the relatively strong interaction between the two components via the intermolecular, co-facial pyrene pair.

**Figure 4:**
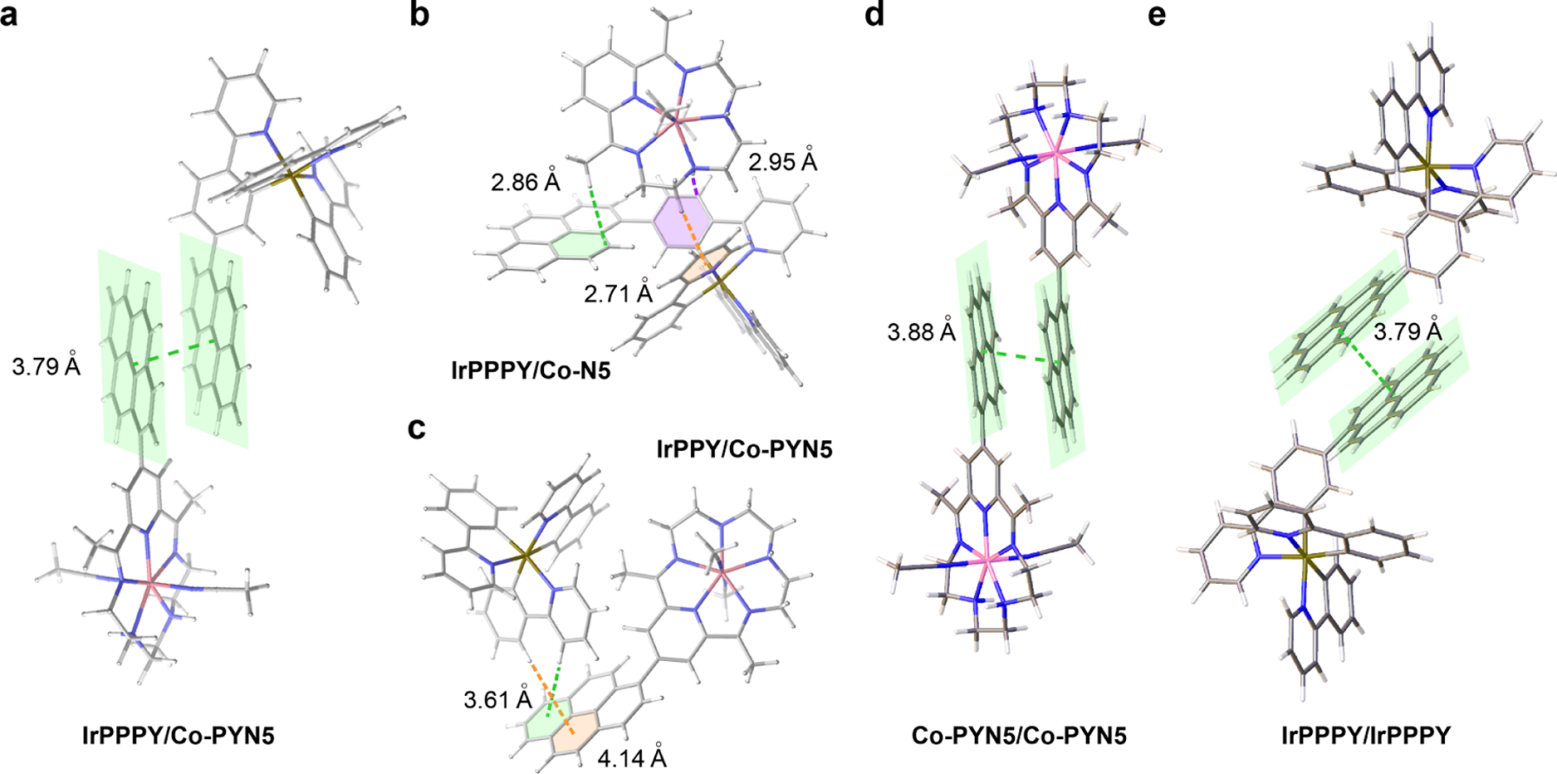
π interactions. DFT-simulated structures of (a) **IrPPPY**/**Co-PYN5**, (b) **IrPPPY**/**Co–N5**, (c) **IrPPY**/**Co-PYN5**, (d) **Co-PYN5**/**Co-PYN5** and (e) **IrPPPY**/**IrPPPY**. The interplanar or point-to-plane distances of the π interactions are noted. Counteranions are omitted for simplicity. Atom color: Ir, brown; Co, pink; C, gray; and H, white.

With the NMR titration results and preliminary conclusions, we further utilized DFT calculation to elucidate the possible π–π interaction modes between the Ir and the Co complexes and to shed light on their binding structures (Figure 4). First, a pyrene–pyrene distance of 3.79 Å is observed from the optimized structure of the **IrPPPY**/**Co-PYN5** couple, showing the co-facial π–π interaction. In the **IrPPPY**/**Co–N5** couple, besides two CH-π interactions between the alkyl protons in the N5 ligand and the pyrene/pyridyl rings of **IrPPPY** with the point-to-plane distances of 2.86 and 2.71 Å, respectively. There is also a NH−π interaction between the amine proton of **Co–N5** and the phenyl ring in the ppy moiety, with a point-to-plane distance of 2.95 Å. For the **IrPPY**/**Co-PYN5** couple, CH−π interactions exist between the phenyl/pyridinyl protons in **IrPPY** and the pyrene ring in **Co-PYN5** with the point-to-plane distances of 3.61 and 4.14 Å, respectively. The differences between the CH−π distances in the calculated structures (**IrPPPY**/**Co–N5** and **IrPPY**/**Co-PYN5** couple) and the crystal structures (**IrPPPY** and **Co-PYN5**) are probably due to the domination of the π–π stacking rather than the CH−π/NH−π interactions. Although the consideration of computational errors cannot be ruled out, the results of the calculated binding free energies (Table S14) suggest that the π–π interaction for the **IrPPPY**/**Co-PYN5** couple (−3.0 kcal mol^–1^) is stronger than the CH−π/NH−π interactions for either the **IrPPPY**/**Co–N5** (−2.2 kcal mol^–1^) or the **IrPPY**/**Co-PYN5** systems (−0.9 kcal mol^–1^). It should be noted that the binding free energy for the **IrPPPY**/**Co-PYN5** couple is in good agreement with the experimental *K*_11_ value of 199 M^–1^ (corresponding to a binding free energy of −3.1 kcal mol^–1^). The diverse π interactions for these couples have also been presented by reduced density gradient isosurface (Figures S20–S22).^64^ The good consistency between the abovementioned experimental and computational results indicates the well establishment of diverse π–π interaction modes.

On the other hand, we also noticed that self-interaction takes place for the pyrene-appended complexes on the basis of their crystal structures (Figure 1) as well as ^1^H NMR spectra at different concentrations (Figures S1 and S9). Further investigations on this self-interaction issue were enabled by additional DFT simulations (Table S14). The results of the calculated binding free energies (Table S14) suggest that the π–π interaction for the **IrPPPY**/**Co-PYN5** couple (−3.0 kcal mol^–1^) is close to that for the **IrPPPY**/**IrPPPY** pair (−3.3 kcal mol^–1^), whereas much stronger than the **Co-PYN5**/**Co-PYN5** system (−0.3 kcal mol^–1^). The trends in binding free energies are also consistent with the varied pyrene–pyrene distances of 3.79, 3.79, and 3.88 Å for the **IrPPPY**/**Co-PYN5**, **IrPPPY**/**IrPPPY**, and **Co-PYN5**/**Co-PYN5** couples, respectively (Figures 4d,f, S23, and S24). The cationic nature of **Co-PYN5** should inhibit their self-interaction trends from the electrostatic point of view. Despite the similar thermodynamic tendency of **IrPPPY** between the self-interaction and the combination with **Co-PYN5** (−3.3 vs −3.0 kcal mol^–1^), the overall equilibrium in the **IrPPPY**/**Co-PYN5** system (*n*_PS_/*n*_catalyst_ = 1:1 as the photocatalytic conditions) will favor the formation of the PS/catalyst couple rather than the self-stacked species due to the less favored stacking of **Co-PYN5** (−2.4 kcal mol^–1^, eq 1). This computational comparison indicates that the π–π interaction between **IrPPPY** and **Co-PYN5** should circumvent their own self-interactions, thus exhibiting a promotive effect in electron-transfer processes for photocatalysis.
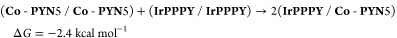
1

### Photocatalytic CO_2_ Reduction

**Figure 5:**
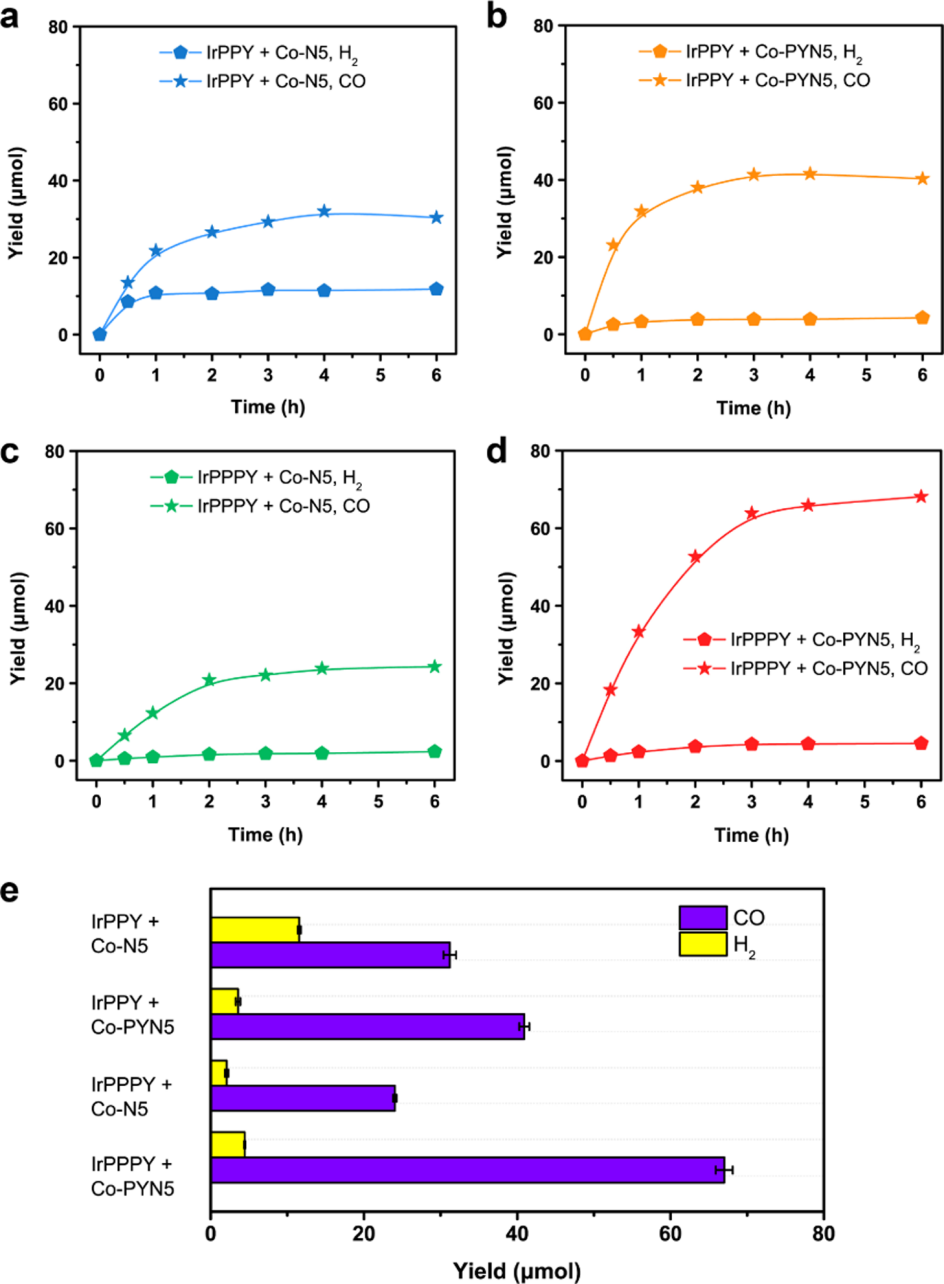
Photocatalytic CO_2_ reduction. (a–d) Time profiles and e yield comparison of photocatalytic CO (star) and H_2_ (pentagon) formation from a mixture of Co catalysts (0.1 mM), Ir PSs (0.1 mM), TEA (2.5 v %), TFE (4.0 v %), BIH (25 mM) in 4.0 mL CH_3_CN. (a) **IrPPY**/**Co–N5**; (b) **IrPPY**/**Co-PYN5**; (c) **IrPPPY**/**Co–N5**; and (d) **IrPPPY**/**Co-PYN5**.

Visible-light-driven CO_2_ reduction with the Co catalysts and Ir PSs was carried out to evaluate the influence of π–π interaction in photocatalysis. 0.1 mM of the catalyst and PS were used, respectively. The optimized conditions include 4 v % TFE as the proton source, 25 mM 1,3-dimethyl-2-phenyl-2,3-dihydro-1H-benzo[*d*]imidazole (BIH) as the sacrificial electron donor, and 2.5 v % triethylamine (TEA) as the deprotonation agent for BIH, CH_3_CN as the solvent, and blue LED (405, 425, or 450 nm) as the monochromic light source. Initially, all the pair-wise combinations of the two Co complexes and two Ir PSs can continuously produce considerable amounts of CO and trace H_2_ under 6 h of 450 nm light irradiation (Figure 5). Most markedly, the **IrPPPY**/**Co-PYN5** system displayed the best performance, with a CO yield of 68.1 ± 1.1 μmol and 94% selectivity within 6 h, consistent with a TON of 170.3 ± 7.3 and suggesting the key contribution of π–π interaction between pyrene moieties (Table 2, entry 4). The maximum apparent quantum efficiency, Φ(CO), was determined as 14.3 ± 0.8% at 425 nm (Table S15), with a high selectivity of 98%, which is comparable to many state-of-the-art molecular systems with precious-metal PSs and earth-abundant metal catalysts, such as Ru(bpy)_3_^2+^/Fe(qpy)^2+^ [qpy, 2,2′:6′,2″:6″,2‴-quaterpyridine; Φ(CO) = 8.8% at 450 nm],^65^ Ru(bpy)_3_^2+^/Ni(beptpy_2_)^2+^ [Φ(CO) = 11.1% at 450 nm],^11^ and Ru(phen)_3_^2+^/[CoZn(OH)L]^3+^ [L = N[(CH_2_)_2_NHCH_2_(*m*-C_6_H_4_)CH_2_NH(CH_2_)_2_]_3_N; Φ(CO) = 4.9% at 450 nm].^19^ The relatively high Φ(CO) at 425 nm can be ascribed to the higher Φ_em_ of **IrPPPY** at this wavelength (Table S16). The main obstacle to a higher stability and TON should be the decomposition of catalysts, as the re-addition of catalyst in the deactivated reaction mixture could reinitiate the CO production (Figure S25). This in turn manifests the good stability of the electroneutral Ir PSs used in this case. A more robust catalyst prototype to append a conjugated pendant can be anticipated for more sustainable catalysis, which is a warranted work in the future.

**Table 2:** Photocatalytic CO_2_ Reduction to CO with Different Combinations of PSs and Catalysts^a^

entry	PS	catalyst	*n*(CO) (μmol)	*n*(H_2_) (μmol)	TON(CO)^b^	CO %
1	**IrPPY**	**Co–N5**	30.4 ± 1.6	11.80 ± 0.22	76.0 ± 4.0	72
2	**IrPPY**	**Co-PYN5**	40.3 ± 1.7	4.27 ± 0.15	100.8 ± 4.3	90
3	**IrPPPY**	**Co–N5**	24.3 ± 1.1	2.34 ± 0.27	60.8 ± 2.8	91
4	**IrPPPY**	**Co-PYN5**	68.1 ± 1.1	4.52 ± 0.44	170.3 ± 7.3	94

a Standard condition: Ir PS (0.1 mM), Co catalyst (0.1 mM), TFE (4.0 v %), TEA (2.5 v %), and BIH (25 mM) in 4.0 mL CH_3_CN within 6 h of 450 nm LED irradiation (100 mW cm^–2^) under 1 atm CO_2_.

b TON = *n*_CO_/*n*_catalyst_, in which *n*_catalyst_ = 0.1 mM × 4.0 mL = 0.4 μmol.

Further comparison in Figure 5 and Table 2 displays that the CO production catalyzed by **Co-PYN5** was higher than the one by **Co–N5** with the use of **IrPPY** (TON 100.8 ± 4.3 vs 76.0 ± 4.0), consistent with the electrochemical results (Figure S10). In the meantime, the TON(CO) with **IrPPPY** was relatively low compared to **IrPPY** when **Co–N5** was employed (60.8 ± 2.8 vs 76.0 ± 4.0), suggesting a weaker driving force of **IrPPPY** for the photocatalytic reduction of CO_2_, which can be attributed to the less populated ^3^MLCT emission of **IrPPPY**. Albeit with this disadvantage, in sharp contrast, the CO yield of **IrPPPY**/**Co-PYN5** is nearly 1.7 times of that of the **IrPPY**/**Co-PYN5** system (TON 170.3 ± 7.3 vs 100.8 ± 4.3) and over 2 times of that of the prototypical **IrPPY**/**Co–N5** system (TON 170.3 ± 7.3 vs 76.0 ± 4.0). This contradiction further demonstrates the promotional effect of co-facial π–π interaction in catalysis. Moreover, it can be noticed that the CO selectivity was significantly promoted with the presence of increasing pyrenyl groups in either PS or catalyst structures (72%, 0 pyrenyl; 90%/91%, 1 pyrenyl; 94%, 2 pyrenyl). This tendency suggests that the pyrenyl groups may improve the selectivity toward CO_2_ reduction over H_2_ evolution with their hydrophobicity.

With the **IrPPPY**/**Co-PYN5** system as the example, all the components are necessary, as the absence of the catalyst, PS, CO_2_, light, and BIH could not generate significant amounts of CO (Table S17). The results of the isotope labeling experiment with ^13^CO_2_ source show the bare evolution of ^13^CO, further indicating that the produced CO should derive from CO_2_ rather than other organic components in the photocatalytic system (Figure S26). The addition of proton sources, such as phenol, water, and TFE can readily improve the catalytic performances (Figure S27 and Table S18). Moreover, the increasing concentration of the proton source, exemplified by TFE, can further enhance the CO yield (Figure S28 and Table S18).

### Photo-Induced Electron Transfer Pathway

**Figure 6:**
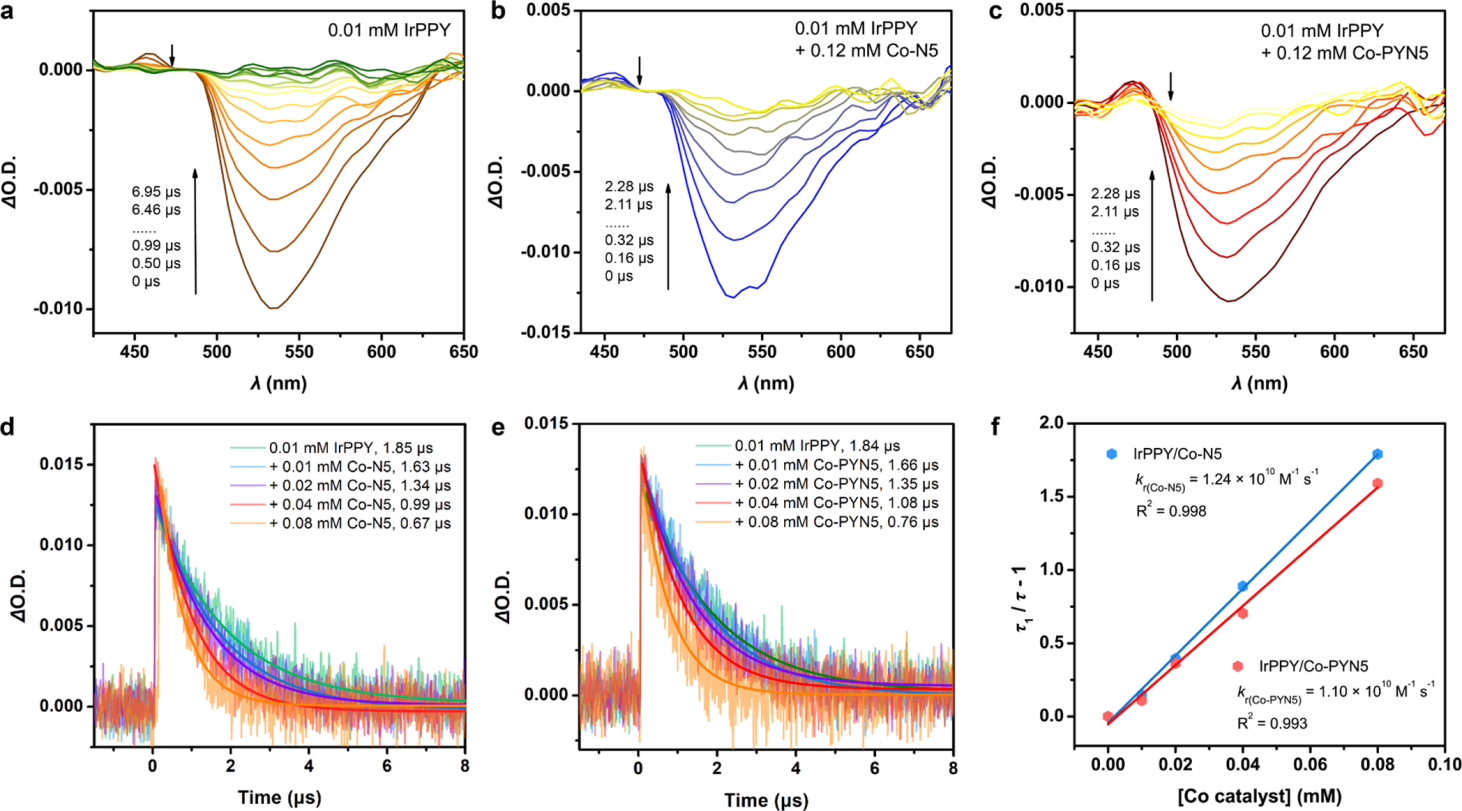
Nanosecond TA spectroscopy with IrPPY. TA spectra of (a) 0.1 mM **IrPPY**, (b) 0.1 mM **IrPPY** with 0.12 mM **Co–N5,** and (c) 0.1 mM **IrPPY** with 0.12 mM **Co-PYN5**. (d) Kinetic traces of **IrPPY** with 0 ∼ 0.08 mM **Co–N5** followed at 520 nm. (e) Kinetic traces of **IrPPY** with 0 ∼ 0.08 mM **Co-PYN5** followed at 520 nm. (f) Plots of (τ_1_/τ – 1) vs the concentration of **Co–N5** (blue) or **Co-PYN5** (red) with linear fitting. The data were collected in Ar-saturated CH_3_CN upon excitation at 450 nm.

To verify the photo-induced electron transfer pathway, we implemented a series of transient absorption (TA) spectroscopic measurements, concomitant with the in-situ spectroelectrochemistry to detect the spectral changes of PSs upon oxidation/reduction. Under the excitation at 450 nm, the TA spectrum of IrPPY* revealed a broad, strong bleaching at 500–650 nm (Figure 6a) with a lifetime of 1.85 μs, consistent with the ^3^MLCT emission structure of **IrPPY**. We then added BIH at increasing concentrations into the solution of **IrPPY**, in which the corresponding TA spectrum did not display a significant change at the ^3^MLCT emission, but slightly decreased lifetimes could be observed along with the increasing BIH at high concentrations (Figure S29). Meanwhile, we noticed that the addition of 2.5 v % TEA and further co-addition of BIH did not alter the TA spectrum and lifetime markedly as well. The reduced state of **IrPPY**, formed by electro-reduction, shows a UV–vis spectrum (Figure S30) which is different from the TA spectra upon the addition of BIH or BIH/TEA, which suggests the negligible formation of reduced **IrPPY** under the abovementioned TA conditions. The abovementioned observations indicate the unfavorable reductive quenching of **IrPPY** with BIH or BIH/TEA (eqs 2–4), in which the latter sped up the reduction by deprotonation of BIH (eq 5) and thus gave a steeper slope.^66^ Eventually, the decreasing lifetimes afford relatively small reaction constants, *k*_r_, via eq 6, at 10^7^ M^–1^ s^–1^ magnitude.^67^
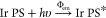
2
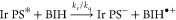
3
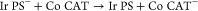
4
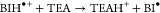
5
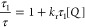
6
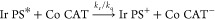
7
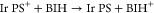
8
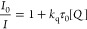
9

On the other hand, the addition of **Co–N5** (Figure 6b) or **Co-PYN5** (Figure 6c) also induced negligible variations on the TA spectra, respectively. However, the lifetimes were significantly shortened, giving much higher reaction constants for both catalysts at 10^10^ M^–1^ s^–1^ magnitude (Figure 6d,e), suggesting the more feasible oxidative quenching pathway in eqs 2, 7, and 8. The *k*_r_ values are similar for the two Co complexes (1.24 × 10^10^ M^–1^ s^–1^ for **Co–N5** and 1.10 × 10^10^ M^–1^ s^–1^ for **Co-PYN5**), showing their similar quenching abilities (Figure 6f). The absence of TA spectral variation may be attributed to the insignificant spectral changes of the oxidized **IrPPY** in the range of 400–700 nm (Figure S31) or the fast charge recombination between the oxidized **IrPPY** and the reduced Co complexes under non-catalytic conditions. However, despite the unchanged TA temporal evolution, the notably decreased lifetimes can support the significant oxidative quenching. Consequently, the abovementioned TA results on **IrPPY** indicate that the oxidative quenching pathway takes the majority.

**Figure 7:**
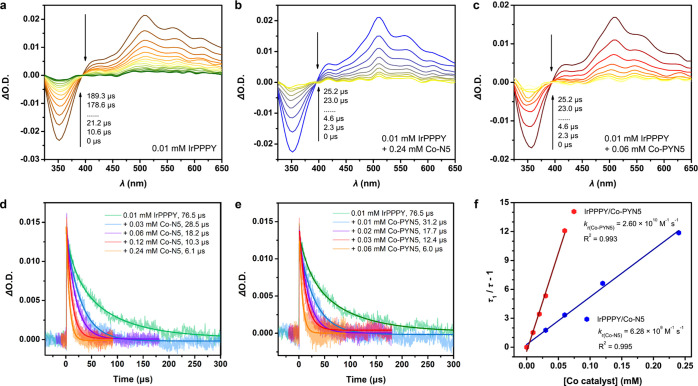
Nanosecond TA spectroscopy with IrPPPY. TA spectra of (a) 0.1 mM **IrPPPY**, (b) 0.1 mM **IrPPPY** with 0.24 mM **Co–N5**, and (c) 0.1 mM **IrPPPY** with 0.06 mM **Co-PYN5**. (d) Kinetic traces of **IrPPPY** with 0 ∼ 0.24 mM **Co–N5** followed at 520 nm. (e) Kinetic traces of **IrPPPY** with 0 ∼ 0.06 mM **Co-PYN5** followed at 520 nm. (f) Plots of (τ_1_/τ – 1) vs the concentration of **Co–N5** (blue) or **Co-PYN5** (red) with linear fitting. The data were collected in Ar-saturated CH_3_CN upon excitation at 450 nm.

Next, the TA spectroscopy was also performed on **IrPPPY** under parallel conditions. Initially, the TA spectrum of **IrPPPY** excited at 450 nm is markedly different from that of **IrPPY**. The ^3^MLCT emission, to a less extent, was overlapped by a positive strong absorption band from just above 400 nm and tailing at over 650 nm, corresponding to the ^3^IL absorption (Figure 7a). In addition, a negative band with absorption at around 350 nm can be assigned to the ground-state bleaching of **IrPPPY**. A much longer lifetime of 76.5 μs was detected due to the merged ^3^MLCT/^3^IL nature of the excited **IrPPPY** (IrPPPY*). Like **IrPPY**, small *k*_r_ values (<10^8^ M^–1^ s^–1^) were estimated with the addition of BIH or TEA/BIH, indicative of the lethargic reductive quenching (Figures S32 and S33). In contrast, evidently decreased lifetimes were detected upon the addition of both Co catalysts (Figure 7d,e). Also, the catalyst-added TA spectra were negligibly changed (Figure 7b,c), also possibly due to the minute spectral changes at >300 nm for the oxidized **IrPPPY** (Figure S34) and fast charge recombination in a non-catalytic setup. More importantly, the *k*_r_ of **Co-PYN5** is nearly four times of that of **Co–N5** (26.0 vs 6.28 × 10^9^ M^–1^ s^–1^, Figure 7f), showing a much faster electron delivery from IrPPPY* to **Co-PYN5**, apparently thanks to the co-facial π–π interaction. This advantage should enable the highest catalytic activity of **IrPPPY**/**Co-PYN5** system in light-stimulated CO_2_ reduction. The abovementioned results also manifest that the photocatalytic system with **IrPPPY** mainly follows the oxidative quenching pathway.

**Figure 8:**
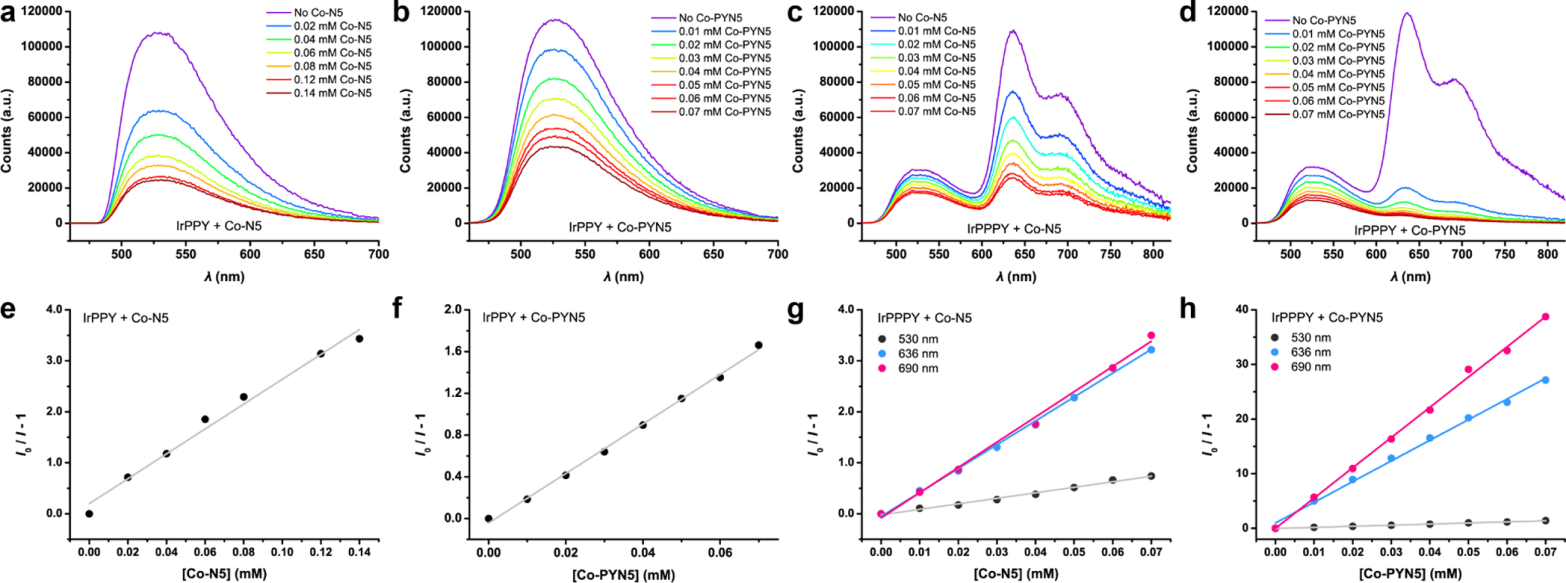
Steady-state fluorescence quenching. (a–d) Fluorescence spectra of a CH_3_CN solution containing 0.05 mM Ir PSs in the absence and presence of varying concentrations of Co catalysts, respectively. (e–h) Linear fittings of the ratio of fluorescence intensities of Ir PSs vs [Co catalysts]. (a,e) **IrPPY**/**Co–N5**; (b,f) **IrPPY**/**Co-PYN5**; (c,g) **IrPPPY**/**Co–N5**; and (d,h) **IrPPPY**/**Co-PYN5**.

With the TA results, we further evaluated the phosphorescence quenching ability of BIH, **Co–N5,** or **Co-PYN5** with steady-state fluorescence measurements, respectively. Here, the unexpected dual emission behavior of **IrPPPY** enables the separation of the quenching behaviors toward different triplet excited states (see below) and quantitatively estimate the apparent quenching constants, *k*_q_, by the Stern–Volmer plots. The results are also summarized in Table 3, which mainly indicate that the quenching of Ir PSs by Co complexes (Figure 8) are much notable than that by BIH (Figure S35) within similar concentration ranges, suggesting that the electron transfer from the photo-excited Ir PS to the Co catalyst is more feasible. Moreover, the possible absorption of emission of Ir PS (>480 nm) by Co catalysts (absorbance: **Co–N5**, <445 nm; **Co-PYN5**, <460 nm, see Figure S12) can be excluded,^68^ which also confirms the oxidative quenching pathway in eqs 2, 7, and 8. The oxidative quenching processes are highly dynamic, as significant oxidative quenching was also observed in the time-resolved fluorescence spectroscopic measurements, as exemplified by **IrPPY**-based systems (Figure S36), in which the measured dynamic quenching constants (*k*_q_′) are close to the *k*_q_ values from the steady-state fluorescence spectroscopy.^69^

**Table 3:** Related Data and Calculated Reaction Constants from TA and Steady-State Fluorescence Quenching Experiments

	TA					steady-state fluorescence quenching		
PS	τ_1_ (μs)	*k*_r(BIH)_ (× 10^6^ M^–1^ s^–1^)	*k*_r(BIH)_ (× 10^6^ M^–1^ s^–1^)^a^	*k*_r(Co_–_N5)_ (× 10^9^ M^–1^ s^–1^)	*k*_r(Co-PYN5)_ (× 10^9^ M^–1^ s^–1^)	*k*_q(BIH)_ (M^–1^ s^–1^)	*k*_q(Co_–_N5)_ (× 10^9^ M^–1^ s^–1^)	*k*_q(Co-PYN5)_ (× 10^9^ M^–1^ s^–1^)
**IrPPY**	1.85	80.9	98.0	12.4	11.0	N.A.	17.5	17.0
**IrPPPY**	76.5	3.72	8.51	6.28	26.0	N.A.	13.2 (530 nm)	24.7 (530 nm)
							1.72 (636 nm)	13.9 (636 nm)
							1.85 (690 nm)	20.7 (690 nm)

a The values were determined in the presence of 2.5 v % TEA.

Following the oxidative quenching pathway, two main factors related to Ir PSs are responsible for the photocatalytic performance, including the emission quantum yields of Ir PSs (Φ_em_, eq 2) and the oxidative quenching rates (*k*_q,ox_, eq 7). On the one hand, the less populated ^3^MLCT and the non-emissive nature of ^3^IL make the Φ_em_ value of **IrPPPY** much lower than that of **IrPPY**. On the other hand, the quenching rates between the excited Ir PSs and Co catalysts have been estimated by the Stern–Volmer plots (eq 9), and the related data are listed in Table 3. Among the four pair-wise combinations, first for **IrPPY**, the *k*_q(**Co–N5**)_ and *k*_q(**Co-PYN5**)_ (1.75 vs 1.70 × 10^10^ M^–1^ s^–1^) values at the ^3^MLCT emission are very close, suggesting that the similar quenching abilities between the two Co catalysts with **IrPPY** are consistent with the TA results. This similarity also infers that the CH−π interactions between **IrPPY** and **Co-PYN5** did not achieve a faster electron communication. More importantly, in the systems with **IrPPPY**, the *k*_q(**Co-PYN5**)_ values are sharply higher than *k*_q(**Co–N5**)_ for both the ^3^IL and ^3^MLCT states of **IrPPPY**. It is notable that the quenching by **Co–N5** at ^3^IL emission is slower than the one at ^3^MLCT by nearly a magnitude (1.72/1.85 vs 13.2 × 10^9^ M^–1^ s^–1^ for ^3^IL vs ^3^MLCT) whereas the *k*_q(**Co-PYN5**)_ values of both triplet states are comparable (13.9/20.7 vs 24.7 × 10^9^ M^–1^ s^–1^ for ^3^IL vs ^3^MLCT). This comparison highlights the ultrafast electron transfer via ^3^IL emission between **IrPPPY** and **Co-PYN5**. Although the electron transfer via ^3^IL emission of **IrPPPY** may be less able to initiate the reduction of **Co-PYN5** for catalysis for the lower energy of the ^3^IL state, the interacted pyrenyl groups should serve as a relay to concurrently propel the electron transfer via the ^3^MLCT emission in the **IrPPPY**/**Co-PYN5** system. This synergistic effect enables the highest *k*_q_ of the **IrPPPY**/**Co-PYN5** system (2.47 × 10^10^ M^–1^ s^–1^) among the four pairs at ^3^MLCT emission, which is almost twice of that of the **IrPPPY**/**Co–N5** system (1.32 × 10^10^ M^–1^ s^–1^). Ultimately, the abovementioned multiple spectroscopic experiments have clearly demonstrated that the co-facial π–π interaction renders the ultrafast electron-transfer rate between **IrPPPY** and **Co-PYN5** and thus the highest activity of the **IrPPPY**/**Co-PYN5** system.

Notably, the *k*_r_ estimated from TA spectroscopy is close to the *k*_q_ at ^3^MLCT emission from steady-state fluorescence quenching measurements (e.g., 2.60 vs 2.47 × 10^10^ M^–1^ s^–1^ for the **IrPPPY**/**Co-PYN5** system), which also suggests that the ^3^MLCT-based electron transfer accounts for the main driving force. The former value should be more suitable to describe the intermolecular, photo-induced electron-transfer rate, *k*_PET_, for its mixing nature of different triplet states. *k*_PET_ can be calculated based on the TA data by multiplying the *k*_r_ with the used quencher concentration, eq 10. With [catalyst] = 0.1 mM, the *k*_PET_ in the **IrPPPY**/**Co-PYN5** system was determined as 2.60 × 10^6^ s^–1^.
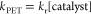
10

Overall, the abovementioned spectroscopic analyses demonstrate that although **IrPPPY** possesses the much lower Φ_em_ and less populated ^3^MLCT than those of **IrPPY**, the intermolecular electron transfer between **IrPPPY** and **Co-PYN5** can still be the fastest to enable a significantly higher activity. Such circumvention demonstrates that the rapid electron transfer facilitated by dynamic π–π interaction plays a crucial role in photocatalysis besides the intrinsic properties of PSs and catalysts. That is, the construction of the co-facial π–π interaction can be a promising strategy to advance the photocatalytic performance to a higher level on the basis of the optimized PS and catalyst.

### Proposed Mechanism

**Figure 9:**
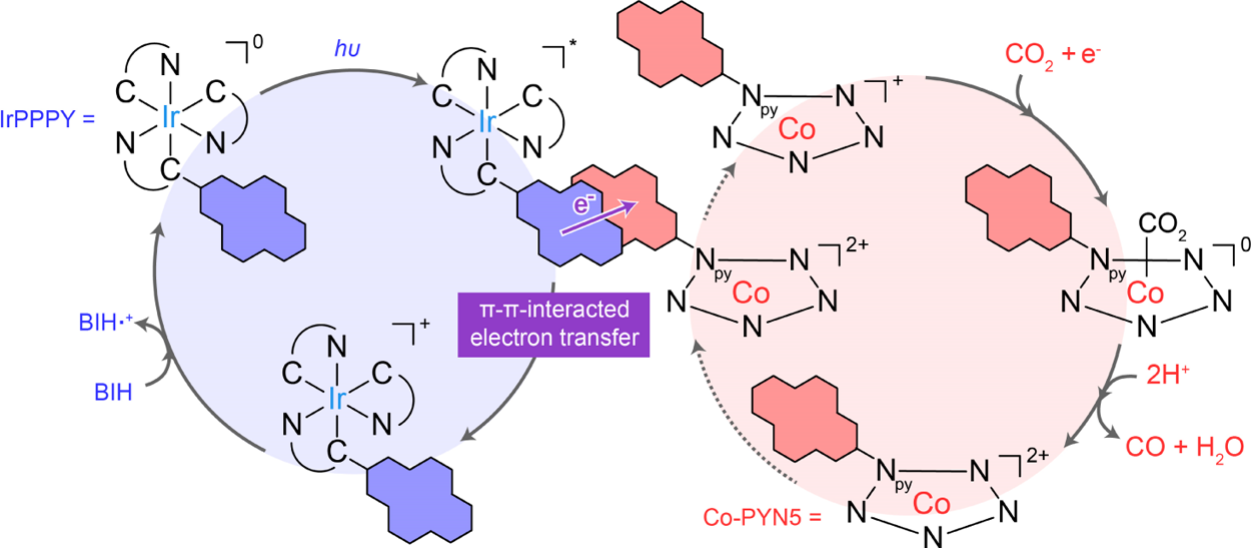
Proposed mechanism. Proposed photocatalytic mechanism for CO_2_-to-CO conversion in the **IrPPPY**/**Co-PYN5** system.

Ultimately, the catalytic mechanism for the optimal **IrPPPY**/**Co-PYN5** system for photocatalytic CO_2_ reduction can be tentatively proposed in Figure 9, according to the abovementioned results and our previous findings on molecular catalysts.^3,20,70,71^ Initially, the photoexcitation of **IrPPPY** generates its excited state. Afterward, the oxidative quenching takes place, in which the photo-induced electron is rapidly delivered to **Co-PYN5** catalyst via the co-facial pyrenyl–pyrenyl π–π interaction, giving rise to the oxidized **IrPPPY** and the one-electron reduced **Co-PYN5**. The former can be recovered to its original form by reacting with BIH. Meanwhile, the reduced **Co-PYN5** will undergo further reduction^43,72^ and bind a CO_2_ molecule and then experience the protonation by the proton source and undergo further reduction to generate CO and pristine **Co-PYN5**. The π–π interaction should also be involved in the second electron-transfer process whereas its further investigation is difficult due to the non-detectable charged states after the first one-electron reduction in TA experiments (Figure 7).

## Concluding Remarks

For the first time, we present here the combined experimental and theoretical characterizations on several π–π interaction modes in purely molecular systems by modifying the PS and the catalyst with pyrenyl groups, which are found to be highly correlated with the catalytic performances. The pair-wise combinations among the pyrene-appended PS and catalyst as well as their prototypes enable the construction of photocatalytic systems with different π interactions, including the co-facial π–π and CH−π interactions. The former interaction has been found to be stronger and presumably more feasible to facilitate the electron transfer, as demonstrated by steady-state and time-resolved spectroscopies, eventually achieving the highest catalytic activity for visible-light-driven CO_2_-to-CO conversion. Remarkably, an optimal Φ(CO) of 14.3 ± 0.8% and a high CO selectivity of 98% can be accomplished, which are comparable to many pioneering photocatalytic systems. However, the Φ(CO) is still lower than some noble-metal-based molecular systems relevant to intermolecular π–π interactions,^50,73^ which can be attributed to the limited catalytic performances of catalysts. Compared to the reported molecular systems with additional interactions between the PS and the catalyst, the affinity of π–π interactions in our work is similar to that of the H-bonding case (10^2^ M^–1^)^8^ but an order of magnitude less than that of coordinative interactions (10^3^ M^–1^).^21^ These differences reveal that the binding strength is relatively weak for non-covalent interactions in contrast to covalent, coordinative interactions, whereas the former can still accelerate the electron-transfer kinetics for improving the photocatalytic CO_2_ reduction. The dynamic π–π interaction should also inhibit the charge recombination (back electron transfer) during photo-induced electron transfer. However, no significant signals of the charged states (formally Ir^IV^ and Co^I^ species) were noticed in the range of 400–700 nm for our ns-TA instrument, which precludes the calculation of back electron-transfer rates^74^ and the comparison with previous covalent-bonding systems. It is also interesting to note that the pyrene substitution of the Ir PS induces a rare dual emission behavior, which enables the distinguishable investigation of electron-transfer processes with different excited states. Finally, we believe our thorough instrumental and computational studies have presented the crucial role of dynamic, non-covalent interaction in the photocatalytic CO_2_ reduction, providing imperative insights for the rational elaboration of molecular-catalyst-based systems and thus the breakthrough on the exploitation of extraordinary photocatalytic systems.

## Experimental Section

### Materials

Co–N5,^72^ Co-PYN5,^43^ [Ir(ppy)_2_(CH_3_CN)_2_]PF_6_,^75^ and BIH^76^ were synthesized by following the literature methods. IrPPY (98%, Aldrich) and other chemicals were commercially available and used without further purification.

### Instruments

^1^H NMR data were gained on a Bruker Avance III instrument (400 MHz). Electrochemical measurements were carried out using an electrochemical workstation (CHI 620E). All potentials were referenced against ferrocenium/ferrocene (Fc^+^/Fc) as an internal standard. Unless otherwise stated, all potentials were footnoted as versus Fc^+^/Fc. The irradiation experiments were carried out with a blue LED light (Zolix, MLED4). Gas chromatographic analysis was conducted on an Agilent 7820A gas chromatograph equipped with a thermal conductivity detector and a TDX-01 packed column, where the oven temperature was held constant at 60 °C, and the inlet and detector temperatures were set at 80 and 200 °C, respectively. The isotopic labeling experiment was conducted under ^13^CO_2_ atmosphere and the gas in the headspace was analyzed by a quantitative mass spectrometer attached to an Agilent 7890A gas chromatograph. The liquid phase of the reaction system was analyzed by an ion chromatograph (Metrohm, 930 Compact IC Flex, Supp 5 anion column, Na_2_CO_3_/NaHCO_3_ aqueous eluent) to detect the presence of formate. UV–vis spectra were collected on an ultraviolet/visible/near-infrared spectrophotometer (PerkinElmer, Lambda 950). The emission quantum yields of Ir PSs were carried out on a fluorescence spectrophotometer (FLSP1000, Edinburgh Instruments Ltd.) equipped with an integrating sphere upon 450/425/405 nm excitation. The lifetime measurements and quenching experiments were conducted on a modular fluorescence life and steady-state fluorescence spectrometer (FLSP980, Edinburgh Instruments Ltd.). TA spectra were measured on an LP980 laser flash photolysis instrument (Edinburgh, UK). All experiments were carried out at room temperature (24 ∼ 25 °C).

## Supporting Information Available

Supplementary experimental details, computational details, supporting figures, supporting tables, and Cartesian coordinates for all optimized structures (PDF)
The cif file of IrPPPY (CIF)

## Supplementary Material

au2c00073_si_001.pdf

au2c00073_si_002.cif

## Notes

Data availability: experimental details, supplementary figures, and data from the ^1^H NMR titration, electrochemistry, spectroscopy, and DFT calculation are available in the Supporting Information. The data with CCDC number, **Co-PYN5** (2011002) and **IrPPPY** (2086643), contain the supplementary crystallographic information for this paper. The data can be obtained free of charge from the Cambridge Crystallographic Data Centre via http://www.ccdc.cam.ac.uk/.
