# Supplementary material for: Co-facial π–π Interaction Expedites Sensitizer-to-Catalyst Electron Transfer for High-Performance CO2 Photoreduction
Source: JACS Au. 2022 Apr 7;2(6):1359–74. doi: 10.1021/jacsau.2c00073 (PMC9241016; doi:10.1021/jacsau.2c00073)
Supplement: Supplementary file 1 — au2c00073_si_001.pdf [file au2c00073_si_001.pdf]

Supporting Information

Co-Facial π-π Interaction Expedites Sensitizer-to-Catalyst Electron Transfer for High-Performance CO_2 Photoreduction

Jia-Wei Wang,[a]+ Hai-Hua Huang,[b]+ Ping Wang,[c]+ Guangjun Yang,[d] Stephan Kupfer,[d] Yanjun Huang,[a] Zizi Li,[a] Zhuofeng Ke,*[b] Gangfeng Ouyang*[a,d,f,g]

[a] KLGHEI of Environment and Energy Chemistry, School of Chemistry, Sun Yat-sen University, Guangzhou 510275, China.

[b] School of Materials Science & Engineering, PCFM Lab, Sun Yat-sen University, Guangzhou 510275, China.

[c] Institute of New Energy Materials and Low Carbon Technology, School of Material Science and Engineering, Tianjin University of Technology, Tianjin 300384, China.

[d] Friedrich Schiller University Jena, Institute of Physical Chemistry, Helmholtzweg 4, 07743 Jena, Germany.

[e] Instrumental Analysis and Research Center, Sun Yat-sen University, Guangzhou, 510275, China.

[f] Chemistry College, Center of Advanced Analysis and Gene Sequencing, Zhengzhou University, Zhengzhou 450001, China.

[g] Guangdong Provincial Key Laboratory of Emergency Test for Dangerous Chemicals, Guangdong Institute of Analysis (China National Analytical Center Guangzhou), Guangzhou, 510070, China.

[+] These authors contributed equally to this work.

Correspondence and requests for materials should be addressed to G.O. (kezhf3@mail.sysu.edu.cn; cesoygf@mail.sysu.edu.cn).

Supplementary Experimental Details

Synthesis of 2-(4-(pyren-1-yl)phenyl)pyridine (pppy ligand). Under a N_2 atmosphere, 1-pyrenylboronic acid (8 mmol, 1.97 g), 2-(4-bromophenyl)pyridine (8 mmol, 1.87 g), K_2CO_3 (24 mmol, 3.31 g) and $Pd(PPh_3)_4$ (10 mol%, 0.8 mmol, 924 mg) were mixed with 100 mL THF/H_2O (v/v = 5/1) in a 200 mL Schlenk flask. The mixture was kept at 70 °C for 36 h under darkness. After cooling to room temperature, the mixture was dried over a rotary evaporator and added with 300 mL of brine, followed by DCM extraction (150 mL × 3). The organic phase was evaporated to dryness and loaded to a silica column chromatography. The pure pppy ligand (colorless needle crystals, ca. 70% yield) was obtained as the second crop of product with petroleum/DCM eluent. ESI-MS(+): $[pppy + H]^+$ (356.4). ^1H NMR (400 MHz, d_7-DMSO), δ 8.73 (d, J = 4.8 Hz, 1H), 8.39 – 8.22 (m, 6H), 8.20 (s, 2H), 8.17 – 8.05 (m, 3H), 8.00 (d, J = 8.0 Hz, 1H), 7.90 (td, J = 7.8, 1.6 Hz, 1H), 7.78 (d, J = 7.9 Hz, 2H), 7.36 (dd, J = 7.4, 5.0 Hz, 1H). Elemental analysis: Calcualted ($C_{27}H_{17}N$), C, 91.24; H, 4.82; N, 3.94; Measured, C, 90.92; H, 4.75; N, 3.95.

Synthesis of IrPPPY. $[Ir(ppy)_2(CH_3CN)_2]PF_6$ (500 mg, 0.7 mmol) and 2-(4-(pyren-1-yl)phenyl)pyridine (710 mg, 2 mmol) were mixed in 20 mL of ethanol. The mixture was heated at 90 °C for 24 h under nitrogen. Precipitate formed during reflux. The reaction mixture was filtered and washed with methanol and hexane. The solid was dissolved in DCM and purified by column using dichloromethane/hexane (v:v = 1:1). 120 mg of pure product was obtained as the first crop yellow solid in the column purification. Slow evaporation of the CH_2Cl_2 or CH_3CN solution of **IrPPPY** under dark, ambient conditions afforded orange block crystals. The crystallization is very easy even some impurity remains. Elemental analysis: Calculated ($IrC_{49}N_3H_{32}$), C, 68.83; H, 3.77; N, 4.91; Measured, C, 68.79; H, 3.85; N, 4.73. ^1H NMR (400 MHz, 1.0 mM,

$CDCl_3$) δ 8.23 (d, J = 9.4 Hz, 1H), 8.14 (t, J = 7.5 Hz, 4H), 8.10-7.82 (m, 10H), 7.65 (d, J = 20.1 Hz, 4H), 7.22 (d, J = 6.6 Hz, 2H), 7.14 (s, 1H), 6.94 (dt, J = 19.5, 6.5 Hz, 6H), 6.84 (s, 1H), 6.67 (t, J = 4.4 Hz, 3H). ESI-MS(+): [**IrPPPY**]$^+$ (854.9).

X-ray Crystallography. Single-crystal X-ray diffraction data were collected at 150 K on an Agilent Technologies Supernova system with Cu/$K\alpha$ (λ = 1.54178 Å) radiation. The empirical absorption corrections were applied using spherical harmonics, implemented in SCALE3 ABSPACK scaling algorithm. The structure was solved using direct method and refined by the full-matrix least-squares method on F^2, which yielded the positions of all non-hydrogen atoms. These were refined first isotropically and then anisotropically. All the hydrogen atoms of the ligand were placed in calculated positions with fixed isotropic thermal parameters and included in the structure factor calculations in the final stage of full-matrix least-squares refinement. All the calculations were performed using the SHELX-97 program[1]. The crystallographic data were summarized in Table S19.

NMR Titration and Determination of Binding Constant. For typical 1H NMR titration experiments, A 0.5 mL solution of host (**IrPPY**/**IrPPPY**, 1.0 × 10^{-3} M) in $CDCl_3$ was titrated with another solution containing the same concentration of host (**IrPPY**/**IrPPPY**, 1.0 × 10^{-3} M) and more concentrated guest (**Co-N5**/**Co-PYN5**, 5.0 × 10^{-2} M) in CD_3CN via an Agilent 0~10 microliter injector. Upon each addition, the solution was manually stirred for 3 min before acquiring the spectrum, which allowed equilibrium to be reached between the host and guest. The addition of small volume (up to 25 μL) of CD_3CN in $CDCl_3$ showed no interference on the proton signals in controlled experiments with no guest.

Binding isotherms for the NMR titration were calculated from a global proton shift analysis using BINDFIT with the shift differences (δ - δ_0) taken to obtain a binding isotherm[2]. The fitting is based on the shift difference between the host-only data and the data at varying guest concentrations. The proton signals used in the fitting belong to those of protons at the pyrenyl groups. The equations used for these analyses are available in the review by Thordarson.[3] In most cases, Nelder-Mead (Simplex) method was used as the optimal search algorithm. The error of binding constant, the root mean square, and the covariance of the fit (variance of the residuals divided by the variance in the data) were used to conclude the models that best describe the binding between host and guest. Specific discussion on the choices of the models is included in the table captions. It should be noted that the non-cooperative mode means that each of the multiple binding process does not interfere with each other, in which the second binding constant (K_{12} or K_{21}) will be equivalent to the first binding constant (K_{11}).

Cyclic Voltammetry. Cyclic voltammetry was performed in a 25 mL gas-tight three-neck flask with a three-electrode system, where a 3 mm glassy carbon disc electrode, a platinum wire auxiliary electrode, and a silver wire pseudo-reference electrode were placed in a 0.1 M tetrabutylammonium hexafluorophosphate ($TBAPF_6$) CH_3CN solution (8.0 mL). Prior to experiments, the working electrode was polished in turn with 0.3 and 0.05 μm Al_2O_3 slurry for 3 min to obtain a mirror surface, then sonicated in water for ~60 seconds to remove debris, and finally rinsed with water. The solution was bubbled with N_2/CO_2 for 15 min prior to each experiment. Scan rate is 100 mV/s.

Spectroelectrochemistry. The combined electrochemistry and UV-Vis spectroscopy were operated in a home-made, gas-tight quartz cell equipped with a ~1 cm^2 glassy carbon rod electrode, a platinum wire auxiliary electrode, and a platinum wire pseudo-reference electrode. The cyclic voltammetry and controlled-potential electrolysis were operated on an electrochemical workstation (CHI 620E) and the in-situ UV-Vis spectra were collected on an ultraviolet visible-near infrared spectrophotometer (Perkin Elmer, Lambda 950). Prior to experiments, the cell with electrodes were sonicated in CH_3CN for ~60 seconds to remove debris, and finally rinsed with CH_3CN. The measured solution contains 20 μM of PS and 0.1 M $TBAPF_6$, which was bubbled with argon for 10 min prior to each experiment. The applied potentials in controlled-potential electrolysis for reduction or oxidation of PSs were based on the one-electron reduction or oxidative waves observed from corresponding CVs in the set-up.

Photocatalytic Experiments. The photocatalytic reduction of CO_2 to CO was conducted in a 17 mL home-made reactor (1.5 cm path) upon successive addition of catalyst, proton source, a CH_3CN solution containing Ir PS and BIH, and finally TEA, under 1 atm CO_2 atmosphere at 293±2 K. PhOH was placed in a N_2-saturated Schlenk flask and heated to liquid state at 70 °C prior to use. After the reaction system purged with CO_2 for 10 min, the photocatalytic reaction was initiated by irradiation under an LED light. The generated gases in the headspace were analyzed by a gas chromatography, and the possible products in the solution were analyzed by an ion chromatograph. The main products are CO and H_2 in the headspace. No formate was detected in the liquid phase.

Determination of Apparent Quantum Efficiency (*Φ*). A reported method[4] was used to determine *Φ*. A typical experiment employed a mixture of **Co-PYN5** (0.1 mM), **IrPPPY** (0.1 mM), TFE (4.0 v%), TEA (2.5 v%), and BIH (25 mM) in 4.0 mL CH_3CN as the sample solution for evaluation. The temperature was kept at 25 °C. The light source is an LED light (λ = 450 ± 5 nm, light intensity = 100 $mW\cdot cm^{-2}$, irradiated area is 0.8 cm^2). The total number of incident photons was measured by a Newport light intensity detector. The photon flux was determined to be 3.01×10^{-7} einstein/s.[5] Under

these conditions, the light entering the reaction solution was considered to be fully absorbed by PS. The 0.5 h of light irradiation is consistent with the total number of photons $n_p = 5.4 \times 10^{-4}$ einstein.

The Φ was evaluated by the Equation S1[6] for two-electron reduction of CO_2.

$$\Phi = n\,(\mathrm{CO}) / n_p \qquad \text{(S1)}$$

A typical Φ of 3.3% was determined by the measured n (CO) = 19.0 μmol by GC-TCD after 0.5 h of irradiation (Table S15, entry 1).

Nanosecond TA Spectroscopy. Nanosecond transient absorption spectra were measured on the LP980 laser flash photolysis instrument (Edinburgh, UK). The pump beam was generated from a tunable laser: Opolette HE 355 LD+UV laser system from OPOTEK (355 nm/410-700 nm, 20/100 Hz, > 4.3 mJ per pulse, and 1 kHz repetition rate). Output pulse of 355 nm from the regenerative amplifier was split into two parts with a beam splitter. The transient spectra were acquired in this work over a wide wavelength range (300-1000 nm). The scan range for decay was 100 ns (1 ns resolution) to 1000 μs (10 μs resolution), which is optional. After penetrating the sample, the probe beam was collimated to focus into a fiber-coupled spectrometer and detected at a frequency of 1 kHz. The intensity of the pump pulse used in this experiment was controlled by a variable neutral-density filter wheel. The delay between the pump and probe pulses was controlled by a motorized delay stage. The pump pulses were chopped by a synchronized chopper at 500 Hz. The pump pulse was kept in a weak regime where the excitonic annihilation effect can be neglected.

After obtaining the lifetime decay diagram of a certain wavelength, the lifetime was fitted using the Tail Fitting method (Range: start from the top point that upon excited to the base point that contour to before excited; Fitting function: exponential function; Index number: singlet to triplet), and then the Map scan of the whole spectrum at the same excited wavelength was carried out (Map processing method: select the number of bars for equidistant distribution, moderately smoothing).

Determination of Second-Order Reaction Rate (k_r). A reported method[7] was followed to calculate the k_r, where the lifetime of reduced Ir PS in the presence of quencher was measured and fitted to the following Equation S2,

$$\frac{\tau_1}{\tau} = 1 + k_r \tau_1 [\mathrm{Q}] \qquad \text{(S2)}$$

in this equation, τ_1 and τ are the lifetimes in the absence and presence of the quencher, and [Q] is the concentration of the quencher.

Determination of Quenching Rates. A reported method[6] was followed to calculate the apparent rate of bimolecular quenching (k_q) in the steady-state measurements, where the fluorescence intensity of photo-excited Ir PS in the presence of quencher was measured and fitted to the Stern-Volmer formula as Equation S3,

$$\frac{I_0}{I} = 1 + K[\mathrm{Q}] = 1 + k_q\tau_0[\mathrm{Q}] \quad \text{(S3)}$$

in this equation, I_0 and I are the fluorescence intensity values in the absence and presence of the quencher, K is the Stern-Volmer constant for dynamic quenching, τ_0 is the lifetime of the excited state without quencher, and [Q] is the concentration of the quencher.

In the time-resolved fluorescent quenching experiments, the calculation equation should be

$$\frac{\tau_0}{\tau} = 1 + k_q{}'\tau_0[\mathrm{Q}] \quad \text{(S4)}$$

in which k_q' is the dynamic quenching rate.

Determination of Rate Constants of Photo-Induced Electron Transfer (k_{PET})[8]. The values of k_{PET} were calculated based on the TA data, by multiplying the k_r with the used quencher concentration ([catalyst] = 0.1 mM), as indicated by Equation S5.

$$k_{\mathrm{PET}} = k_r[\text{catalyst}] \quad \text{(S5)}$$

Computational Details. All quantum chemical simulations addressing the intermolecular interactions among the Ir PSs and the Co catalysts were performed with the Gaussian 09 D.01[9] software package. Geometry optimizations were carried out at the PBE-D3(BJ)[10-12]/BSI (BSI designated the basis set combination of LanL2DZ[13] for Ir and Co atoms and 6-311G(d,p) for nonmetal atoms) level with the SMD implicit solvent model[14] (solvent is acetonitrile). The PBE functional has been successfully applied into the geometry optimization of the face-to-face metal-tetraphenylporphyrin with D3 empirical dispersion correction.[15] Similar to the porphyrin case, the application of PBE functional here is useful for the study on the weak interaction between transition metal complexes, which should be applicable to our case. Frequency analysis for the optimized structures was performed at the same level to characterize them to be the minimal. The energy results were further refined at PBE0-D3(BJ)[11, 16, 17]/BSII (BSII designated the basis set combination of Lanl2DZ[13] for Ir and Co atoms and 6-311+G(d,p) for nonmetal atoms) level with the SMD implicit solvent model (solvent is acetonitrile). It should be noted that the frequency calculations were carried out with the ideal gas model (1 atm, 298.15 K, 24.5 L mol^{-1}), so a concentration correction of 1.9 kcal mol^{-1} was applied to obtain the free energy in the standard 1 M solution. The

reduced density gradient (RDG) isosurfaces were calculated by Multiwfn 3.8[18] and drawn by VMD[19] program. The 3D optimized structures in this paper were drawn by CYLview visualization program.[20]

Furthermore, quantum chemical simulations were performed to elucidate the excited state properties and the photo-induced relaxation channels of **IrPPPY**. Therefore, all following quantum chemical calculations, if not pointed otherwise, were performed utilizing the Gaussian 16[21] program. The ground state equilibrium structure and electronic properties of the Ir(III) complex **IrPPPY** were obtained using DFT utilizing the B3LYP[22] exchange correlation (XC) functional. The def2-SVP[23, 24] basis set as well as the respective core potentials were applied for all atoms. Vibrational analysis was carried out subsequently for the optimized ground state structure to verify that a minimum on the potential energy (hyper-)surface (PES) was obtained. Effects of interaction with the solvent CH_3CN: $\varepsilon = 35.69$, $n = 1.8069$) were taken into account by the solute electron density (SMD) variant of the integral equation formalism of the polarizable continuum model (IEFPCM).[14, 25, 26] All calculations were performed including D3 dispersion correction with Becke-Johnson damping (D3BJ).[17]

Thereafter, time-dependent DFT (TDDFT) calculations were performed using the same computational protocol to assess exited state properties, *i.e.* excitation energies, oscillator strengths, transition dipole moments and electronic characters of the 100 lowest excited singlet states as well as of the 20 lowest triplet states within the Franck-Condon region. Implicit solvent effects (CH_3CN) were considered for excited state properties. For the calculations of excitation energies, where only the fast reorganization of the solvent is important, the non-equilibrium procedure of solvation was used. This computational protocol was already successfully applied to illustrate the ground and excited state properties of structurally closely related Ir(III) complexes.[27-29]

To evaluate scalar-relativistic effects on the various excited states within the Franck-Condon region, calculations were performed utilizing ORCA 5.0,[30] *i.e.*, employing the scalar-relativistic zeroth-order regular approximation (SR-ZORA).[31] DFT and TDDFT calculations were performed using the B3LYP/G XC functional.[32] The SARC-ZORA-TZVP[33] basis set was utilized for Ir, while all other atoms were described using the respective def2-TZVP basis sets (with the corresponding SARC/J auxiliary basis set).[34] The 50 lowest singlet-singlet and singlet-triplet excitations (spin-free, SF, states) were calculated, while the spin-orbit couplings (SOCs) between these states and the singlet ground state were obtained at the SR-ZORA-TDDFT level of theory – yielding the respective spin-orbit (SO) states. Effects of interaction with CH_3CN were taken into consideration with the conductor-like polarizable continuum model (CPCM).[35]

In addition, the triplet ground state, T_1 ($^3ILCT_{PPPY}$), equilibrium structure - directly accessible from the Franck-Condon region – was obtained using DFT, while excited states; *i.e.* the states T_2 ($^3MLCT_{PPPY}$) and T_3 ($^3MLCT_{PPY}$) – obtained at the TDDFT level of theory – were optimized with the external optimizer pysisyphus[36] that interfaces Gaussian 16 for gradient and energy calculations. Wavefunction overlaps were utilized for the tracking of excited states along the course of the

optimization, as implemented in the wfoverlap program.[37] The equilibrium procedure of solvation IEFPCM was applied for all optimizations.

Supporting Figures

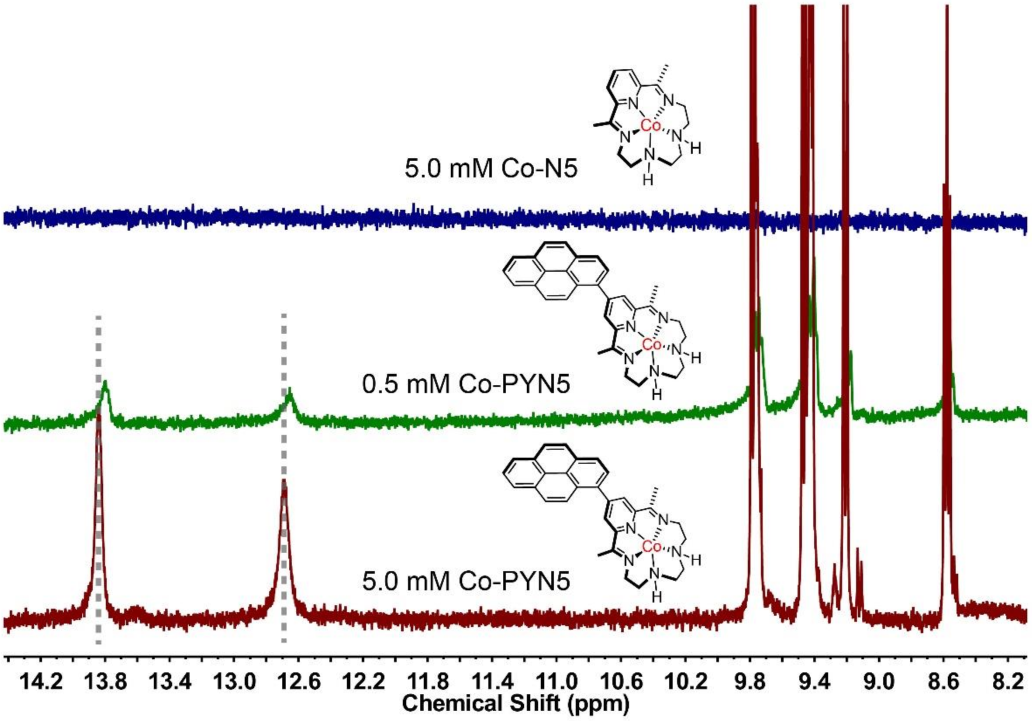

Figure S1. ^{1}H NMR spectra of 5.0 mM **Co-N5** (navy), 0.5 mM **Co-PYN5** (olive) and 5.0 mM **Co-PYN5** (crimson) in CD_3CN.

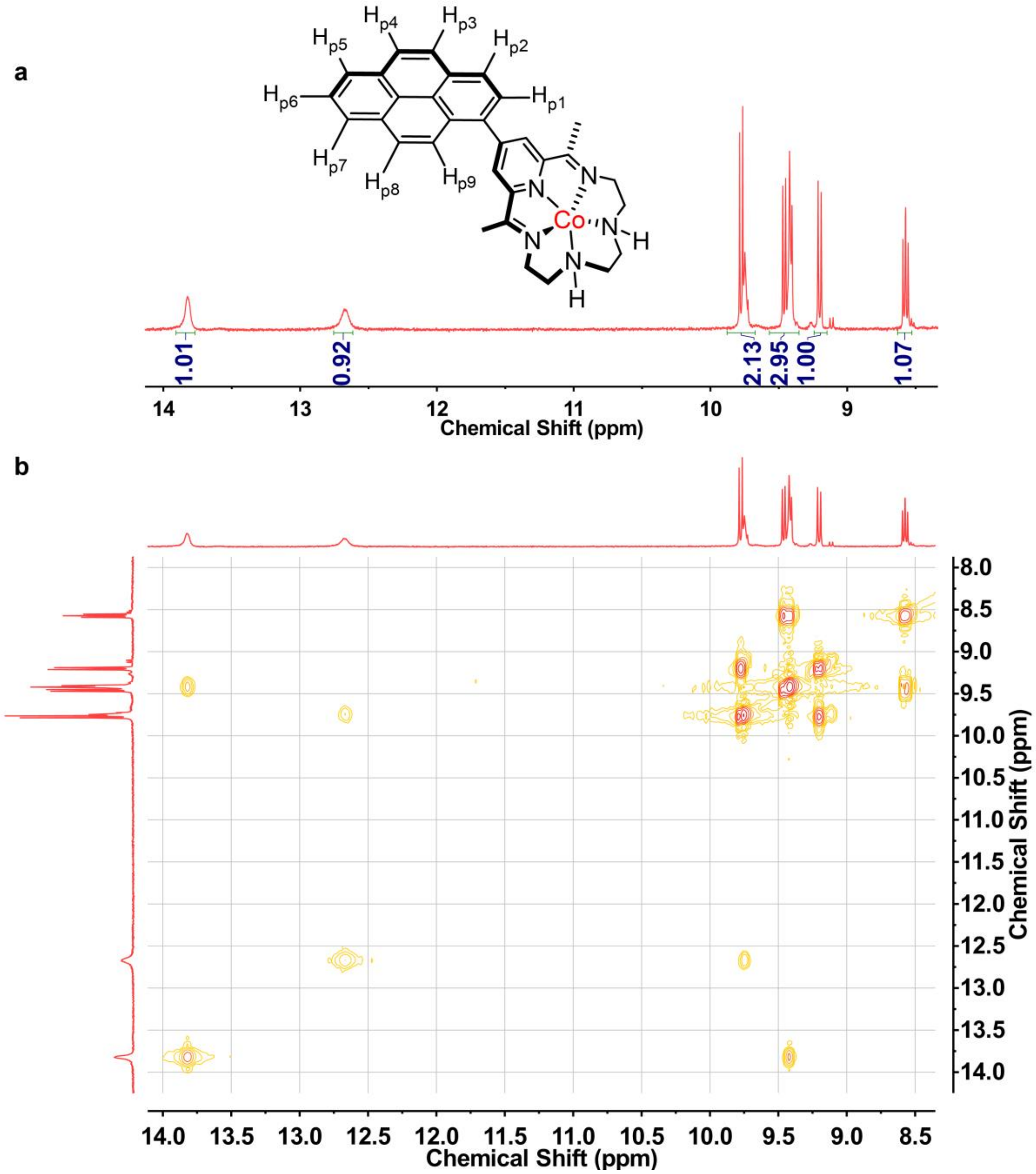

Figure S2. (a) ^1H and (b) ^1H-^1H COSY NMR spectra of 5.0 mM **Co-PYN5** in CD_3CN solution with proton assignments.

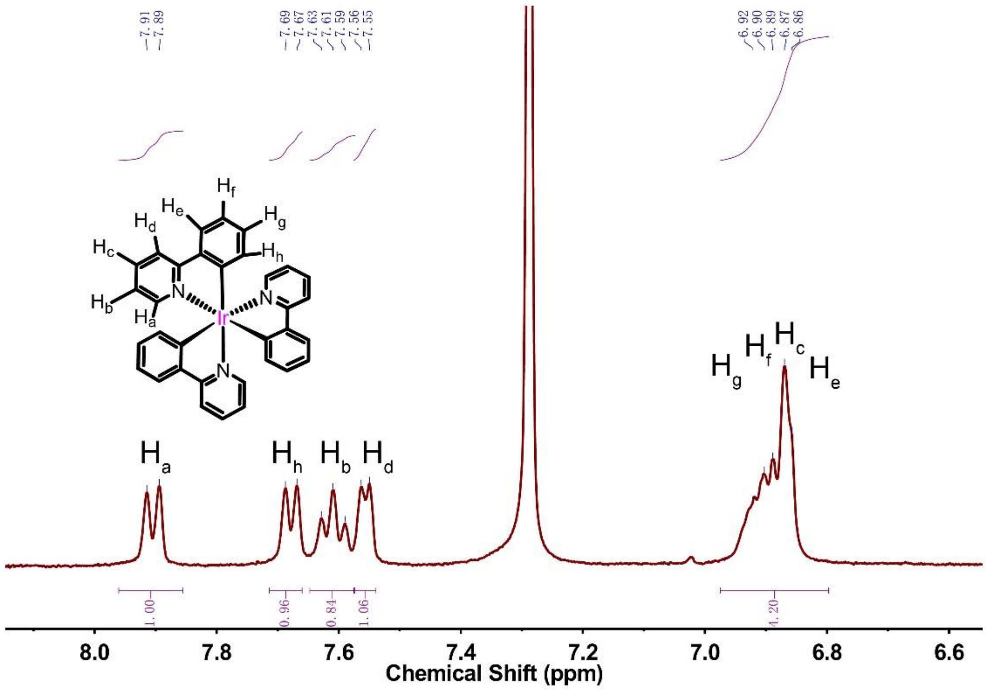

Figure S3. ^{1}H NMR spectrum of 5.0 mM **IrPPY** with indication of proton positions in $CDCl_3$.

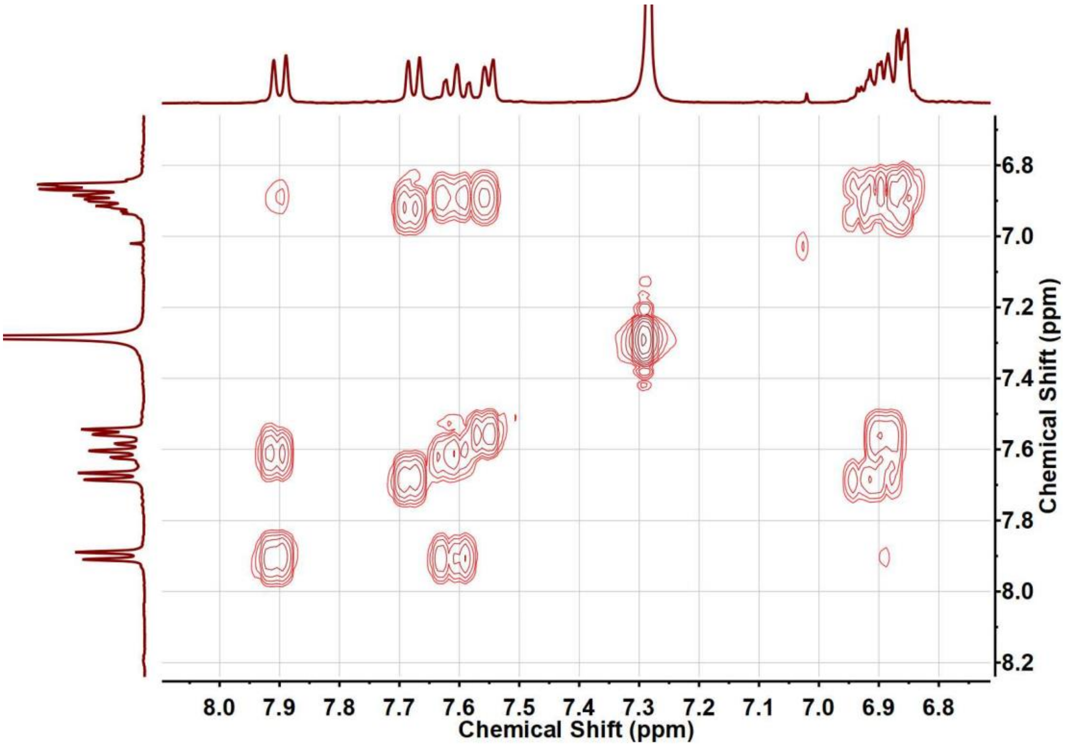

Figure S4. ^{1}H-^{1}H 2D COSY NMR spectrum of 5.0 mM **IrPPY** in $CDCl_3$.

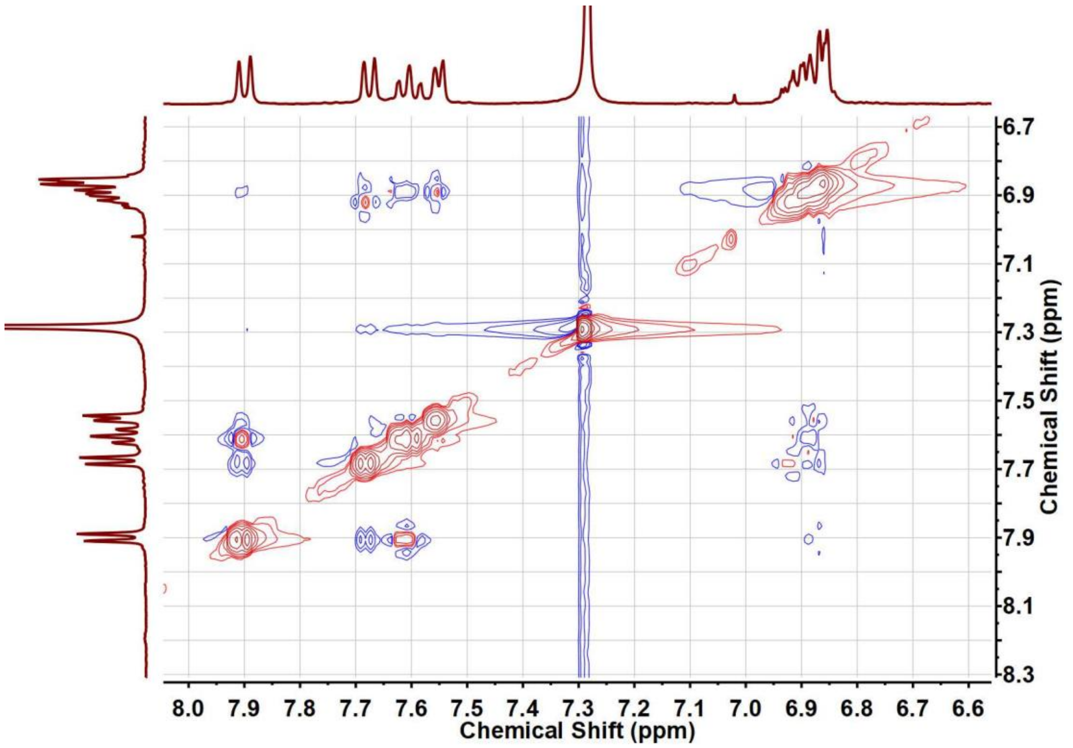

Figure S5. ^{1}H-^{1}H 2D NOESY NMR spectrum of 5.0 mM **IrPPY** in $CDCl_3$.

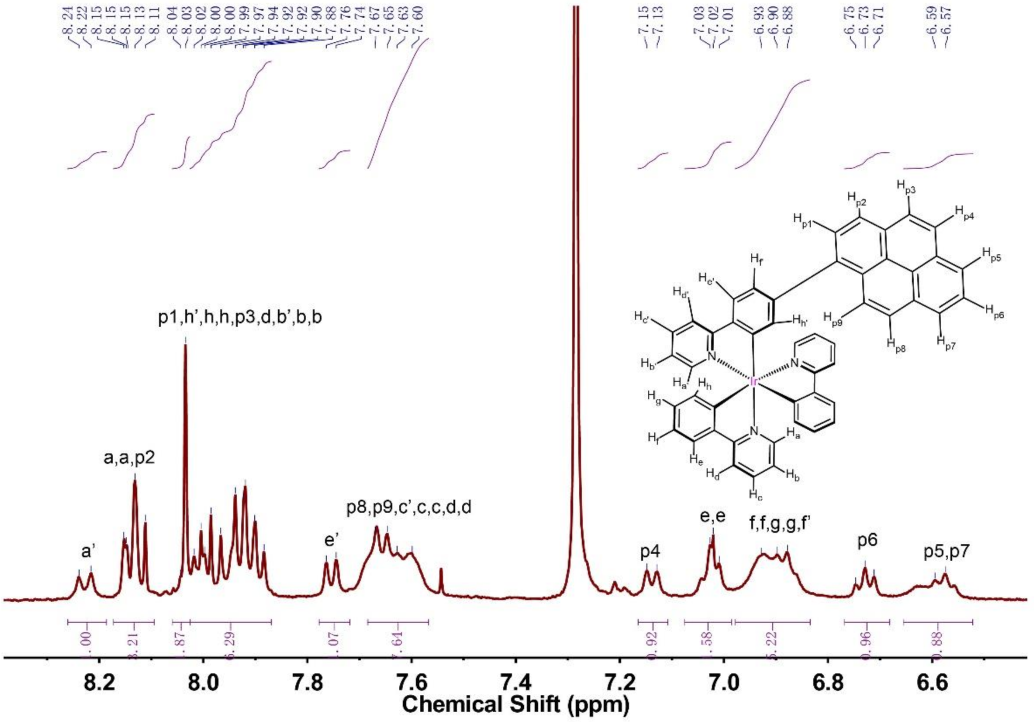

Figure S6. ^{1}H NMR spectrum of 5.0 mM **IrPPPY** with indication of proton positions in $CDCl_3$.

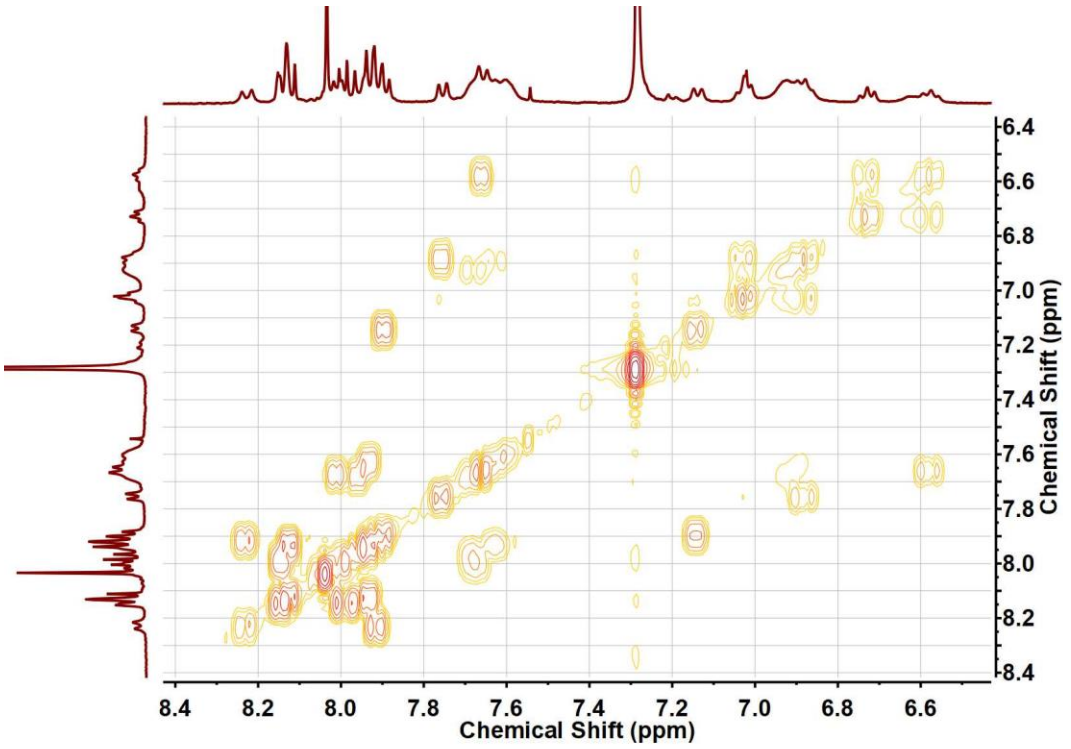

Figure S7. ^{1}H-^{1}H 2D COSY NMR spectrum of 5.0 mM **IrPPPY** in $CDCl_3$.

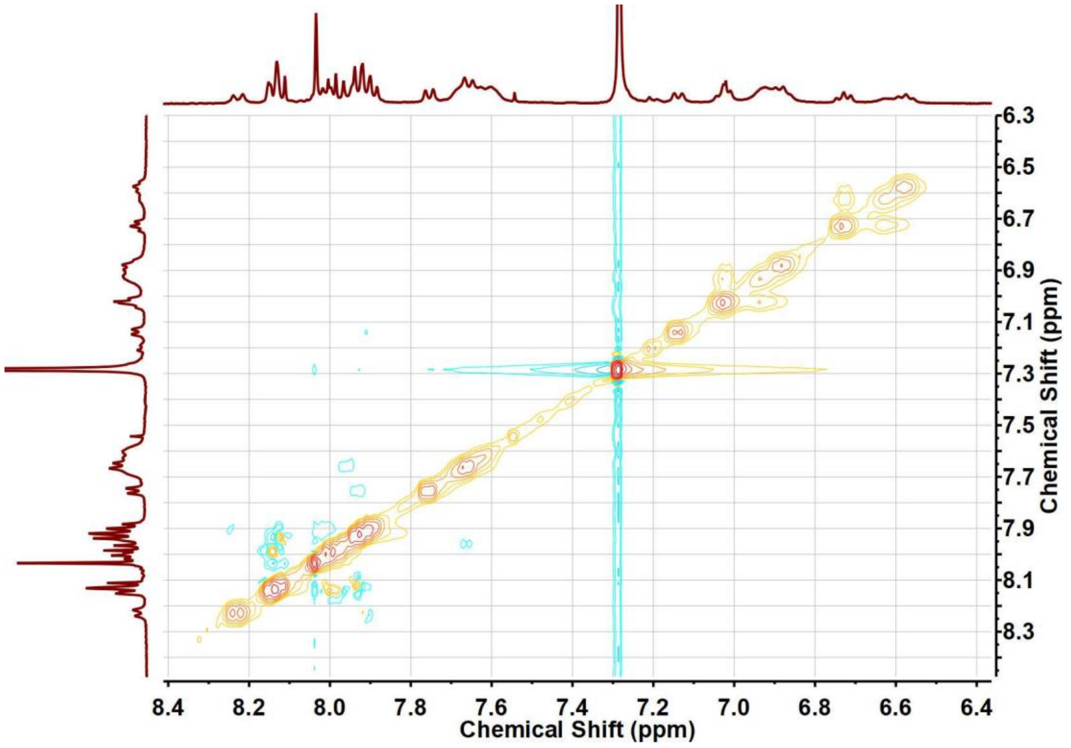

Figure S8. ^{1}H-^{1}H 2D NOESY NMR spectrum of 5.0 mM **IrPPPY** in $CDCl_3$.

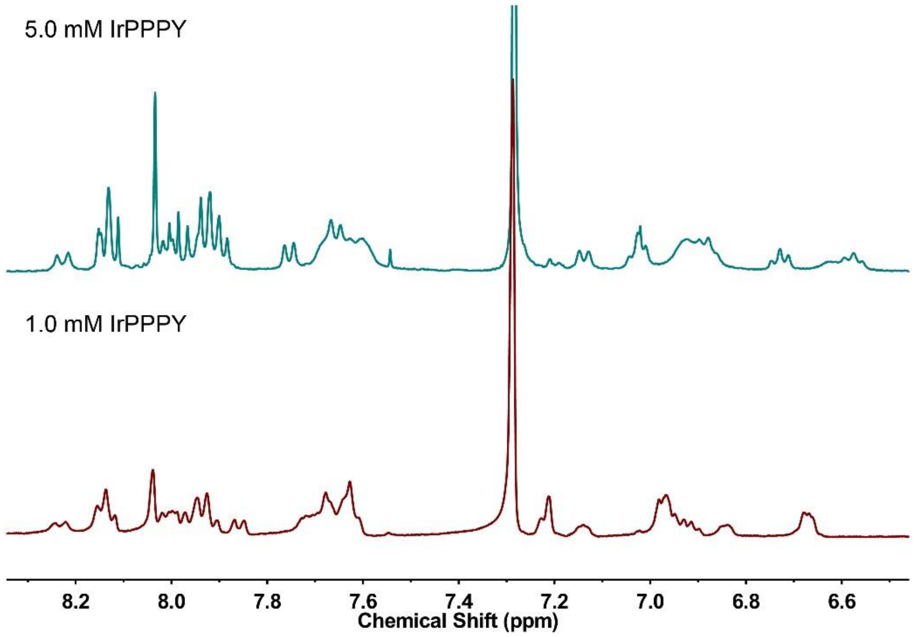

Figure S9. ^{1}H NMR spectra of 5.0 mM (blue) or 1.0 mM (crimson) **IrPPPY** in $CDCl_3$. It can be seen that the less concentrated solution of **IrPPPY** shows a spectrum where the proton signals are shifted toward low field, in which the resolution is not good and the position of the important H_{p3} getting unclear. This is why the assignment was done on a 5.0 mM solution. However, the subsequent NMR titration still requires the use of a relatively dilute solution (1.0 mM) for achieving high guest/host ratio.

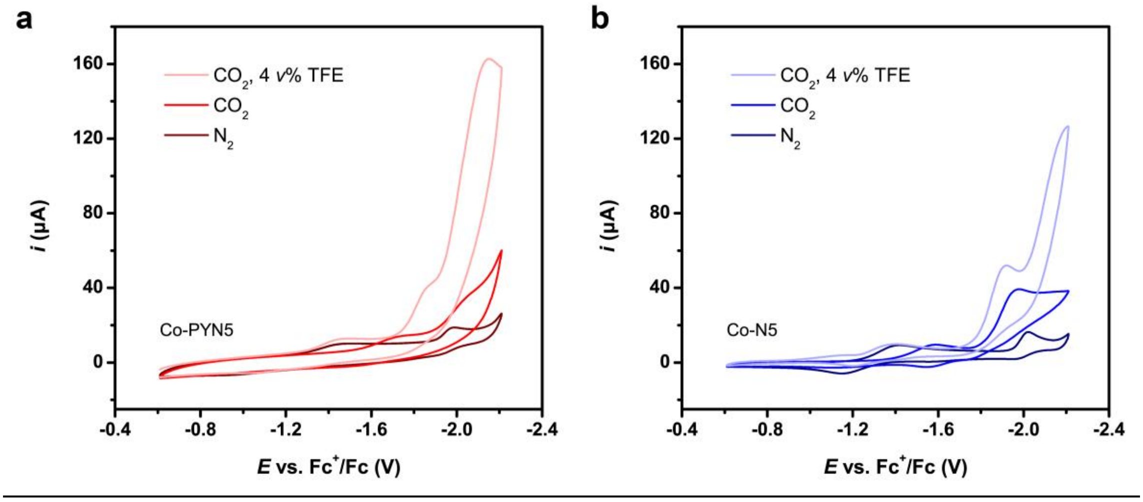

Figure S10. (a) CVs of 0.5 mM **Co-PYN5** in 0.1 M nBu$_4$NPF$_6$ CH_3CN solution under N_2 (deep red), CO_2 (red), CO_2 with 4 v% TFE (pale red). (b) CVs of 0.5 mM **Co-N5** in 0.1 M nBu$_4$NPF$_6$ CH_3CN solution under N_2 (deep blue), CO_2 (blue), CO_2 with 4 v% TFE (pale blue)

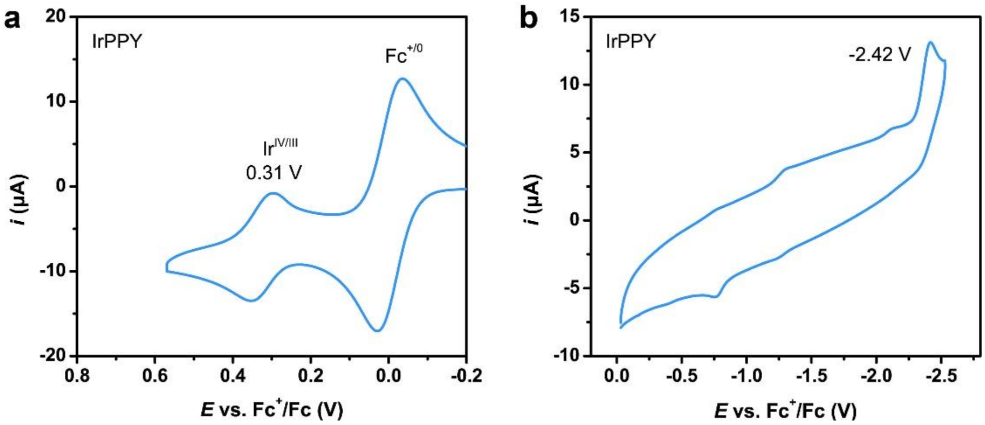

Figure S11. CV of 0.5 mM **IrPPY** in 0.1 M $n\mathrm{Bu_4NPF_6}$ CH_3CN solution under N_2 with Fc as the internal reference.

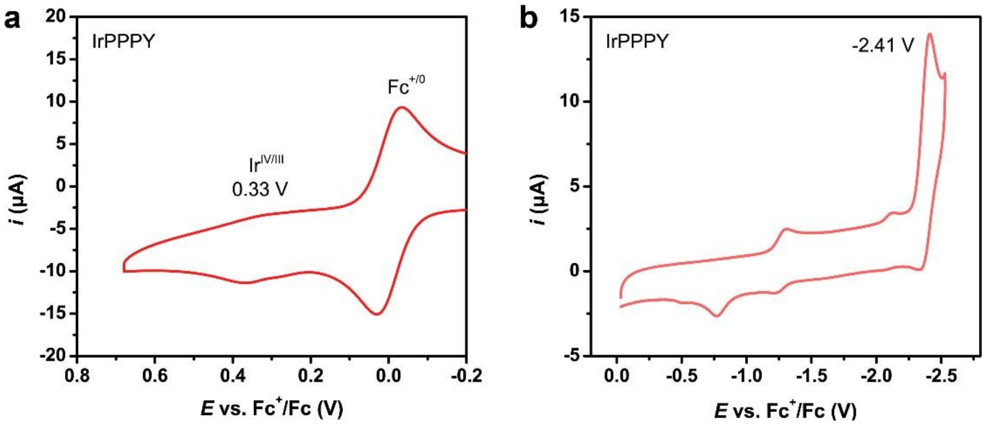

Figure S12. CV of 0.5 mM **IrPPPY** in 0.1 M $n\mathrm{Bu_4NPF_6}$ CH_3CN solution under N_2 with Fc as the internal reference.

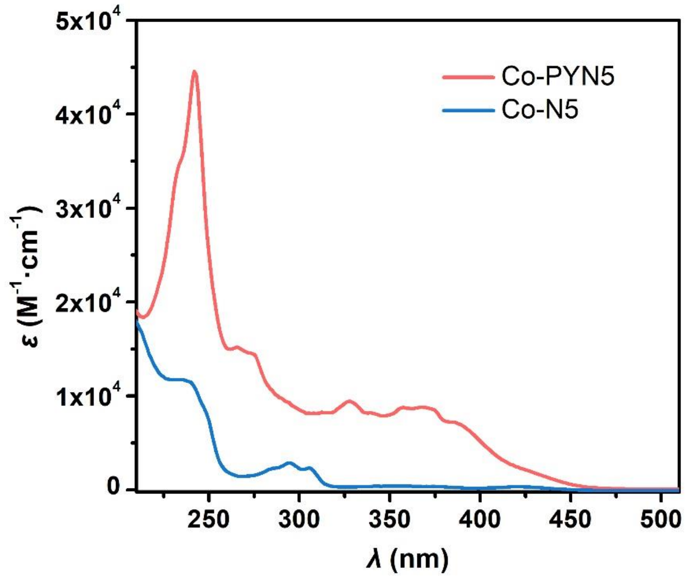

Figure S13. UV-Vis spectra of 50 μM **Co-N5** (blue) or **Co-PYN5** (red) in CH_3CN.

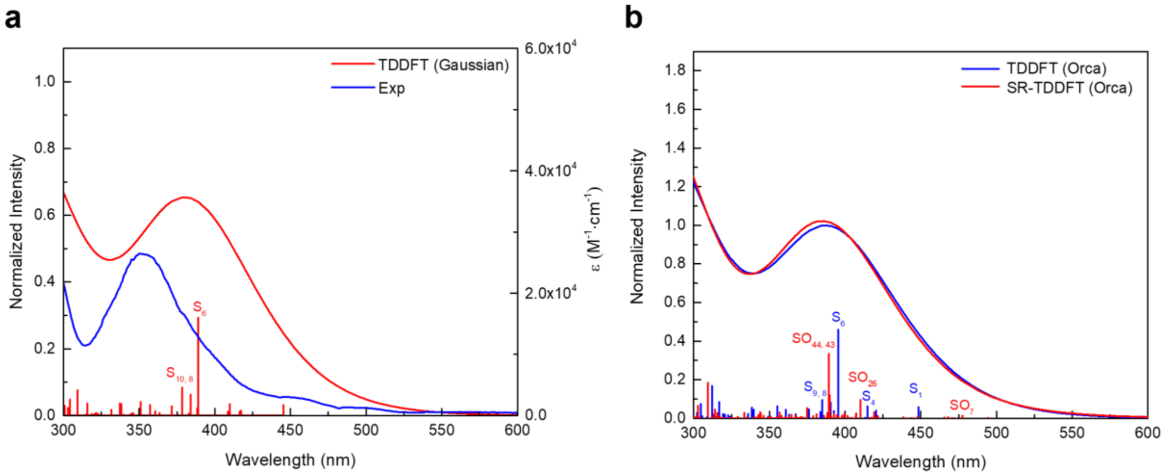

Figure S14. (a) Simulated (red, spin-free) and experimental (blue) absorption spectra of **IrPPPY**. The three main singlet states are indicated, (b) simulated absorption spectra of **IrPPPY** obtained by ORCA 5.0. spectra were obtained by means of excited spin-free singlet states (blue) and spin-orbit states (red), solvent effects (CH_3CN) were incorporated by means of CPCM. Electronic transitions are broadened by Lorentzian functions with a full width at half maximum of 0.333 eV.

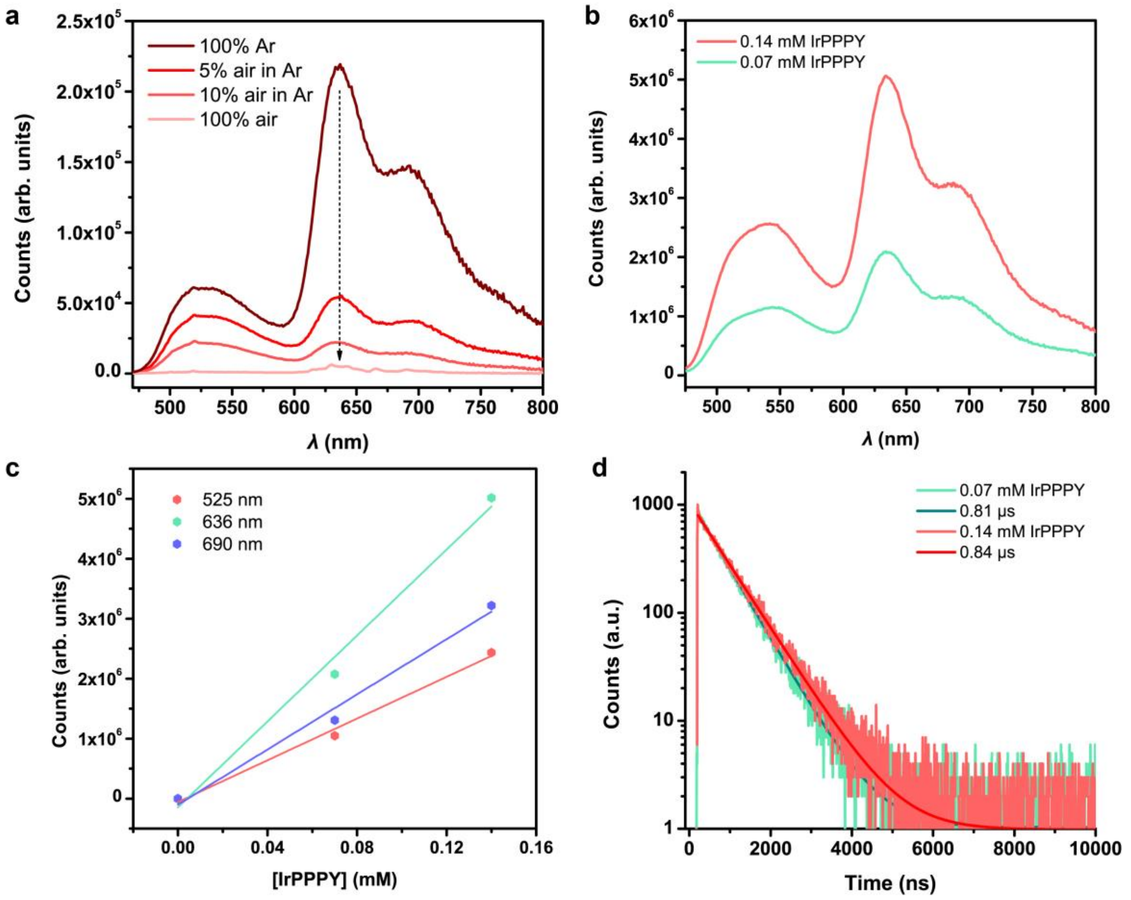

Figure S15. (a) Emission spectra of 50 μM **IrPPPY** in CH_3CN with increasing air ratio in the atmosphere. (b) Emission spectra of 0.14 (red) and 0.07 mM (green) **IrPPPY** in CH_3CN with (c) plots of fluorescent intensity versus [**IrPPPY**] at wavelengths of 525, 636 and 690 nm. (d) Emission lifetimes at 530 nm of 0.14 (red) and 0.07 mM (green) **IrPPPY** in CH_3CN.

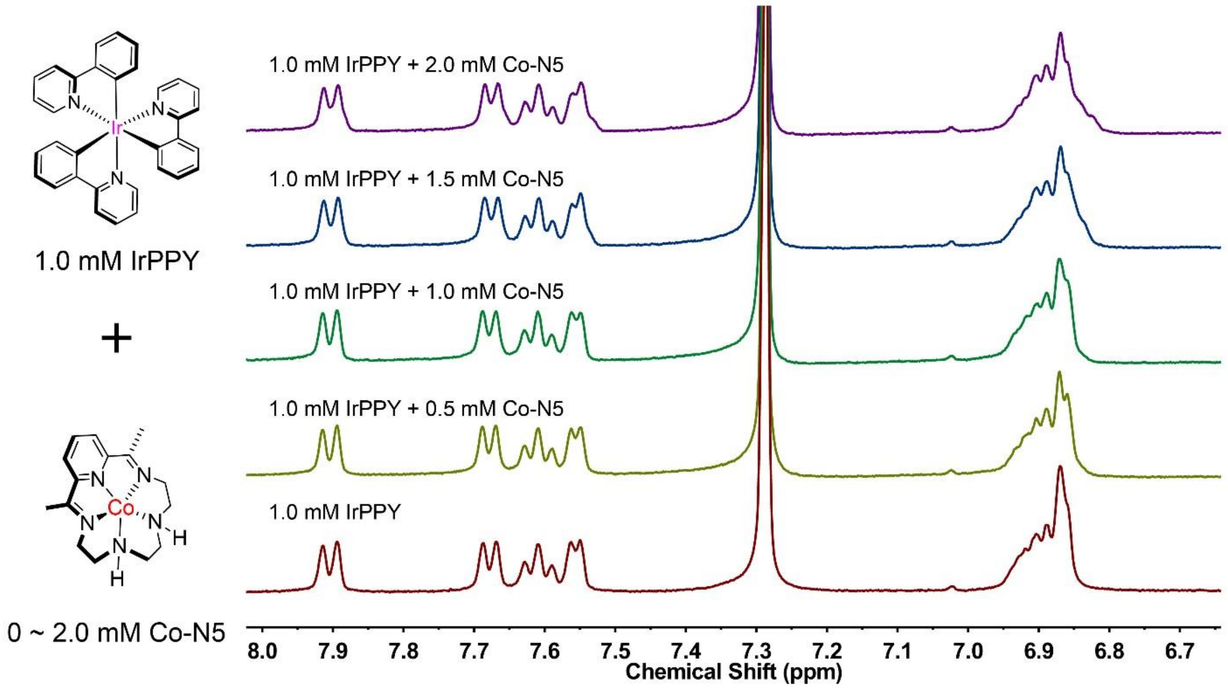

Figure S16. ^{1}H NMR titration of **Co-N5** into the $CDCl_3$ solution of **IrPPY**.

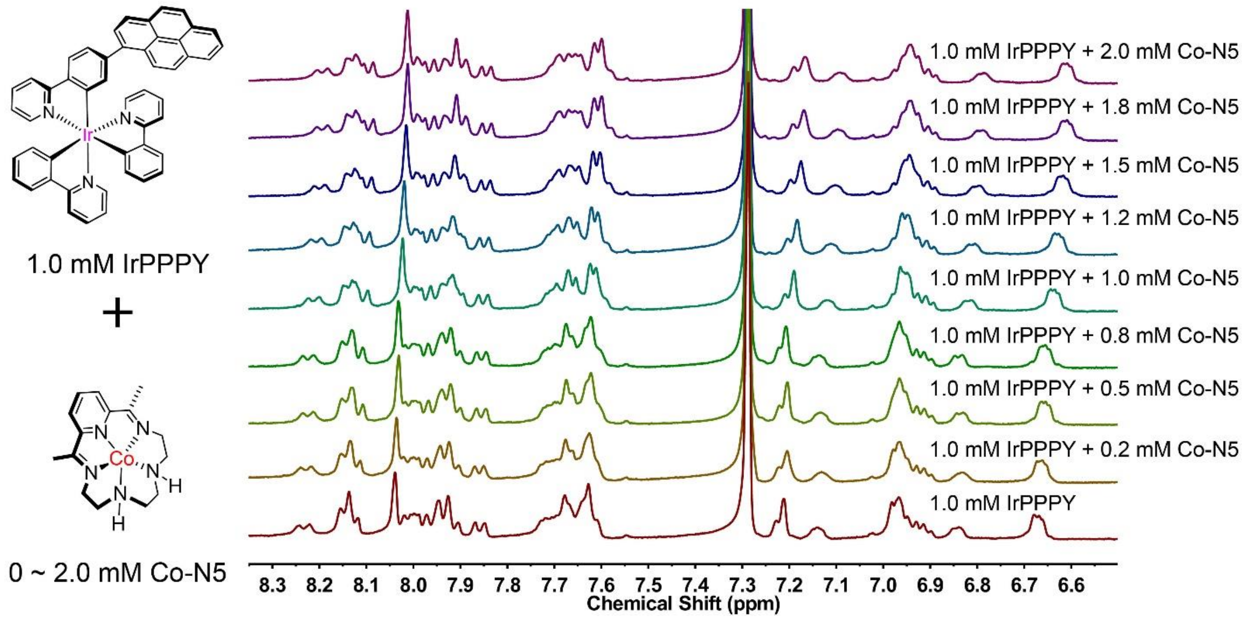

Figure S17. ^{1}H NMR titration of **Co-N5** into the $CDCl_3$ solution of **IrPPPY**.

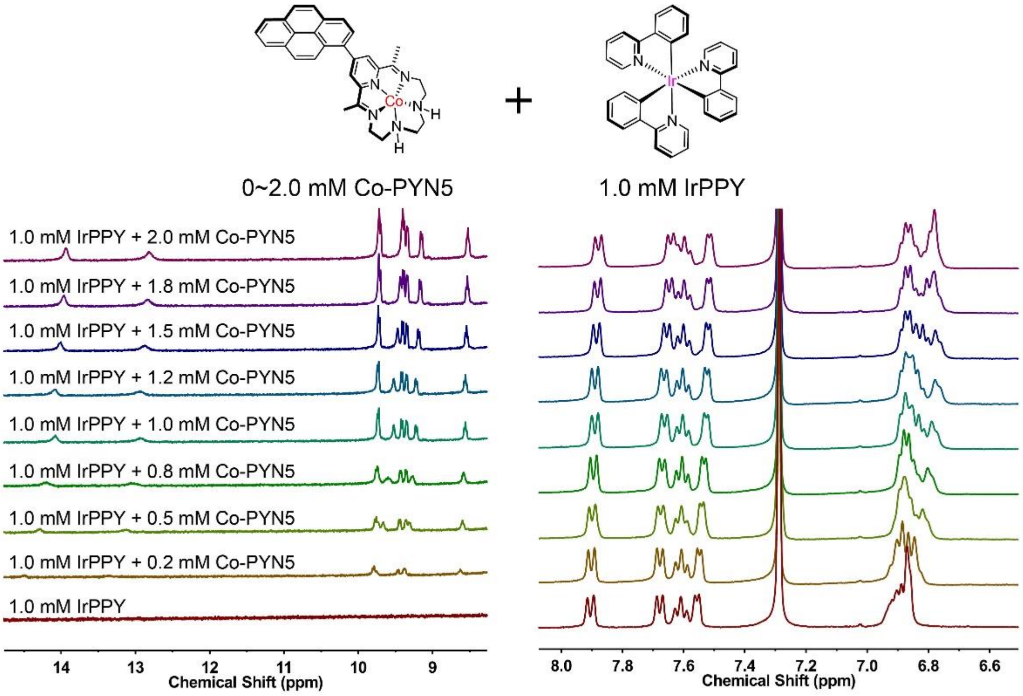

Figure S18. ^{1}H NMR titration of **Co-PYN5** into the $CDCl_3$ solution of **IrPPY**. We considered the shifts should be caused by interaction between **Co-PYN5** and **IrPPY** rather than the paramagnetic Co(II) species, as the proton signals of **IrPPY** showed no shift with addition of **Co-N5** (Figure S15).

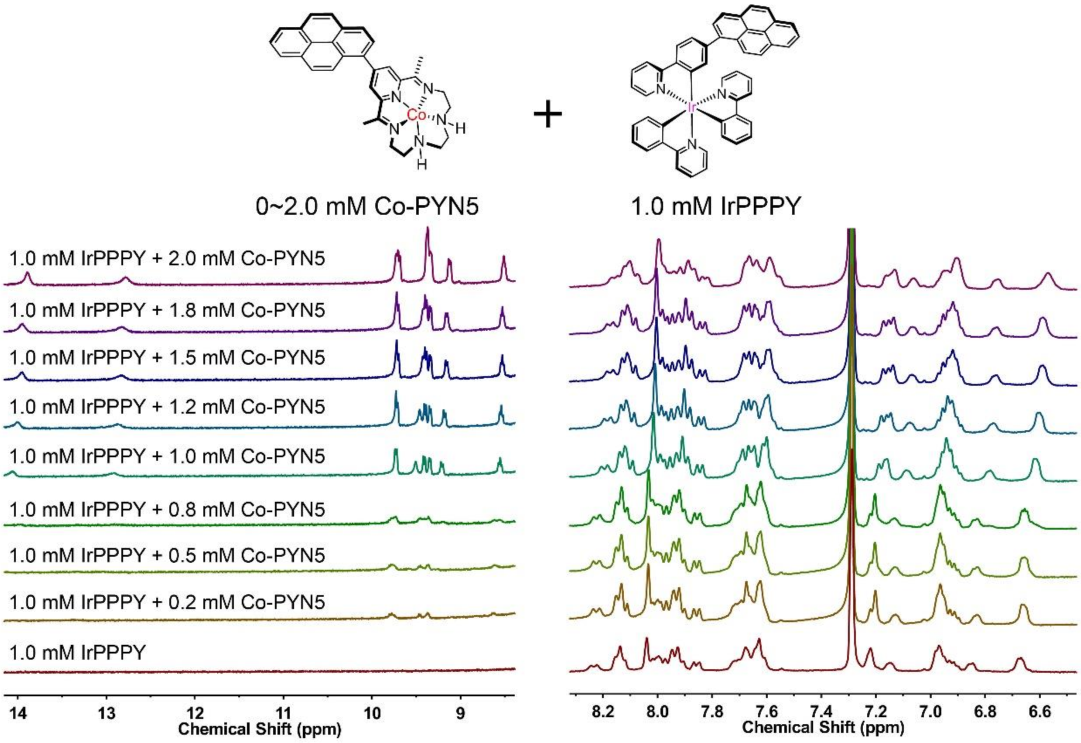

Figure S19. ^{1}H NMR titration of **Co-PYN5** into the $CDCl_3$ solution of **IrPPPY**.

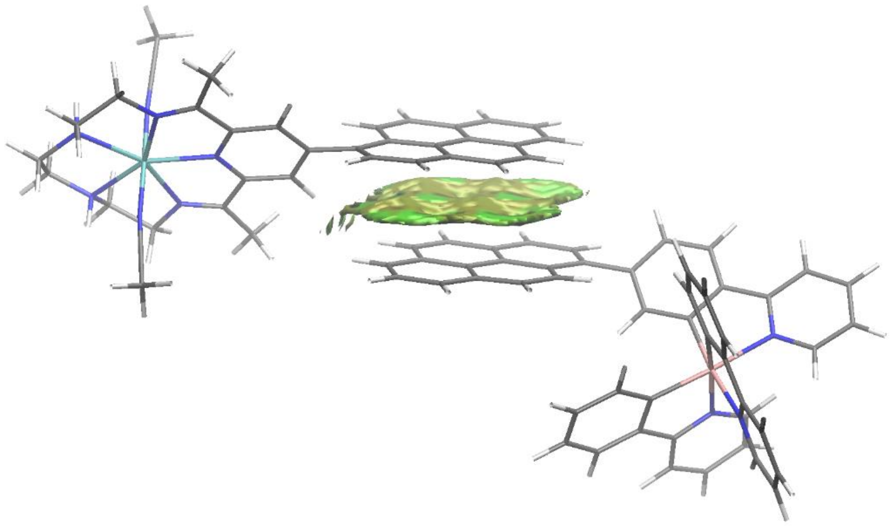

Figure S20. RDG isosurface for **IrPPPY**/**Co-PYN5** couple. Atom color: Ir, pale pink; Co, cyan; C, gray; H, white.

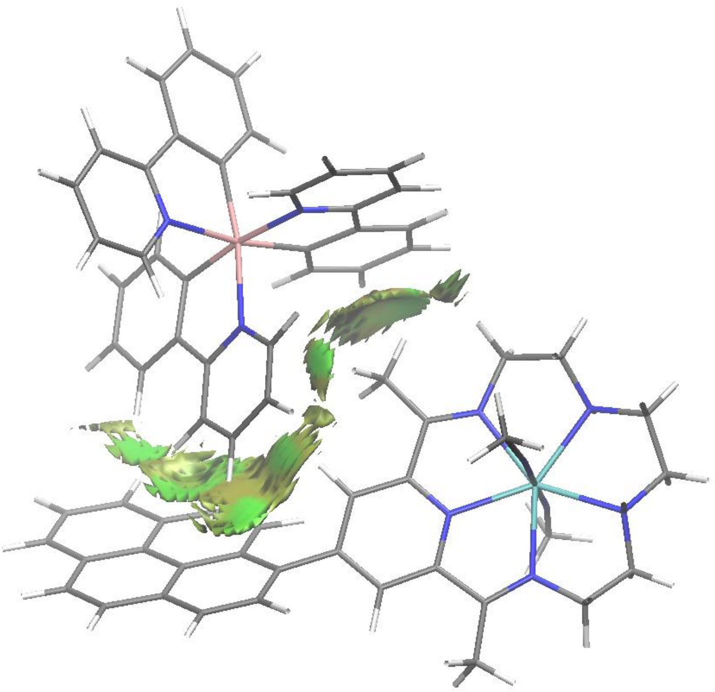

Figure S21. RDG isosurface for **IrPPY**/**Co-PYN5** couple. Atom color: Ir, pale pink; Co, cyan; C, gray; H, white.

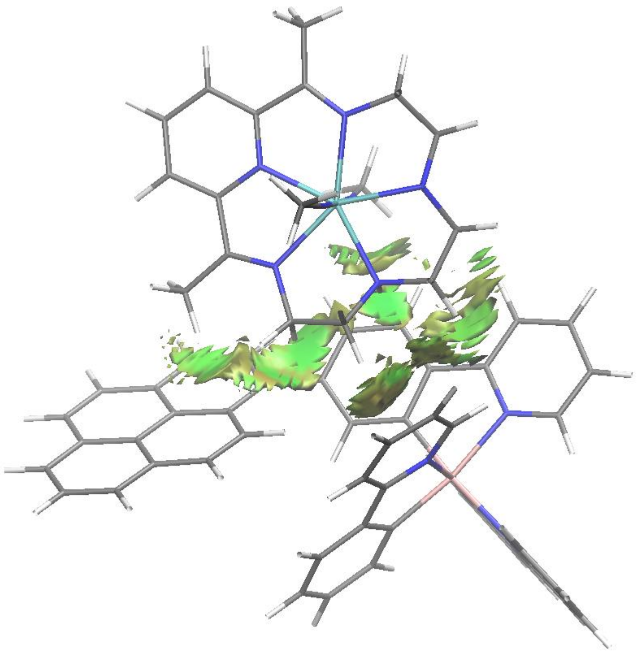

Figure S22. RDG isosurface for **IrPPPY**/**Co-N5** couple. Atom color: Ir, pale pink; Co, cyan; C, gray; H, white.

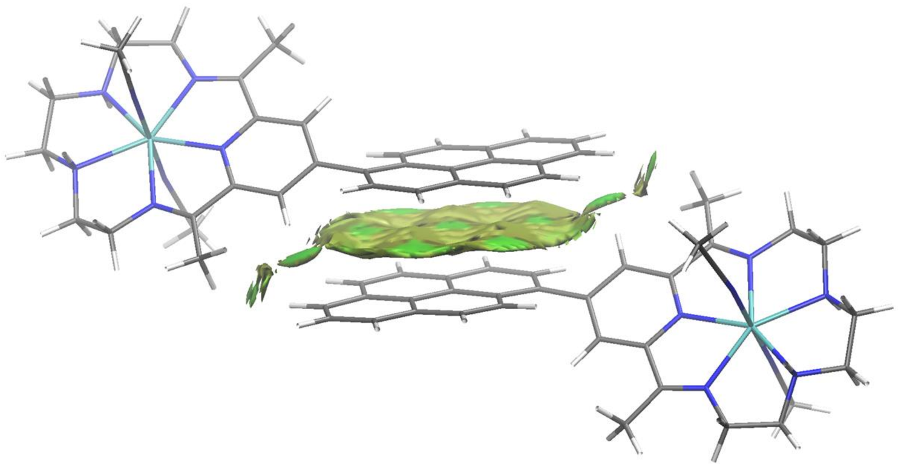

Figure S23. RDG isosurface for **Co-PYN5** dimerization. Atom color: Co, cyan; C, gray; H, white.

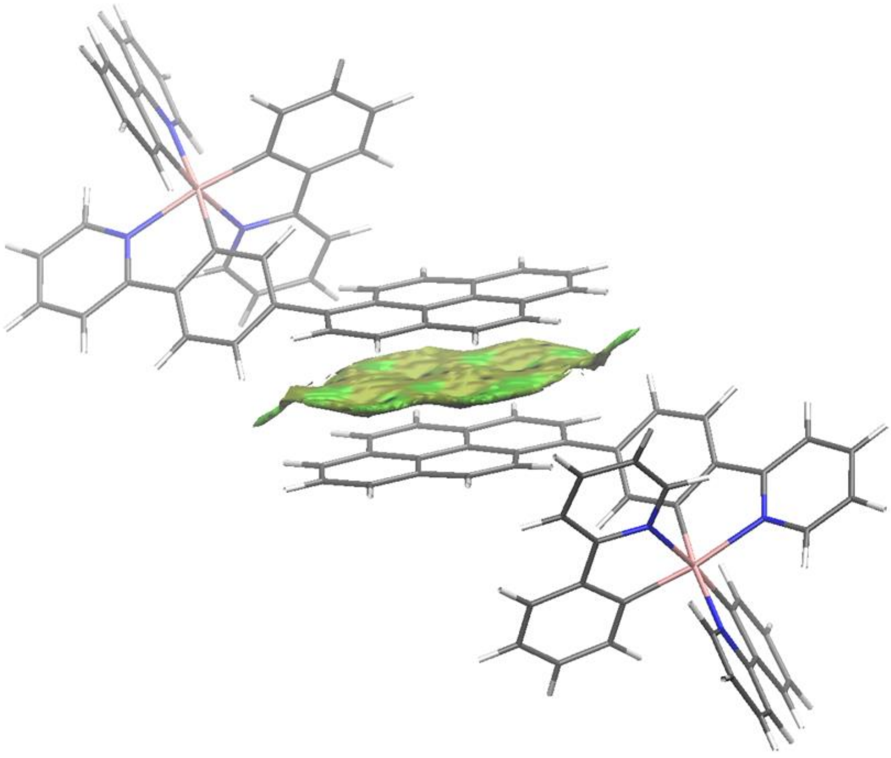

Figure S24. RDG isosurface for **IrPPPY** dimerization. Atom color: Ir, pale pink; C, gray; H, white.

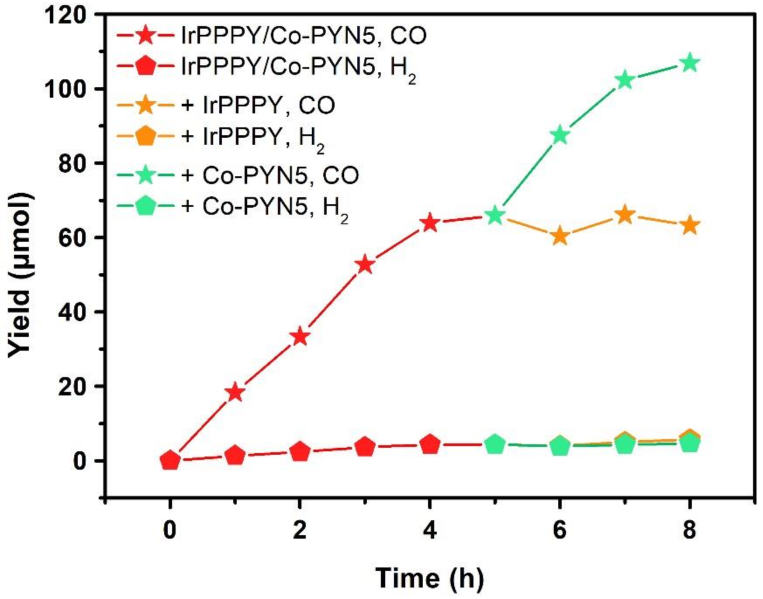

Figure S25. Time profiles of photocatalytic CO (star) and H_2 (pentagon) formation from a mixture of **Co-PYN5** (0.1 mM), **IrPPPY** (0.1 mM), TEA (2.5 v%), TFE (4.0 v%), and BIH (25 mM) in 4.0 mL CH_3CN under 1 atm CO_2. After 5 h, fresh **IrPPPY** (0.4 μmol, orange) or **Co-PYN5** (0.4 μmol, green) was dispersed in 0.5 mL CO_2-saturated CH_3CN and injected in the solution, respectively.

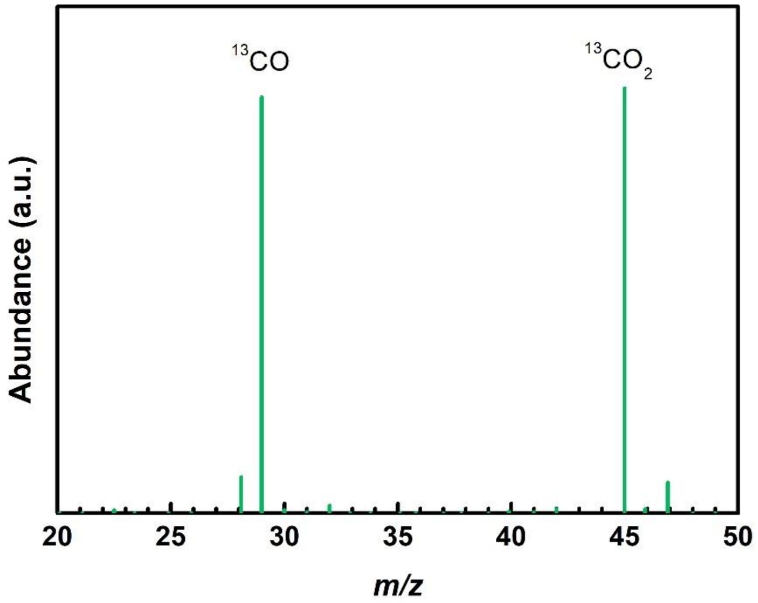

Figure S26. Mass spectra analyses via gas chromatography on the generated gas from a mixture of **Co-PYN5** (0.1 mM), **IrPPPY** (0.1 mM), TEA (2.5 v%), TFE (4.0 v%), and BIH (25 mM) in 4.0 mL CO_2-saturated CH_3CN within 4 h of 450 nm irradiation under 1 atm CO_2.

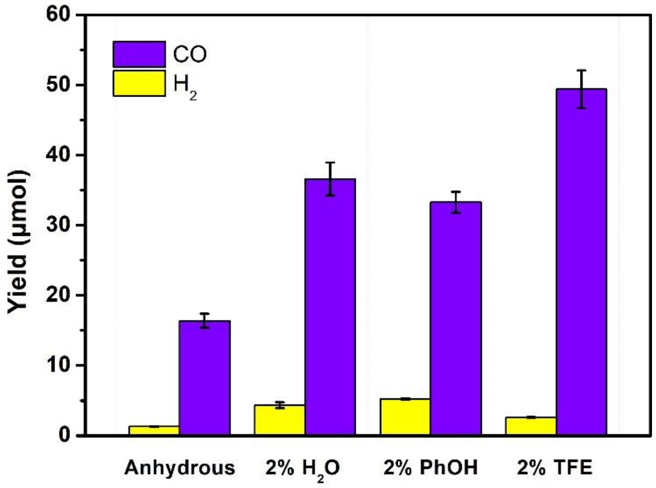

Figure S27. Photocatalytic CO and H_2 yields from a mixture of **Co-PYN5** (0.1 mM), **IrPPPY** (0.1 mM), TEA (2.5 v%), and BIH (25 mM) in 4.0 mL CH_3CN within 4 h of 450 nm irradiation under 1 atm CO_2, in the absence of proton source or the presence of 2.0 v% H_2O, PhOH or TFE.

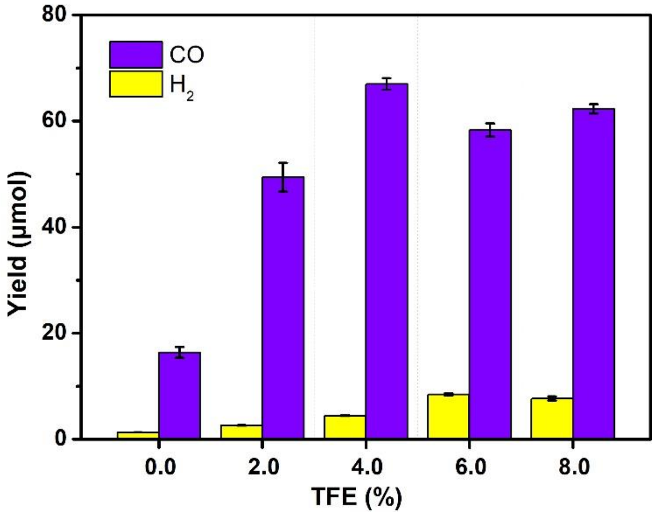

Figure S28. Photocatalytic CO and H_2 yields from a mixture of **Co-PYN5** (0.1 mM), **IrPPPY** (0.1 mM), TEA (2.5 v%), TFE (0, 2.0, 4.0, 6.0 or 8.0 v%), and BIH (25 mM) in 4.0 mL CO_2-saturated CH_3CN within 4 h of 450 nm irradiation under 1 atm CO_2.

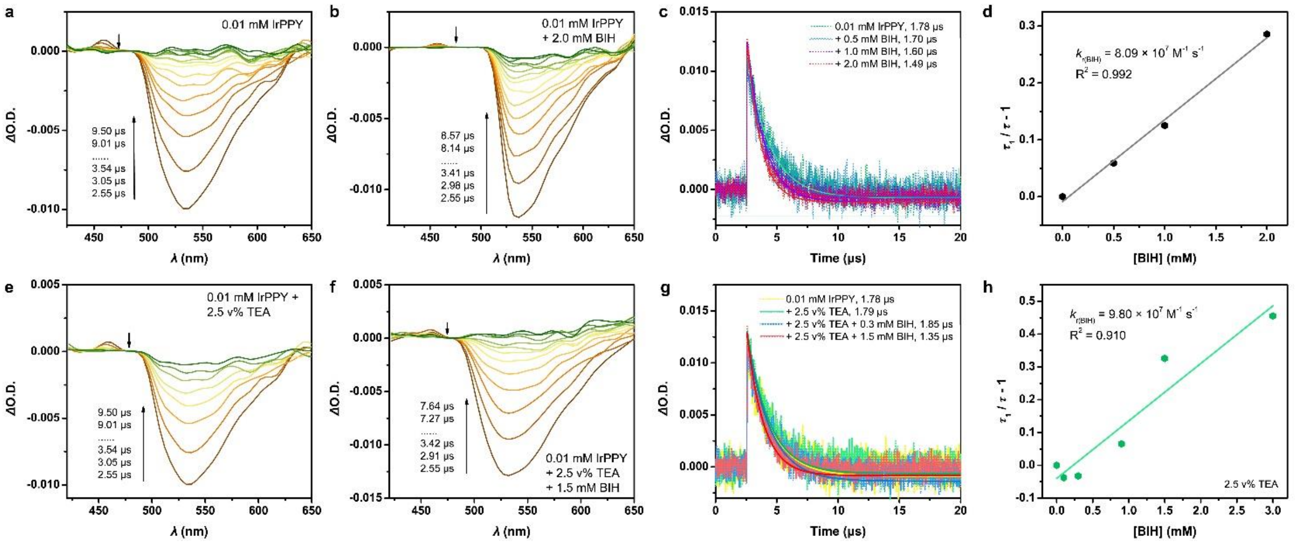

Figure S29. Nanosecond TA spectra of **IrPPY**. (a) 0.01 mM **IrPPY**, (b) 0.01 mM **IrPPY** with 2.0 mM BIH, (c) kinetic traces of **IrPPY** with 0~2.0 mM BIH followed at 520 nm, (d) Plots of (τ_1 / τ - 1) versus the concentration of BIH with linear fitting. (e) 0.01 mM **IrPPY** with 2.5 v% TEA. (f) 0.01 mM **IrPPY** with 2.5 v% TEA and 1.5 mM BIH. (g) kinetic traces of **IrPPY** with 2.5 v% TEA and 0~1.5 mM BIH followed at 520 nm. (h) Plots of (τ_1 / τ - 1) versus the concentration of BIH in the presence of 2.5 v% TEA with linear fitting. The data were collected in Ar-saturated CH_3CN upon excitation at 450 nm.

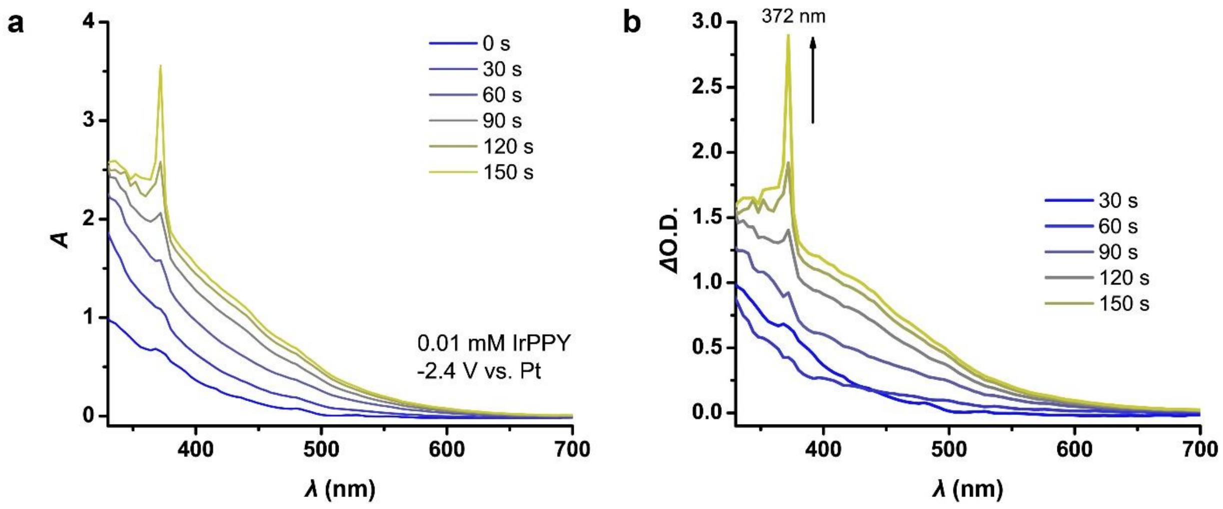

Figure S30. (a) Measured and (b) corresponding differential spectra of UV-vis absorption of 0.01 mM **IrPPY** in the presence of 0.1 M *n*Bu_4NPF_6 upon reduction under N_2. The sharp peak should be induced by the long step during the fast scans (5 nm). The solution was changed from pale yellow to brown, which can be recovered by exposing to air.

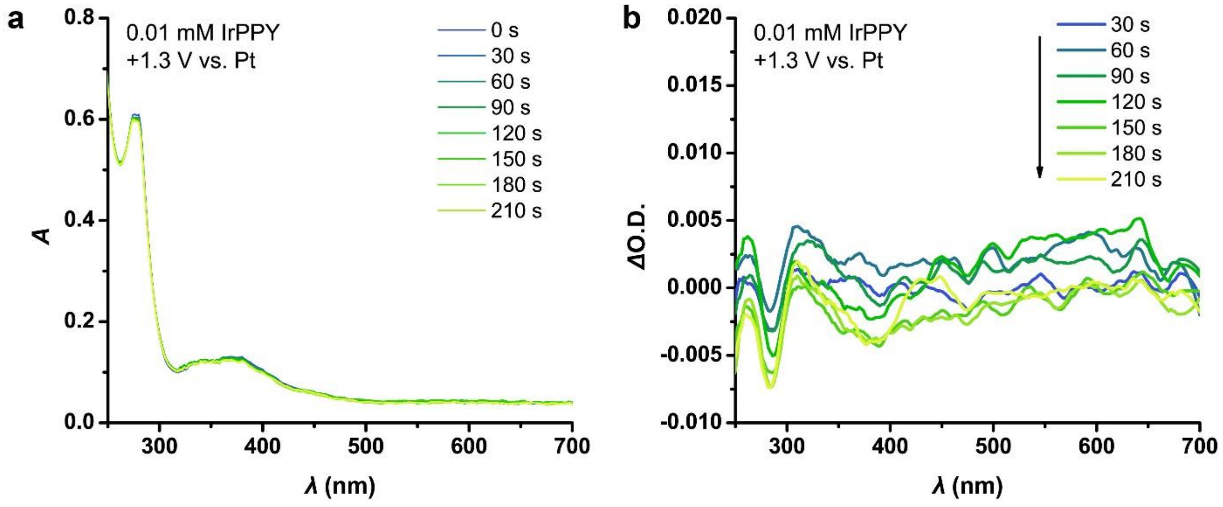

Figure S31. (a) Measured and (b) corresponding differential spectra of UV-vis absorption of 0.01 mM **IrPPY** in the presence of 0.1 M *n*Bu_4NPF_6 upon oxidation under N_2.

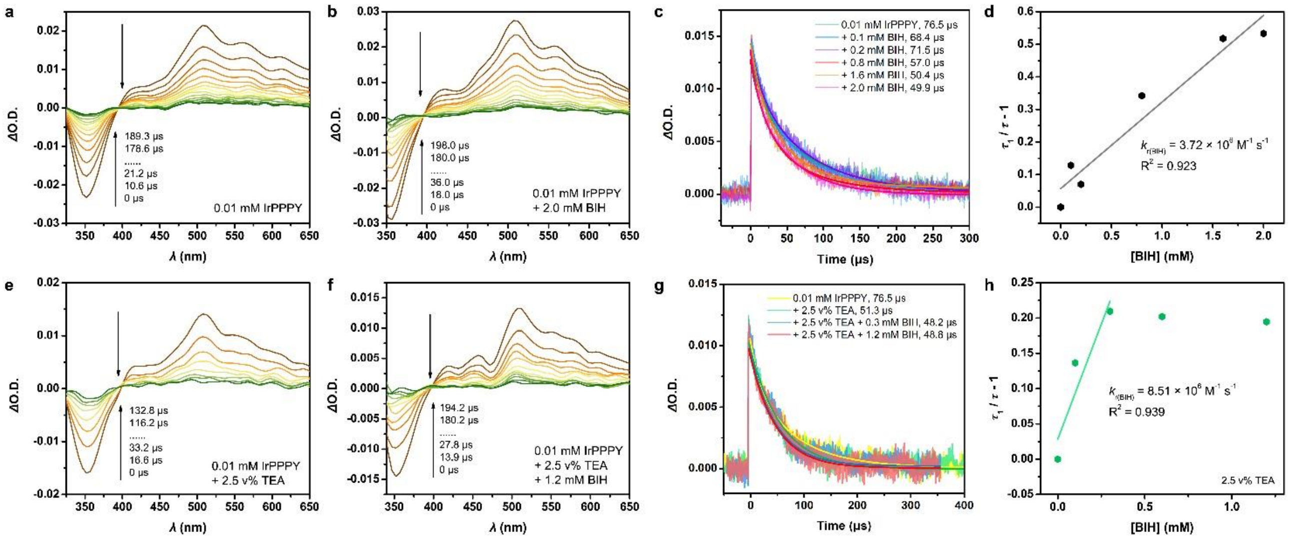

Figure S32. Nanosecond TA spectra of **IrPPPY**. (a) 0.01 mM **IrPPPY**, (b) 0.01 mM **IrPPPY** with 2.0 mM BIH, (c) kinetic traces of **IrPPPY** with 0~2.0 mM BIH followed at 520 nm, (d) Plots of (τ_1 / τ - 1) versus the concentration of BIH with linear fitting. (e) 0.01 mM **IrPPPY** with 2.5 v% TEA. (f) 0.01 mM **IrPPPY** with 2.5 v% TEA and 1.2 mM BIH. (g) kinetic traces of **IrPPPY** with 2.5 v% TEA and 0~1.2 mM BIH followed at 520 nm. (h) Plots of (τ_1 / τ - 1) versus the concentration of BIH in the presence of 2.5 v% TEA with linear fitting. The data were collected in Ar-saturated CH_3CN upon excitation at 450 nm.

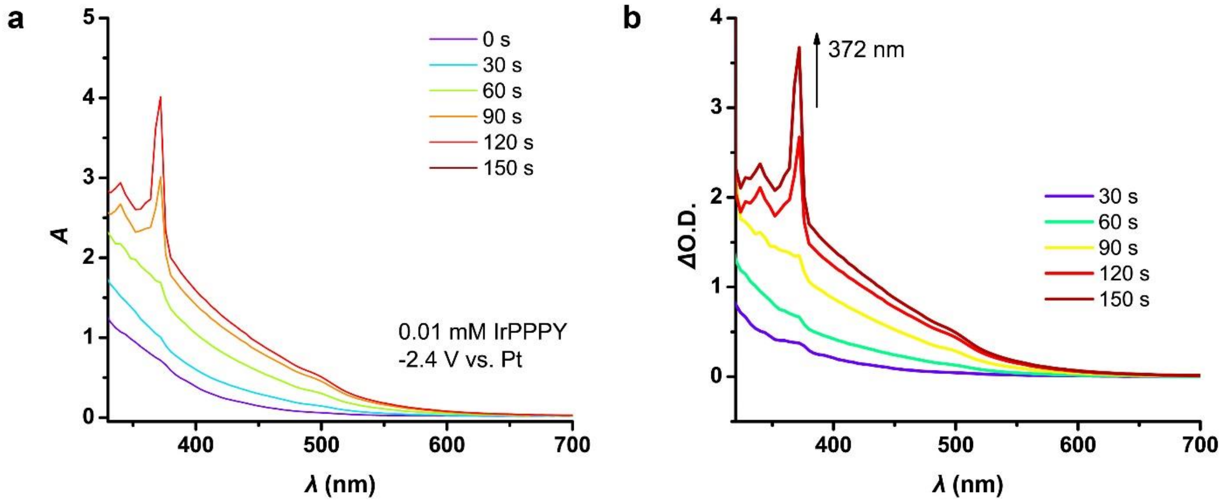

Figure S33. (a) Measured and (b) corresponding differential spectra of UV-vis absorption of 0.01 mM **IrPPPY** in the presence of 0.1 M $n\mathrm{Bu_4NPF_6}$ upon reduction under N_2. The sharp peak should be induced by the long step during the fast scans (5 nm). The solution was changed from pale yellow to brown, which can be recovered by exposing to air.

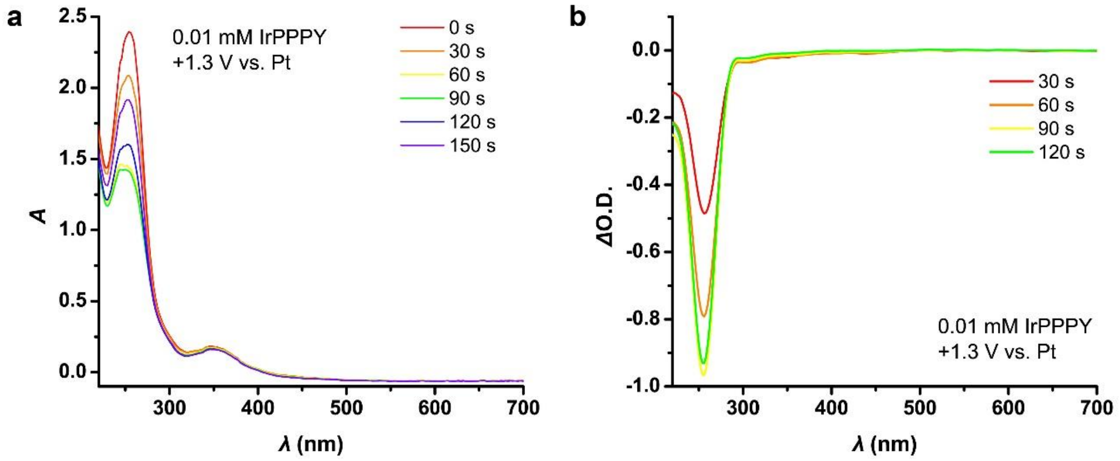

Figure S34. (a) Measured and (b) corresponding differential spectra of UV-vis absorption of 0.01 mM **IrPPPY** in the presence of 0.1 M *n*Bu_4NPF_6 upon oxidation under N_2.

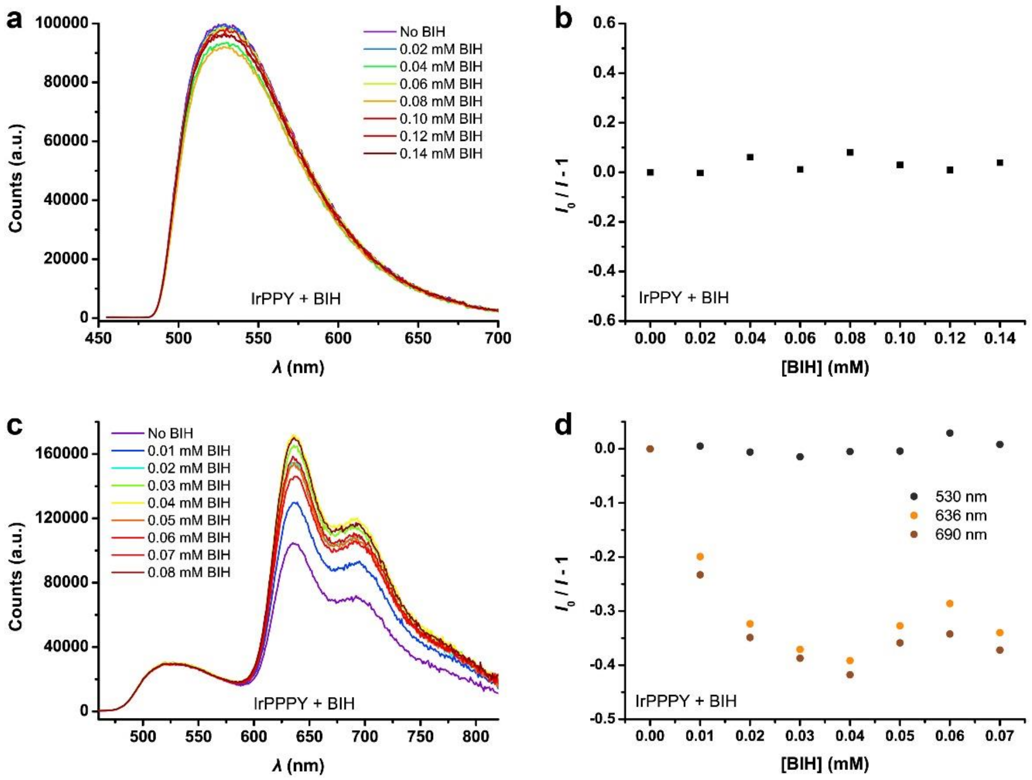

Figure S35. (a) Fluorescence spectra of a CH_3CN solution containing 0.05 mM **IrPPY** in the presence of 0~0.14 mM BIH, respectively. (b) Plot of ratio of fluorescence intensity of **IrPPY** versus [BIH]. (c) Fluorescence spectra of a CH_3CN solution containing 0.05 mM **IrPPPY** in the presence of 0~0.08 mM BIH, respectively. (d) Plot of ratio of fluorescence intensity of **IrPPPY** versus [BIH]. The increased intensity may be caused by the reaction between the remnant air and BIH, as the ^{3}IL of **IrPPPY** is extremely sensitive to air, but this phenomenon still requires further investigations.

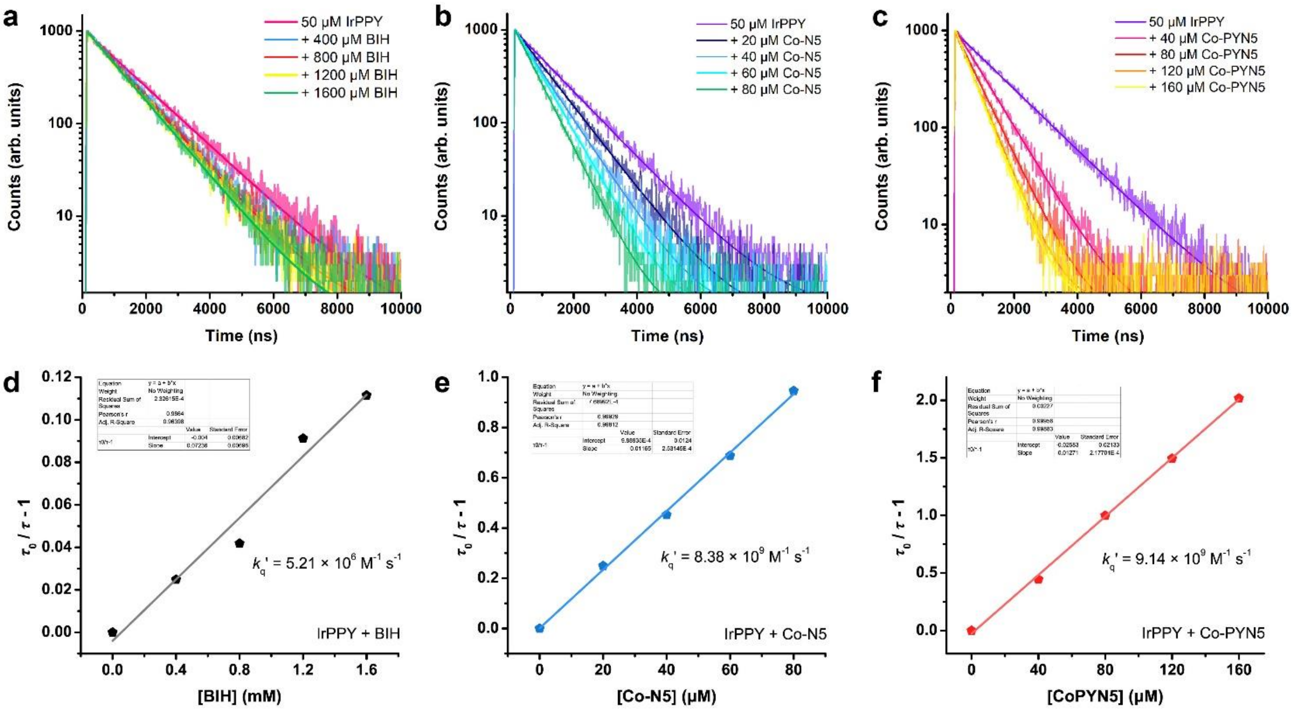

Figure S36. Time-resolved fluorescence decay traces of a CH_3CN solution containing 0.050 mM **IrPPY** in the presence of (a) 0~1.6 mM BIH, (b) 0~80 µM **Co-N5**, or (c) (b) 0~160 µM **Co-PYN5**, respectively, as well as corresponding linear fitting plots of ratio of fluorescence lifetime versus (d) [BIH], (e) [**Co-N5**] or (f) [**Co-PYN5**], respectively. Excitation laser is 445.6 nm.

Supporting Tables

Table S1. Supplemental Redox properties and photophysical properties of Ir PSs.[a]

Complex	λ_{ab} (nm)	Isosbestic point (nm)	$E_{0\text{-}0}$ (V)	λ_{em}[b] (nm) at 298 K	λ_{em}[b] (nm) at 77 K
**IrPPY**	371	492	2.52	519	505 (536)
**IrPPPY**	353	480	2.58	524, 636, 692	512 (544), 630, 690

[a] The values are measured in deaerated, dry CH_3CN at 298 K unless otherwise noted.

[b] The values are measured in deaerated, dry DCM.

Table S2. Simulated excited state properties of the low-lying bright singlet excited states of **IrPPPY** in CH_3CN such as excitation energies (in eV), excitation wave lengths (in nm), oscillator strengths, spin contamination, MO pairs, leading transitions as represented by charge density differences (CDDs; charge transfer takes place from red to blue). All results were obtained in Gaussian 16.

Nr	En eV	λ nm	f	⟨S²⟩	Wgt %	From	To	Character
S_6	3.19	389	0.4199	0.000	35	179	183	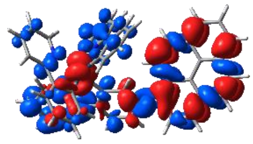
					9	179	184	
					35	181	183	
S_8	3.23	384	0.0894	0.000	10	179	183	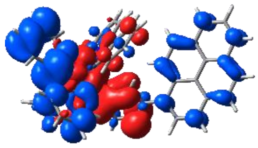
					19	179	184	
					11	179	185	
					28	181	184	
					10	181	185	
					11	182	186	
S_{10}	3.27	379	0.1205	0.000	9	179	185	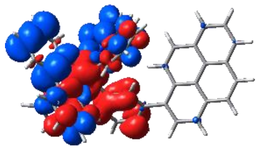
					12	180	184	
					36	180	185	
					9	181	184	
					21	181	185	

Table S3. Simulated excited state properties of the low-lying bright singlet excited states of **IrPPPY** in CH_3CN such as excitation energies (in eV), excitation wave lengths (in nm), oscillator strengths, spin contamination, MO pairs, leading transitions as represented by charge density differences (CDDs; charge transfer takes place from red to blue). All results were obtained in ORCA 5.0.

Nr	En<br>eV	λ<br>nm	f	$\langle S^2 \rangle$	Wgt<br>%	From	To	Character
S_1	2.76	449	0.0578	0.000	84	211	212	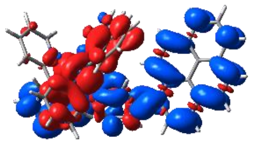
S_4	2.99	415	0.0625	0.000	13<br>19<br>36<br>17	208<br>209<br>210<br>211	212<br>212<br>212<br>214	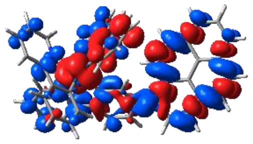
S_6	3.14	395	0.4587	0.000	47<br>32	208<br>210	212<br>212	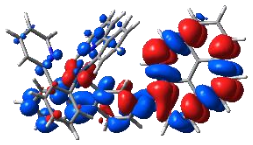
S_8	3.18	391	0.0814	0.000	20<br>11<br>41<br>13	208<br>208<br>210<br>210	213<br>214<br>213<br>214	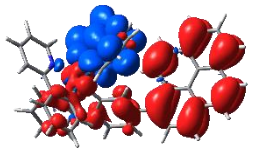
S_9	3.22	385	0.0958	0.000	24<br>20<br>23	209<br>210<br>211	214<br>214<br>215	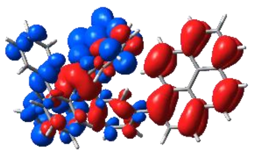

Table S4. Calculated excited state energies, oscillator strengths and composition of the lowest spin-orbit states in **IrPPPY** in CH_3CN based on the spin-free singlet and triplet states, respectively. All result were obtained in ORCA 5.0.

State	Composition	Weight / %	*ΔE* / eV	*λ* / nm	*f*
SO_7	S_1	34	2.59	478	0.0141
	T_3	27			
	T_5	10			
	T_6	15			
SO_{26}	S_3	27	3.02	410	0.0959
	T_6	11			
	T_{10}	8			
SO_{43}	S_6	18	3.18	390	0.1191
	T_7	10			
	T_8	15			
	T_{10}	32			
	T_{11}	11			
SO_{44}	S_6	59	3.18	390	0.3341
	T_{10}	15			

Table S5. Calculated excited energies and oscillator strengths of the lowest singlet-singlet excitations contributing to the spin-orbit states in **IrPPPY** (CH_3CN). The character of the electronic excitation is indicated by charge density differences; charge transfer takes place from red to blue All result were obtained in ORCA 5.0.

Nr	En eV	λ nm	f	⟨S^2⟩	Wgt %	From	To	Character
S_1	2.76	499	0.0578	0.000	84	211	212	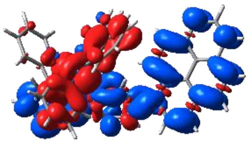
					7	211	215	
S_3	2.95	421	0.0404	0.000	11	208	212	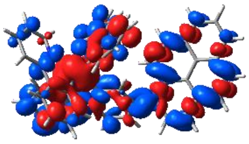
					39	209	212	
					16	210	212	
					19	211	213	
S_6	3.14	395	0.4587	0.000	47	208	212	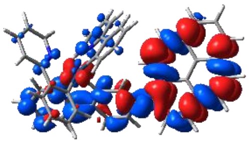
					8	208	213	
					32	210	212	

Table S6. Calculated excited energies of the lowest singlet-triplet excitations contributing to the spin-orbit states in **IrPPPY** (CH_3CN). The character of the electronic excitation is indicated by charge density differences; charge transfer takes place from red to blue All result were obtained in ORCA 5.0.

Nr	En eV	λ nm	f	⟨S²⟩	Wgt %	From	To	Character
T_3	2.66	465	0.000	2.000	12<br>9<br>46<br>9	209<br>210<br>211<br>211	214<br>213<br>213<br>215	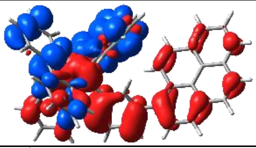
T_5	2.82	440	0.000	2.000	10<br>45<br>10<br>17	208<br>210<br>210<br>211	212<br>212<br>215<br>212	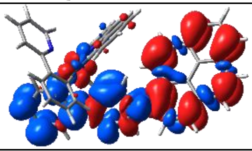
T_6	2.88	431	0.000	2.000	59<br>11<br>11	209<br>209<br>211	212<br>215<br>212	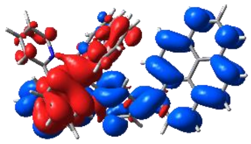
T_7	2.96	419	0.000	2.000	26<br>47<br>9	209<br>209<br>211	213<br>214<br>214	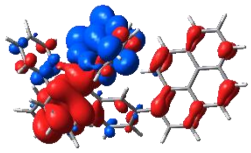
T_8	3.00	414	0.000	2.000	20<br>11<br>11<br>19<br>9	208<br>208<br>209<br>210<br>210	213<br>214<br>213<br>213<br>214	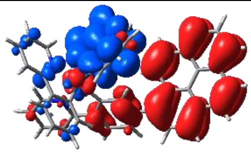
T_{10}	3.08	402	0.000	2.000	16<br>25<br>14<br>14	209<br>209<br>210<br>211	213<br>214<br>213<br>214	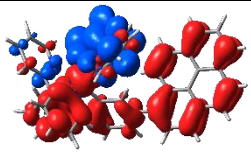
T_{11}	3.11	399	0.000	2.000	8<br>22<br>26<br>12	208<br>208<br>210<br>211	213<br>214<br>214<br>213	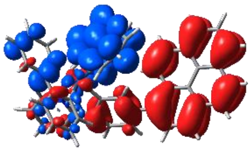

Table S7. Spin-orbit couplings in cm^{-1} for **IrPPPY** in CH_3CN. All result were obtained in ORCA 5.0.

	T_3	T_5	T_6	T_7	T_8	T_{10}	T_{11}
S_1	149	938	882	130	77	48	14
S_3	58	729	482	295	492	407	261
S_6	289	258	296	154	80	195	169

Table S8. Chemical shifts of **IrPPPY** in the ^{1}H NMR titration with **Co-PYN5**.[a]

[**IrPPPY**] (mM)	[**Co-PYN5**] (mM)	Proton H_{p5} + H_{p7}[b]	Proton H_{p6}	Proton H_{p4}	Proton H_{p8} + H_{p9}[b]	Proton H_{p1}	Proton H_{p2}
	0	6.669	6.844	7.22	7.724	8.039	8.118
	0.25	6.662	6.832	7.211	7.721	8.035	8.113
	0.5	6.648	6.818	7.199	7.717	8.0295	8.107
	0.75	6.6355	6.804	7.184	7.7145	8.0235	8.101
1.00	1.00	6.615	6.782	7.164	7.71	8.014	8.091
	1.25	6.6	6.77	7.1535	7.706	8.009	8.085
	1.50	6.591	6.759	7.1455	7.7045	8.004	8.081
	1.75	6.59	6.756	7.144	7.704	8.003	8.079
	2.00	6.572	6.753	7.139	7.6985	7.996	8.073

[a] The proton signals chosen for the fitting are the pyrenyl protons defined in Figure S5, which will participate in the π-π interaction with **Co-PYN5**. It should be noted that the H_{p3} is relatively indiscernible with 1.0 mM **IrPPPY** and the related discussion is made in Figure S8.

[b] The proton signals are merged due to their close proximity and the peak broadening caused by the paramagnetic Co(II) species.

Table S9. Binding fit results for **IrPPPY/Co-PYN5**.

Host:Guest Model	Flavor	Applicable? Y/N	*K* (M^{-1})	*K* error (%)	Total root mean square	Total covariance	Optimal model*
1:1	None	Y	K_{11} = 199	±3.5	0.00373	0.0166	√
1:2	None	N			N.A.		
1:2	Non-cooperative	Y	K_{11} = K_{12} = 18172	±20.1	0.00306	0.0112	
2:1	None	Y	K_{11} = 4804<br>K_{21} = -487	±6.2<br>±2.1	0.00185	0.00414	
2:1	Non-cooperative	Y	K_{11} = K_{21} = 6251	±16.5	0.00214	0.00556	

*It can be seen that four models can be established with approaching values of total root mean square and total covariance. However, the *K* errors of 1:2 non-cooperative and 2:1 non-cooperative models are relatively large (> 10%). Then, it can be seen that the total root mean square and total covariance values of 2:1 model are smaller than those of 1:1 model, but the minus value of K_{21} indicate that the 2:1 model is not applicable. To sum up, 1:1 model is v should be the optimal model to afford the binding constant K_{11} = 199±7 M^{-1}. The error bar was calculated according to the corresponding *K* error value.

Table S10. Chemical shifts of **IrPPPY** in the ^{1}H NMR titration with **Co-N5**.[a]

[**IrPPPY**] (mM)	[**Co-N5**] (mM)	Proton H_{p5} + H_{p7}[b]	Proton H_{p6}	Proton H_{p4}	Proton H_{p8} + H_{p9}[b]	Proton H_{p1}	Proton H_{p2}
	0	6.669	6.844	7.22	7.724	8.039	8.118
	0.25	6.659	6.838	7.216	7.72	8.034	8.112
	0.5	6.655	6.836	7.213	7.718	8.031	8.108
	0.75	6.646	6.827	7.205	7.715	8.025	8.104
1.00	1.00	6.631	6.814	7.199	7.711	8.023	8.095
	1.25	6.626	6.812	7.192	7.708	8.019	8.093
	1.50	6.616	6.802	7.184	7.7085	8.015	8.089
	1.75	6.609	6.795	7.178	7.7065	8.012	8.085
	2.00	6.608	6.792	7.177	7.7065	8.012	8.086

[a] The proton signals chosen for the fitting are the pyrenyl protons defined in Figure S5, which will participate in the possible *π*-*π* interaction with **Co-N5**. It should be noted that the H_{p3} is relatively indiscernible with 1.0 mM **IrPPPY** and the related discussion is made in Figure S8.

[b] The proton signals are merged due to their close proximity and the peak broadening caused by the paramagnetic Co(II) species.

Table S11. Binding fit results for **IrPPPY/Co-N5**.

Host:Guest Model	Flavor	Applicable? Y/N	K (M^{-1})	K error (%)	Total root mean square	Total covariance	Optimal model*
1:1	None	Y	K_{11} = 60	±2.9	0.00460	0.0327	√
1:2	None	Y	K_{11} = 87587<br>K_{12} = 4752	±145.9<br>±20.7	0.00154	0.00886	
1:2	Non-cooperative	Y	K_{11} = K_{12} = 25511	±18.1	0.00156	0.00906	
2:1	None	N			N.A.		
2:1	Non-cooperative	Y	K_{11} = K_{21} = 1813	±11.2	0.00151	0.00851	

*It can be seen that four models can be established with approaching values of total root mean square and total covariance. However, the K errors of 1:2, 1:2 non-cooperative and 2:1 non-cooperative models are relatively large (> 10%), while that of the 1:1 model is smaller than 10%. Despite its higher total root mean square and total covariance, while considering that the binding affinity between **Co-N5** and **IrPPPY** should be weaker than that of **IrPPPY/Co-PYN5**, 1:1 model should be the optimal model to afford a small binding constant K_{11} = 60±2 M^{-1}. The error bar was calculated according to the corresponding K error value.

Table S12. Chemical shifts of proton signals of **IrPPY** in the ^{1}H NMR titration with **Co-PYN5**.[a]

Host [**IrPPY**] (mM)	Guest [**Co-PYN5**] (mM)	Proton H_d	Proton H_f	Proton H_c	Proton H_e
1.00	0	7.55	6.889	6.869	6.857
	0.25	7.543	6.885	6.866	6.836
	0.5	7.535	6.866	6.83	6.81
	0.75	7.527	6.856	6.816	6.7945
	1.00	7.521	6.853	6.8225	6.781
	1.25	7.518	6.852	6.8255	6.7715
	1.50	7.514	6.851	6.818	6.7695
	1.75	7.512	6.8495	6.8155	6.7715
	2.00	7.51	6.8475	6.805	6.7715

[a] The proton signals chosen for the fitting are H_c, H_d, H_e and H_f defined in Figure S2, which will participate in the CH-π interaction with **Co-PYN5**.

Table S13. Binding fit results for **IrPPY/Co-PYN5**.

Host:Guest Model	Flavor	Applicable? Y/N	*K* (M^{-1})	*K* error (%)	Total root mean square	Total covariance	Optimal model*
1:1	None	Y	K_{11} = 32303	±65.1	0.00460	0.0327	
1:2	None	Y	K_{11} = 18404<br>K_{12} = 383	±57.5<br>±90.3	0.00438	0.0295	
1:2	Non-cooperative	Y	K_{11} = K_{12} = 12975	±82.6	0.00453	0.0316	
2:1	None	N	N.A.				
2:1	Non-cooperative	Y	K_{11} = K_{21} = 115	±0.9	0.00460	0.0326	√

*It can be seen that four models can be established with approaching values of total root mean square and total covariance. However, the *K* errors of 1:1, 1:2 and 1:2 non-cooperative models are extremely large (>> 10%), while that of the 2:1 non-cooperative model is very small, which should be the optimal model to afford the binding constant K_{11} = 115±1 M^{-1}. The error bar was calculated according to the corresponding *K* error value.

Table S14. Binding free energy results obtained by DFT calculations.

Reaction	Δ*G* (kcal mol^{-1})
**IrPPPY** + **Co-PYN5** → **IrPPPY/Co-PYN5**	-3.0
**IrPPY** + **Co-PYN5** → **IrPPY/Co-PYN5**	-0.9
**IrPPPY** + **Co-N5** → **IrPPPY/Co-N5**	-2.2
2 **Co-PYN5** → **Co-PYN5/Co-PYN5**	-0.3
2 **IrPPPY** → **IrPPPY/IrPPPY**	-3.3
(**Co-PYN5/Co-PYN5**) + (**IrPPPY/IrPPPY**) → 2(**IrPPPY/Co-PYN5**)	-2.4

Table S15. Photocatalytic CO_2 reduction to CO by **IrPPPY**/**Co-PYN5** for Φ determination.*

Entry	Wavelength (nm)	Time (min)	n(CO) (μmol)	n(H_2) (μmol)	Φ(CO) (%)	CO%
1	450	30	19.0±0.6	1.43±0.07	3.3±0.2	93
2	425	30	32.6±2.6	2.46±0.04	5.7±0.4	93
3	405	30	26.7±1.2	2.67±0.09	4.6±0.2	91
4	425	10	16.4±0.9	0.82±0.04	8.6±0.8	95
5#	425	10	10.9±1.1	0.32±0.05	14.3±1.0	98

*Standard condition: **Co-PYN5** (0.1 mM), **IrPPPY** (0.1 mM), TFE (4.0 v%), TEA (2.5 v%), and BIH (25 mM) in 4.0 mL CH_3CN with LED irradiation at 100 mW cm^{-2} under 1 atm CO_2.

#Light intensity = 50 mW cm^{-2}.

Table S16. Φ_{em} data of **IrPPPY**.

Entry	Excitation λ (nm)	Φ_{em} (%)
1	450	2.11
2	425	2.50
3	405	1.02

Table S17. Photocatalytic CO_2 reduction to CO by **IrPPPY**/**Co-PYN5** in the absence of certain component.*

Entry	Conditions	n(CO) (μmol)	n(H_2) (μmol)
1	No Co catalyst	N.D.	2.20±0.40
2	No Ir PS	N.D.	N.D.
3	Under N_2 instead of CO_2	N.D.	N.D.
4	No irradiation	N.D.	N.D.
5	No BIH	8.60±0.60	1.20±0.10

*Standard condition: **Co-PYN5** (0.1 mM), **IrPPPY** (0.1 mM), TEA (2.5 v%), TFE (4.0 v%), and BIH (25 mM) in 4.0 mL CH_3CN within 4 h of 450 nm irradiation under 1 atm CO_2.

Table S18. Photocatalytic CO_2 reduction to CO by **IrPPPY/Co-PYN5** with varied volume ratios (v%) of different proton sources.*

Entry	Proton source	Proton source (v%)	n(CO) (μmol)	n(H_2) (μmol)	CO%
1	No proton source	0	16.4±1.0	1.29±0.02	93
2	H_2O	2.0	36.6±2.3	4.35±0.41	89
3	PhOH	2.0	33.3±1.5	5.23±0.12	86
4	TFE	2.0	49.4±2.7	2.61±0.06	95
5	TFE	4.0	67.0±1.1	4.47±0.07	94
6	TFE	6.0	58.3±1.2	8.45±0.22	87
7	TFE	8.0	62.3±0.9	7.68±0.39	89

*Standard condition: **Co-PYN5** (0.1 mM), **IrPPPY** (0.1 mM), TEA (2.5 v%) and BIH (25 mM) in 4.0 mL CH_3CN within 4 h of 450 nm irradiation under 1 atm CO_2.

Table S19. Crystallographic data of **IrPPPY** and **Co-PYN5**·$2CH_3CN$.

Complex	**IrPPPY**	**Co-PYN5**·$2CH_3CN$
Formula	$C_{49}H_{32}IrN_3$	$CoC_{39}H_{43}Cl_2N_9O_8$
CCDC number	2086643	2011002
Formula weight	854.98	2173.18
Crystal system	monoclinic	triclinic
Space group	$P2_1/c$	P-1
Z	4	2
a / Å	22.0367(5)	9.5930(3)
b / Å	9.04478(16)	11.4712(3)
c / Å	17.4133(4)	20.8153(5)
α / °	90.00	97.797(2)
β / °	94.134(2)	93.627(2)
γ / °	90.00	112.764(2)
V / Å^3	3461.74(13)	2075.77(10)
ρ_{calcd} / g m^{-3}	1.640	3.477
μ / mm^{-1}	7.782	48.182
Crystal size / mm	0.15 × 0.10 × 0.10	0.20 × 0.10 × 0.10
2Θ range for data collection / °	8.04 / 130	8.488 / 147.334
Reflns collected / Indep.	67709 / 5702	33168 / 8228
R_{int}	0.0706	0.0460
Parameters refined	479	8228
F(000)	1696.0	2020.0
Goodness-of-fit on F^2	1.095	1.068
Final R indices [I > 2sigma(I)]	$R_1^a = 0.0322$<br>$wR_2^b = 0.0798$	$R_1^a = 0.0468$<br>$wR_2^b = 0.1287$
R indices (all data)	$R_1 = 0.0324$<br>$wR_2 = 0.0804$	$R_1 = 0.0503$<br>$wR_2 = 0.1319$
Largest diff. peak and hole / e Å^{-3}	1.07 / -1.36	0.68 / -0.57

$^aR_1 = \sum||F_0| - |F_c||/\sum F_0$. $^bwR_2 = \{[\sum(F_0^2-F_c^2)/\sum w\,(F_0^2)^2]\}^{1/2}$, $w = 1/[s^2(F_o^2) + (0.2000P)^2 + 0.0000P]$; where $P = (F_o^2 + 2F_c^2)/3$

SI References:

1. Sheldrick, G. M., A short history of SHELX. *Acta Crystallogr., Sect. A: Found. Crystallogr.* **2008,** *64* (Pt 1), 112-122.
2. Bindfit. http://app.supramolecular.org/bindfit/.
3. Thordarson, P., Determining association constants from titration experiments in supramolecular chemistry. *Chem. Soc. Rev.* **2011,** *40* (3), 1305-1323.
4. Guo, Z.; Chen, G.; Cometto, C.; Ma, B.; Zhao, H.; Groizard, T.; Chen, L.; Fan, H.; Man, W.-L.; Yiu, S.-M.; Lau, K.-C.; Lau, T.-C.; Robert, M., Selectivity control of CO versus $HCOO^{-}$ production in the visible-light-driven catalytic reduction of CO_2 with two cooperative metal sites. *Nat. Catal.* **2019,** *2* (9), 801-808.
5. Kuhn, H.; Braslavsky, S.; Schmidt, R., Chemical actinometry (IUPAC technical report). *Pure Appl. Chem.* **2004,** *76* (12), 2105-2146.
6. Thoi, V. S.; Kornienko, N.; Margarit, C. G.; Yang, P.; Chang, C. J., Visible-light photoredox catalysis: selective reduction of carbon dioxide to carbon monoxide by a nickel N-heterocyclic carbene-isoquinoline complex. *J. Am. Chem. Soc.* **2013,** *135* (38), 14413-14424.
7. Wang, P.; Guo, S.; Wang, H. J.; Chen, K. K.; Zhang, N.; Zhang, Z. M.; Lu, T. B., A broadband and strong visible-light-absorbing photosensitizer boosts hydrogen evolution. *Nat. Commun.* **2019,** *10* (1), 3155.
8. Bhattacharyya, A.; Mukherjee, S.; Chadha, A.; Prasad, E., Diffusion of Solvent-Separated Ion Pairs Controls Back Electron Transfer Rate in Graphene Quantum Dots. *J. Phys. Chem. C* **2018,** *122* (28), 15819-15825.
9. Frisch, M. J.; Trucks, G. W.; Schlegel, H. B.; Scuseria, G. E.; Robb, M. A.; Cheeseman, J. R.; Scalmani, G.; Barone, V.; Mennucci, B.; Petersson, G. A.; Nakatsuji, H.; Caricato, M.; Li, X.; Hratchian, H. P.; Izmaylov, A. F.; Bloino, J.; Zheng, G.; Sonnenberg, J. L.; Hada, M.; Ehara, M.; Toyota, K.; Fukuda, R.; Hasegawa, J.; Ishida, M.; Nakajima, T.; Honda, Y.; Kitao, O.; Nakai, H.; Vreven, T.; Montgomery, J. A., Jr.; Peralta, J. E.; Ogliaro, F.; Bearpark, M.; Heyd, J. J.; Brothers, E.; Kudin, K. N.; Staroverov, V. N.; Kobayashi, R.; Normand, J.; Raghavachari, K.; Rendell, A.; Burant, J. C.; Iyengar, S. S.; Tomasi, J.; Cossi, M.; Rega, N.; Millam, J. M.; Klene, M.; Knox, J. E.; Cross, J. B.; Bakken, V.; Adamo, C.; Jaramillo, J.; Gomperts, R.; Stratmann, R. E.; Yazyev, O. A., A. J.; Cammi, R. P., C.; Ochterski, J. W.; Martin, R. L.; Morokuma, K.; Zakrzewski, V. G.; Voth, G. A.; Salvador, P.; Dannenberg, J. J.; Dapprich, S.; Daniels, A. D.; Farkas, Ö.; Foresman, J. B.; Ortiz, J. V.; Cioslowski, J.; Fox, D. J. *Gaussian 09*, Revision C.01; Gaussian, Inc.: Wallingford CT, 2009.
10. Perdew, J. P.; Burke, K.; Ernzerhof, M., Generalized Gradient Approximation Made Simple. *Phys. Rev. Lett.* **1996,** *77* (18), 3865-3868.
11. Grimme, S.; Antony, J.; Ehrlich, S.; Krieg, H., A consistent and accurate ab initio parametrization of density functional dispersion correction (DFT-D) for the 94 elements H-Pu. *J Chem Phys* **2010,** *132* (15), 154104.
12. Goerigk, L.; Grimme, S., Efficient and Accurate Double-Hybrid-Meta-GGA Density Functionals-Evaluation with the Extended GMTKN30 Database for General Main Group Thermochemistry, Kinetics, and Noncovalent Interactions. *J. Chem. Theory Comput.* **2011,** *7* (2), 291-309.
13. Chiodo, S.; Russo, N.; Sicilia, E., LANL2DZ basis sets recontracted in the framework of density functional theory. *J Chem Phys* **2006,** *125* (10), 104107.
14. Marenich, A. V.; Cramer, C. J.; Truhlar, D. G., Performance of SM6, SM8, and SMD on the SAMPL1 test set for the prediction of small-molecule solvation free energies. *J. Phys. Chem. B* **2009,** *113* (14), 4538-43.
15. Castillo, U. J.; Torres, A. E.; Fomine, S., Zinc-, cadmium-, and mercury-containing one-dimensional tetraphenylporphyrin arrays: a DFT study. *J. Mol. Model.* **2014,** *20* (4), 2206.
16. Adamo, C.; Barone, V., Toward reliable density functional methods without adjustable parameters: The PBE0 model. *J. Chem. Phys.* **1999,** *110* (13), 6158-6170.
17. Grimme, S.; Ehrlich, S.; Goerigk, L., Effect of the damping function in dispersion corrected density functional

theory. *J. Comput. Chem.* **2011,** *32* (7), 1456-1465.
18. Lu, T.; Chen, F., Multiwfn: a multifunctional wavefunction analyzer. *J. Comput. Chem.* **2012,** *33* (5), 580-92.
19. Humphrey, W.; Dalke, A.; Schulten, K., VMD: Visual molecular dynamics. *J. Mol. Graphics* **1996,** *14* (1), 33-38.
20. C. Y. Legault, *CYLview, 1.0b* **2009,** *Université de Sherbrooke*, http://www.cylview.org.
21. Frisch, M.; Trucks, G.; Schlegel, H.; Scuseria, G.; Robb, M.; Cheeseman, J.; Scalmani, G.; Barone, V.; Petersson, G.; Nakatsuji, H., Gaussian 16 Rev. B. 01, Wallingford, CT. **2016**.
22. Beck, A. D., Density-functional thermochemistry. III. The role of exact exchange. *J. Chem. Phys* **1993,** *98* (7), 5648-6.
23. Weigend, F., Accurate Coulomb-fitting basis sets for H to Rn. *Phys. Chem. Chem. Phys.* **2006,** *8* (9), 1057-1065.
24. Weigend, F.; Ahlrichs, R., Balanced basis sets of split valence, triple zeta valence and quadruple zeta valence quality for H to Rn: Design and assessment of accuracy. *Phys. Chem. Chem. Phys.* **2005,** *7* (18), 3297-3305.
25. Marenich, A. V.; Cramer, C. J.; Truhlar, D. G., Universal solvation model based on solute electron density and on a continuum model of the solvent defined by the bulk dielectric constant and atomic surface tensions. *J. Phys. Chem. B* **2009,** *113* (18), 6378-6396.
26. Mennucci, B.; Cappelli, C.; Guido, C. A.; Cammi, R.; Tomasi, J., Structures and properties of electronically excited chromophores in solution from the polarizable continuum model coupled to the time-dependent density functional theory. *J. Phys. Chem. A* **2009,** *113* (13), 3009-3020.
27. Hay, P. J., Theoretical studies of the ground and excited electronic states in cyclometalated phenylpyridine Ir(III) complexes using density functional theory. *J. Phys. Chem. A* **2002,** *106* (8), 1634-1641.
28. Hung, J.-Y.; Lin, C.-H.; Chi, Y.; Chung, M.-W.; Chen, Y.-J.; Lee, G.-H.; Chou, P.-T.; Chen, C.-C.; Wu, C.-C., Phosphorescent Ir(III) complexes bearing double benzyldiphenylphosphine cyclometalates; strategic synthesis, fundamental and integration for white OLED fabrication. *J. Mater. Chem.* **2010,** *20* (36), 7682-7693.
29. Liao, J.-L.; Chi, Y.; Sie, Z.-T.; Ku, C.-H.; Chang, C.-H.; Fox, M. A.; Low, P. J.; Tseng, M.-R.; Lee, G.-H., Ir(III)-Based phosphors with bipyrazolate ancillaries; rational design, photophysics, and applications in organic light-emitting diodes. *Inorg. Chem.* **2015,** *54* (22), 10811-10821.
30. Neese, F.; Wennmohs, F.; Becker, U.; Riplinger, C., The ORCA quantum chemistry program package. *J. Chem. Phys.* **2020,** *152* (22), 224108.
31. van Lenthe, E.; Baerends, E.-J.; Snijders, J. G., Relativistic total energy using regular approximations. *J. Chem. Phys.* **1994,** *101* (11), 9783-9792.
32. Kovrizhina, A. R.; Samorodova, E. I.; Khlebnikov, A. I., 11H-Indeno [1, 2-b] quinoxalin-11-one 2-(4-ethylbenzylidene) hydrazone. *Molbank* **2021,** *2021* (4), M1299.
33. Debefve, L. M.; Pollock, C. J., Systematic assessment of DFT methods for geometry optimization of mononuclear platinum-containing complexes. *Phys. Chem. Chem. Phys.* **2021,** *23* (43), 24780-24788.
34. Pantazis, D. A.; Neese, F., All-electron scalar relativistic basis sets for the 6 p elements. *Theor. Chem. Acc.* **2012,** *131* (11), 1-7.
35. Takano, Y.; Houk, K., Benchmarking the conductor-like polarizable continuum model (CPCM) for aqueous solvation free energies of neutral and ionic organic molecules. *J. Chem. Theory Comput.* **2005,** *1* (1), 70-77.
36. Steinmetzer, J.; Kupfer, S.; Gräfe, S., pysisyphus: Exploring potential energy surfaces in ground and excited states. *Int. J. Quantum Chem.* **2021,** *121* (3), e26390.
37. Plasser, F.; Ruckenbauer, M.; Mai, S.; Oppel, M.; Marquetand, P.; González, L., Efficient and flexible computation of many-electron wave function overlaps. *J. Chem. Theory Comput.* **2016,** *12* (3), 1207-1219.

Appendix: Cartesian coordinates (xyz) for all optimized structures

IrPPPY/Co-PYN5

2 4

Co	10.52143500	-0.89411400	0.27991000
N	8.56184700	-0.23542500	-0.40044300
N	10.56791900	-0.63265000	-1.96484200
N	9.13524900	-0.73271400	2.06152200
N	11.67734900	-1.57958300	2.12041000
H	11.41468300	-2.57200000	2.16824200
N	12.66025400	-1.26229900	-0.41994800
H	13.03329900	-0.30506500	-0.44624800
C	9.51726800	-0.20944900	-2.58392500
C	8.33901000	0.00788000	-1.70743200
C	4.48080700	2.92214200	-0.12806600
H	5.53021000	2.81712800	0.15162600
C	7.55435000	-0.06980100	0.48010800
C	7.09278100	0.43449800	-2.18152900
H	6.94568900	0.63327200	-3.24323300
C	6.03254200	0.61979300	-1.27793400
C	3.93125800	2.03971300	-1.12147500
C	4.68378800	1.00429100	-1.74526400
C	6.28256500	0.35278400	0.08031100
H	5.48095000	0.44536700	0.81247600
C	7.90587500	-0.37969700	1.88850100
C	1.99208700	1.43130500	-2.55791600
C	9.39450200	0.07957500	-4.04767500
H	10.33083700	-0.08766600	-4.58979200
H	9.08850800	1.12814500	-4.19415500
H	8.60666700	-0.54805400	-4.49485300
C	2.56983000	2.24313200	-1.52793200
C	1.77755700	3.25286600	-0.90132000
C	0.40444000	3.42557000	-1.27510100
C	3.72590000	3.90625600	0.45355100
H	4.16893500	4.57003300	1.20071000
C	2.34862100	4.09650900	0.10445200
C	2.79503100	0.46563200	-3.19556600
H	2.36053000	-0.14838400	-3.98794900
C	4.11338200	0.26504700	-2.79843600
H	4.70658000	-0.52106700	-3.27133400
C	11.82346400	-0.89615200	-2.65726900
H	12.34961200	0.06583800	-2.80068100
H	11.67570100	-1.34797700	-3.65032600
C	9.67131900	-1.08292300	3.37024800
H	9.43237100	-2.14448500	3.56790400
H	9.23777600	-0.48499700	4.18641900
C	6.84414000	-0.27577000	2.93936700
H	7.21859200	-0.51055100	3.94105700
H	6.02104300	-0.97065700	2.70474200
H	6.41321700	0.73832500	2.94847500
C	0.61292200	1.62036700	-2.90635300
H	0.18225100	0.97612500	-3.67709600
C	-0.15143400	2.57181500	-2.28657400
H	-1.20434600	2.69929500	-2.55051000
C	12.66200300	-1.81191600	-1.78062000
H	12.21511400	-2.81705400	-1.73805200
H	13.68345600	-1.90455700	-2.19141500
C	11.17987700	-0.90093300	3.32261500
H	11.43475000	0.16691600	3.23790900
H	11.64262100	-1.29563000	4.24498900
C	1.54762100	5.08515900	0.71168600
H	1.98935300	5.72582600	1.47919200
C	-0.35974100	4.42252800	-0.63640900
H	-1.40912000	4.54375100	-0.91650400
C	0.20903900	5.24189400	0.34314700
H	-0.40112300	6.00689900	0.82876900
C	13.44333600	-2.04147900	0.55097600
H	14.52585100	-2.00054300	0.33675200
H	13.12123900	-3.09367400	0.48018800
C	13.13312800	-1.49223600	1.93009300
H	13.68555100	-2.04095400	2.71326900
H	13.42029100	-0.42954700	1.99103100
C	-2.24784500	0.14210700	-0.45800400
H	-3.25615600	0.23960100	-0.86047500
C	-1.83865000	1.01200500	0.61361000
C	-2.70574600	1.97780100	1.20756800
C	0.01134800	1.81181300	2.07762900
C	-0.47705300	0.92335000	1.06433600
C	0.40717900	-0.04859300	0.50163700
C	1.76548700	-0.13681900	0.95342300
C	-1.40300800	-0.79518800	-0.98960300
H	-1.74381900	-1.44192200	-1.80263000
C	-0.05531400	-0.93809600	-0.52177200
C	-0.86545300	2.77326600	2.61534600
H	-0.49706800	3.45778700	3.38364500
C	-2.18720600	2.84884100	2.18636600
H	-2.85599700	3.58602900	2.63695100
C	1.37657600	1.70531500	2.50670000
H	1.73092800	2.39796600	3.27456100
C	2.21948400	0.76962900	1.96995000
H	3.25839000	0.69879300	2.30291600
C	0.83105100	-1.89386500	-1.05840900

H	0.47245900	-2.56644200	-1.84191700
C	2.61744900	-1.10437200	0.38316000
H	3.65427200	-1.16004200	0.72486600
C	2.15138800	-1.97370700	-0.60700700
H	2.82890300	-2.71353900	-1.03994000
C	-10.16941100	0.99579700	-0.64909000
H	-10.57312100	-0.01711700	-0.69577600
C	-10.96018300	2.10852900	-0.92081800
H	-12.00997000	1.97571300	-1.18519800
C	-10.37415300	3.37915100	-0.84997000
H	-10.96188900	4.27507000	-1.06028500
C	-9.02801800	3.48809300	-0.50944900
H	-8.55265900	4.46793900	-0.45035900
C	-8.27468600	2.33230000	-0.23898500
C	-6.86084700	2.29955600	0.13800900
C	-6.30724800	1.00232400	0.37913700
C	-4.95318400	0.94617700	0.75612100
H	-4.51279800	-0.02546200	0.98696200
C	-4.13709800	2.09007800	0.84077100
C	-4.71488800	3.35677600	0.59207000
H	-4.09738600	4.25630000	0.64824300
C	-6.06269800	3.45576900	0.25438500
H	-6.48918300	4.44406900	0.06243400
C	-8.87619400	0.03670300	2.85247300
H	-9.46908300	0.72602800	2.24882200
C	-9.08045700	-0.09082500	4.22302800
H	-9.84867800	0.50761800	4.71422100
C	-8.28204700	-0.99398700	4.93713400
H	-8.41443700	-1.12300600	6.01331400
C	-7.31293900	-1.72763600	4.25725000
H	-6.68098700	-2.43461800	4.79612400
C	-7.14569900	-1.55994300	2.87146400
C	-6.16579200	-2.25996700	2.03597800
C	-5.24515500	-2.60433400	-0.17642100
H	-5.22834100	-2.39402000	-1.24895200
C	-4.31121200	-3.50212200	0.35234100
H	-3.58401600	-3.98096000	-0.31040500
C	-4.29594400	-3.78768300	1.72752200
H	-3.56463400	-4.48595300	2.14106300
C	-5.22616700	-3.16920000	2.56332400
H	-5.21314300	-3.39047600	3.63382500
C	-9.79730300	-2.71982500	0.33465300
H	-9.75067200	-2.42601500	1.38456000
C	-10.69036100	-3.69031600	-0.10945200
H	-11.36347500	-4.17260400	0.60038300
C	-10.69474400	-4.02252900	-1.47045300
H	-11.38214900	-4.77791900	-1.85667000
C	-9.80738200	-3.37816100	-2.32907800
H	-9.79376100	-3.62465300	-3.39123700
C	-8.92525600	-2.40449200	-1.82946200
C	-7.94276900	-1.65398100	-2.61695500
C	-7.75993800	-1.85689100	-4.00018900
H	-8.36702500	-2.59197300	-4.53563300
C	-6.79928600	-1.12618000	-4.70014000
H	-6.65937900	-1.28738100	-5.77151600
C	-6.01668600	-0.18446300	-4.01195300
H	-5.25945100	0.39195400	-4.55203400
C	-6.19561400	0.02056900	-2.63927600
H	-5.56810300	0.75582300	-2.12834900
C	-7.15542800	-0.70091800	-1.89439500
Ir	-7.54629600	-0.55740800	0.08116000
N	-8.86556900	1.09622200	-0.31470700
N	-8.94183300	-2.08601200	-0.49571100
C	-6.19228200	-1.94498900	0.63876500
N	-7.94204100	-0.67692100	2.18812500
N	11.09721000	1.10323500	0.57517200
N	9.97940400	-2.90212400	0.00784100
C	11.34620400	2.23423700	0.69202600
C	9.57558500	-3.98401400	-0.13815000
C	11.65420000	3.63720500	0.84093500
H	12.18716100	3.80437000	1.78894300
H	12.28927400	3.97132400	0.00693900
H	10.72404700	4.22486000	0.84255800
C	9.07615500	-5.32635700	-0.32266500
H	9.88119600	-5.97651800	-0.69658600
H	8.70780600	-5.72326100	0.63499100
H	8.25119800	-5.32006700	-1.05090400

IrPPPY/Co-N5

2 4

C	-0.14849900	3.25272400	-0.55409100
H	0.28773600	2.27178500	-0.73594900
C	-0.65026300	3.56087300	0.75959700
C	-0.61008300	2.63938200	1.85167400
C	-1.85806100	5.19027800	2.20977000
C	-1.27671400	4.84262800	0.94647700
C	-1.34134000	5.78118700	-0.13150600
C	-1.96062700	7.06155400	0.05917500
C	-0.21528000	4.14939900	-1.58641800
H	0.17281700	3.87802900	-2.57173700
C	-0.79728700	5.44753800	-1.41389100
C	-1.81732400	4.24788600	3.25576300
H	-2.25805800	4.50630400	4.22209300
C	-1.21226900	3.00893600	3.07266500

H	-1.17461700	2.30535200	3.90755900
C	-2.47001400	6.47813400	2.37399000
H	-2.90135700	6.72321200	3.34823200
C	-2.51764200	7.37854200	1.34403200
H	-2.98538200	8.35722800	1.48010600
C	-0.86756900	6.38715600	-2.46245100
H	-0.44837000	6.12368700	-3.43701500
C	-2.00738400	7.97009300	-1.01827400
H	-2.47730800	8.94514900	-0.86557600
C	-1.46536600	7.63443100	-2.26229000
H	-1.51134000	8.35216700	-3.08475000
Co	-4.08999400	-1.94855500	-0.32091100
N	-5.81581800	-0.68473200	0.10901100
N	-3.52647900	0.19714400	-0.66509900
N	-5.86875600	-3.25982000	0.14326800
N	-3.37573200	-4.10757600	-0.42549700
H	-3.55501200	-4.34262100	-1.40987600
N	-1.83840800	-1.85131600	-0.68643700
H	-1.47954700	-1.57427100	0.23774700
C	-4.35310400	1.13734100	-0.34828500
C	-5.68917000	0.65667700	0.07888400
C	-6.99422600	-1.23905900	0.45395700
C	-6.75475700	1.50586100	0.41283400
H	-6.62721300	2.58781200	0.38428500
C	-7.97725600	0.93709300	0.77429900
C	-8.10620000	-0.45307100	0.79266600
H	-9.05123600	-0.92154500	1.06663100
C	-7.00471400	-2.72478400	0.44168300
C	-4.07772000	2.60870100	-0.37976100
H	-3.03771500	2.83258200	-0.63852100
H	-4.29797700	3.05050700	0.60533600
H	-4.74118700	3.10503000	-1.10702600
C	-2.17278800	0.49828900	-1.11475400
H	-1.59082600	0.86154300	-0.25127700
H	-2.15707700	1.28828400	-1.88181600
C	-5.66201800	-4.69910900	0.08345400
H	-5.86458200	-5.03077800	-0.95180900
H	-6.32600000	-5.26504300	0.75437000
C	-8.28509500	-3.43814500	0.74700600
H	-8.17806300	-4.52727300	0.71545000
H	-9.05566300	-3.14198400	0.01679200
H	-8.65760000	-3.14535000	1.74157600
C	-1.54045300	-0.77734100	-1.64257800
H	-1.97306100	-1.06044200	-2.61448200
H	-0.45510700	-0.62982900	-1.77820100
C	-4.20421200	-4.97065500	0.42395400
H	-4.00388500	-4.70731900	1.47409100
H	-3.97008900	-6.04128700	0.28367500
C	-1.25605200	-3.15611400	-1.03747400
H	-0.16184700	-3.16913600	-0.89936200
H	-1.46687000	-3.34764300	-2.10274400
C	-1.93001700	-4.20916100	-0.17957100
H	-1.53933000	-5.21761300	-0.40535600
H	-1.75064600	-4.00546100	0.88837500
C	3.63012500	-3.71910400	0.47541000
H	4.58586000	-3.62300600	-0.04252300
C	3.14051300	-4.95883400	0.87707100
H	3.72064200	-5.86039600	0.67659300
C	1.90399600	-5.01074600	1.53439000
H	1.48692800	-5.96553800	1.86078700
C	1.21149600	-3.82547300	1.76946100
H	0.24776800	-3.84190200	2.27955300
C	1.75062200	-2.59945500	1.34342700
C	1.12536500	-1.28845400	1.51566900
C	1.83349700	-0.17778100	0.95823900
C	1.25405200	1.09558300	1.11942800
H	1.79894400	1.96644300	0.75176900
C	0.01153400	1.29647800	1.75054700
C	-0.65027800	0.17568700	2.30843500
H	-1.61484700	0.30831100	2.80210700
C	-0.09307200	-1.09586300	2.19880100
H	-0.61815200	-1.94537500	2.64409500
C	2.01415300	-1.89994000	-2.37433600
H	2.10511200	-2.79676400	-1.75942100
C	1.36706100	-1.92826400	-3.60618600
H	0.94906800	-2.86488800	-3.97685400
C	1.25587500	-0.73409800	-4.32981100
H	0.73984000	-0.71216200	-5.29178600
C	1.81479900	0.43035600	-3.80808500
H	1.74268700	1.36851400	-4.35949500
C	2.48915100	0.39748700	-2.57434500
C	3.17682000	1.52600000	-1.94231000
C	3.81082700	1.25561600	-0.68564400
C	4.48921200	2.33396400	-0.07582400
H	4.98625600	2.17493800	0.88447800
C	4.52747500	3.60726800	-0.65492300
H	5.05579700	4.41878700	-0.14571100
C	3.89192100	3.85201300	-1.88327300
H	3.91950900	4.84651300	-2.33421000
C	3.22434900	2.80907300	-2.52508400
H	2.73706700	2.99815800	-3.48513900
C	5.73871800	-1.61908900	-1.99187500
H	4.88170300	-1.65320900	-2.66651500
C	7.01225700	-1.97963500	-2.42049800

H	7.16508800	-2.30460900	-3.45035700
C	8.07076000	-1.90880900	-1.50560700
H	9.08468200	-2.18199700	-1.80494700
C	7.81548800	-1.48185000	-0.20493600
H	8.62812500	-1.41837000	0.51933100
C	6.50943900	-1.13024000	0.17889500
C	6.10226600	-0.66910200	1.50872500
C	7.01240500	-0.52327800	2.57574200
H	8.07172500	-0.75417900	2.43550200
C	6.57330500	-0.08037700	3.82345600
H	7.28231000	0.03278000	4.64679700
C	5.21232000	0.21350800	4.00808100
H	4.85835200	0.55738300	4.98466200
C	4.30522400	0.07183500	2.95222700
H	3.25263700	0.31064800	3.12473600
C	4.71145100	-0.36314700	1.67086300
Ir	3.56725400	-0.61649800	0.02832000
N	2.95748000	-2.56814600	0.69016400
N	2.55819000	-0.77400100	-1.86440500
N	5.48508200	-1.20927500	-0.73010600
H	-8.82498300	1.57227900	1.03683800
N	-3.66327200	-1.87978600	1.74046400
N	-4.54015000	-2.09524900	-2.36682500
C	-3.53146800	-1.78209800	2.89264700
C	-4.86416900	-2.16178800	-3.48283000
C	-5.26077400	-2.24025600	-4.86921600
H	-5.12718300	-3.26787200	-5.23873000
H	-6.31876400	-1.95604200	-4.97169100
H	-4.64446400	-1.55715700	-5.47271800
C	-3.34405300	-1.65235300	4.31841900
H	-2.28829100	-1.83196000	4.57180500
H	-3.62365200	-0.63800200	4.64028000
H	-3.97198200	-2.38429200	4.84799800

IrPPY/Co-PYN5

2 4

C	2.25752400	4.03724800	-0.78234100
C	1.25663900	4.49162500	0.12531300
C	1.07457300	3.93822000	1.44042200
H	1.76136300	3.16803500	1.79340600
C	0.07283700	4.36921400	2.26931700
H	-0.04388400	3.93140400	3.26402600
C	-0.83746700	5.40074400	1.86784500
C	-1.89624000	5.83242200	2.69296300
H	-2.03135600	5.36201600	3.67009500
C	-2.76620300	6.83907200	2.26570400
H	-3.58616200	7.15756900	2.91346300
C	-2.59525700	7.44293100	1.01618800
H	-3.27731100	8.23072400	0.68638500
C	-1.54526500	7.04974000	0.16175800
C	-1.34072400	7.65763600	-1.12291900
H	-2.02149200	8.45422100	-1.43407800
C	-0.31858300	7.25564700	-1.94030900
H	-0.16703400	7.72658900	-2.91499800
C	0.57674600	6.20422200	-1.54951600
C	1.62432200	5.76997900	-2.38595100
H	1.77052600	6.25629100	-3.35347800
C	2.44196100	4.71038400	-2.00605300
H	3.21255800	4.35421600	-2.69369400
C	0.39899000	5.57064900	-0.27604800
C	-0.65512800	6.00817000	0.58450200
C	-1.23767600	-0.16865800	-1.83545000
H	-1.20157100	-1.21050400	-2.15803500
C	-0.41786800	0.79838500	-2.40989500
H	0.28079300	0.51676700	-3.19871700
C	-0.51783200	2.11967400	-1.95260400
H	0.10609400	2.90694000	-2.38002600
C	-1.42340200	2.41837400	-0.93781600
H	-1.51375300	3.43945300	-0.56635000
C	-2.23355200	1.40488700	-0.39573100
C	-3.25011300	1.58940900	0.64169000
C	-4.01750600	0.42980900	0.98493000
C	-5.00342800	0.59885900	1.98289700
H	-5.61412700	-0.25918900	2.27632500
C	-5.21681900	1.83087200	2.61180700
C	-4.44834700	2.95375300	2.26139700
H	-4.61341200	3.91626500	2.75136900
C	-3.46975400	2.82922200	1.27546000
H	-2.87245300	3.70254500	1.00384000
C	-4.90765900	0.05900800	-2.53488700
H	-3.92612800	0.52372000	-2.63730800
C	-5.93316000	0.33091500	-3.43520000
H	-5.75880600	1.01992200	-4.26249700
C	-7.17314200	-0.29189100	-3.24447500
H	-8.00301500	-0.10080700	-3.92787000
C	-7.33841600	-1.15704600	-2.16584700
H	-8.29920900	-1.64416300	-1.99591900
C	-6.26843800	-1.40075600	-1.28689700
C	-6.30941500	-2.27771700	-0.11319600
C	-5.10920500	-2.34718400	0.66574200
C	-5.14557300	-3.17861900	1.80745300
H	-4.25480100	-3.26018400	2.43535500
C	-6.29058700	-3.90027500	2.16264700
H	-6.27770600	-4.53181100	3.05613900

C	-7.45449800	-3.82151900	1.38107300
H	-8.34738300	-4.38788400	1.65566700
C	-7.45916700	-3.01053700	0.24616100
H	-8.36494100	-2.95106000	-0.36298700
C	-3.14096000	-3.38648500	-2.28407500
H	-3.86320400	-2.75253400	-2.80115800
C	-2.60810200	-4.52428700	-2.88273000
H	-2.91408200	-4.79905500	-3.89289000
C	-1.68500100	-5.29079300	-2.15917100
H	-1.24471800	-6.18932900	-2.59628400
C	-1.33382800	-4.89323700	-0.87127200
H	-0.61662400	-5.47583200	-0.29181600
C	-1.90299100	-3.73565200	-0.31227500
C	-1.61926200	-3.20079100	1.02145300
C	-0.76414900	-3.85087800	1.93445400
H	-0.28717300	-4.79713200	1.66500000
C	-0.52315300	-3.29770900	3.19298700
H	0.13786800	-3.80549600	3.89913300
C	-1.13587300	-2.08070300	3.53619300
H	-0.94799300	-1.63626400	4.51823400
C	-1.98880600	-1.43485400	2.63461300
H	-2.46121500	-0.49504900	2.93272400
C	-2.27269300	-1.97074000	1.35777100
Ir	-3.55596200	-1.25552200	-0.02531500
N	-2.12714100	0.11813500	-0.85921500
N	-5.06064700	-0.78459600	-1.49176100
N	-2.79982000	-2.99487900	-1.03790500
Co	5.62837300	-1.40631600	0.08682200
N	4.51647900	0.44028500	-0.18276400
N	7.03264100	0.21475200	-0.63691200
N	3.45832400	-1.78847500	0.56971100
N	5.43743700	-3.54185800	0.87085000
H	5.46996500	-3.38733700	1.88641400
N	7.68737900	-2.34174500	-0.14619700
H	7.68249600	-2.61547100	-1.13690000
C	6.57869200	1.39634900	-0.89047600
C	5.12593300	1.55823000	-0.62583900
C	3.19691800	0.47687800	0.08649300
C	4.43036700	2.75980900	-0.81057600
H	4.95522400	3.65021100	-1.15770900
C	3.05220700	2.81664400	-0.53238200
C	2.43575600	1.63648600	-0.07938200
H	1.36415600	1.61359600	0.11091900
C	2.62105700	-0.80640400	0.55058500
C	7.35464600	2.56026400	-1.42325900
H	8.41257800	2.32403600	-1.57809300
H	6.92427200	2.88258200	-2.38546900
H	7.27753800	3.41728800	-0.73520100
C	8.42957700	-0.14672800	-0.83494100
H	8.56472300	-0.43722000	-1.89341800
H	9.12204500	0.68267400	-0.62464700
C	3.06127600	-3.12678800	0.99198100
H	3.00237100	-3.14637100	2.09615600
H	2.07229600	-3.41370200	0.60141200
C	1.17967600	-0.85349600	0.94086500
H	0.89734100	-1.80789600	1.39515000
H	0.95311200	-0.04346200	1.65119500
H	0.54718000	-0.68255000	0.05478400
C	8.73553800	-1.33550200	0.06257900
H	8.70648100	-1.03275200	1.12078700
H	9.74286600	-1.73236400	-0.15726400
C	4.12718200	-4.10018600	0.51547300
H	4.09256800	-4.20158100	-0.58032400
H	3.96337900	-5.09775000	0.96133300
C	7.83900200	-3.55539700	0.67088700
H	8.74006900	-4.13073200	0.39356800
H	7.94263500	-3.24451500	1.72342000
C	6.58146600	-4.38400500	0.48935100
H	6.62532100	-5.31463300	1.08211100
H	6.46003300	-4.66128400	-0.57079600
H	-5.98850900	1.91975100	3.38245300
N	5.24970800	-1.99197000	-1.89278200
N	6.02859500	-0.84218100	2.07268800
C	6.15453900	-0.44028400	3.15778000
C	5.01645200	-2.24113000	-3.00560700
C	6.31215300	0.06149900	4.50267200
H	6.52294000	1.14103000	4.47501800
H	5.38886300	-0.11098500	5.07572800
H	7.14649700	-0.45546800	4.99963700
C	4.72004000	-2.55449600	-4.38387900
H	3.87472200	-3.25727200	-4.43269900
H	4.45606500	-1.63506800	-4.92769500
H	5.59844000	-3.01461800	-4.86035700

Co-PYN5

2 4

Co	-3.36785800	-0.14269100	0.00466800
N	-1.23492500	0.28489200	0.01770400
N	-2.96838400	1.65423800	-1.30273200
N	-2.32575300	-1.62199000	1.36173200
N	-4.96604700	-1.48367900	0.91961600
H	-5.16036800	-0.99624900	1.80335000
N	-5.33601200	0.45376400	-0.98603600
H	-5.26505000	-0.02384600	-1.89327200

C	-1.75583300	2.05621400	-1.48838600
C	-0.73840100	1.30170000	-0.71451500
C	3.69231200	-1.18750400	-0.42022100
H	2.65796500	-1.48909400	-0.59133800
C	-0.39480800	-0.45633900	0.76728900
C	0.62753000	1.60725000	-0.73051500
H	0.99899400	2.42737100	-1.34519800
C	1.52113800	0.84155200	0.03879300
C	3.97568200	0.18082600	-0.07848900
C	2.96336400	1.17124700	0.07852000
C	0.98101400	-0.20575500	0.80676100
H	1.62641100	-0.80239000	1.45026900
C	-1.05064400	-1.53853200	1.54379700
C	5.69553700	1.93592100	0.33008500
C	-1.31079500	3.16885000	-2.38568000
H	-2.14096200	3.61525100	-2.94280900
H	-0.56840000	2.79061400	-3.10677800
H	-0.81213600	3.95616100	-1.79721400
C	5.35143100	0.57085600	0.05908700
C	6.39045600	-0.40093200	-0.07589300
C	7.76267800	-0.01624900	0.08400800
C	4.69065000	-2.11501600	-0.55742500
H	4.44852300	-3.14777200	-0.82164100
C	6.06881500	-1.76384200	-0.37497300
C	4.66440000	2.88995600	0.43478900
H	4.92135900	3.93299100	0.63487800
C	3.33160700	2.51144200	0.30599200
H	2.54581800	3.26167300	0.42141400
C	-4.10204200	2.28077300	-1.97249200
H	-4.15746100	1.88975500	-3.00532600
H	-4.00490700	3.37568100	-2.03374100
C	-3.14901000	-2.61457500	2.03992000
H	-3.41019400	-2.21977800	3.03938300
H	-2.63277300	-3.57600900	2.18433900
C	-0.21296000	-2.39304500	2.44400500
H	-0.79442500	-3.17477900	2.94315300
H	0.25906900	-1.76378300	3.21645300
H	0.60275500	-2.86436800	1.87284300
C	7.07730900	2.29368900	0.48319500
H	7.31873600	3.33875400	0.69374400
C	8.06933600	1.35722500	0.36932900
H	9.11844100	1.63981000	0.48957300
C	-5.36432200	1.90332200	-1.21430800
H	-5.38396900	2.39926500	-0.23160500
H	-6.25926500	2.21931500	-1.77997500
C	-4.41563900	-2.80988700	1.22231300
H	-4.18219900	-3.30532900	0.26714400
H	-5.13405900	-3.44253600	1.77397900
C	7.10885600	-2.70746000	-0.49930100
H	6.85664400	-3.74573100	-0.72962300
C	8.76880400	-0.99505100	-0.04527100
H	9.81308400	-0.69855400	0.08200900
C	8.44226800	-2.32362500	-0.33283200
H	9.23655500	-3.06723000	-0.43084400
C	-6.50901400	-0.07313200	-0.27216000
H	-7.42631600	-0.01314500	-0.88386300
H	-6.66018900	0.54162900	0.63025900
C	-6.19983200	-1.50534100	0.11960600
H	-7.04186200	-1.95995200	0.67095100
H	-6.01101600	-2.11524100	-0.77900400
N	-3.09715500	-1.45410600	-1.61381000
N	-3.69467200	1.15402900	1.62199400
C	-2.87114400	-2.14742100	-2.52099600
C	-3.81053300	1.87290400	2.53006100
C	-2.59153600	-3.01315900	-3.64240700
H	-3.25903800	-3.88743200	-3.61435900
H	-2.75155000	-2.46832000	-4.58476900
H	-1.54700800	-3.35570700	-3.59721800
C	-3.95931100	2.76834800	3.65325600
H	-4.90351000	3.32595700	3.56327700
H	-3.96795600	2.19346100	4.59120200
H	-3.12110300	3.48088400	3.67540100

Co-N5

2 4

Co	-0.56307700	-0.00223700	0.00146000
N	1.62344800	0.00695200	0.01555400
N	0.16723100	2.13147200	-0.08975500
N	0.18404200	-2.12725900	0.14057500
N	-2.38250000	-1.36125800	0.15915000
H	-2.47278600	-1.46573000	1.17767700
N	-2.38504300	1.35058500	-0.23210400
H	-2.41496600	1.48422200	-1.25071900
C	1.43272700	2.37622500	-0.16041300
C	2.29508400	1.17117500	-0.08070000
C	2.30428600	-1.15232300	0.10667300
C	3.69752600	1.21466000	-0.09618800
H	4.22089600	2.16726400	-0.17656200
C	4.40739200	0.01633000	-0.00369200
C	3.70698600	-1.18651900	0.10171000
H	4.23757500	-2.13560200	0.17603000
C	1.45137200	-2.36301600	0.20544900
C	2.07593000	3.71868600	-0.31674600
H	1.34261800	4.52555200	-0.41657100

H	2.72143100	3.72003500	-1.20996700
H	2.72608600	3.93234800	0.54727500
C	-0.82974900	3.19434000	-0.14668600
H	-0.97022200	3.48525400	-1.20432500
H	-0.52332900	4.09361600	0.40979600
C	-0.80953100	-3.19132900	0.21044400
H	-0.99335100	-3.42571700	1.27547700
H	-0.48128800	-4.11733100	-0.28587200
C	2.10647900	-3.69854900	0.37270300
H	1.38098100	-4.50706000	0.50923600
H	2.77607800	-3.67765000	1.24757100
H	2.73411600	-3.92670000	-0.50435800
C	-2.13333300	2.64553700	0.40980700
H	-2.04530900	2.47237000	1.49361400
H	-2.95541400	3.36357300	0.23864600
C	-2.08965300	-2.67491100	-0.42594800
H	-1.94986200	-2.53893700	-1.50973000
H	-2.91372300	-3.39390700	-0.26940200
C	-3.63214100	0.69410500	0.18906200
H	-4.52557800	1.24064000	-0.16065700
H	-3.65154100	0.67804000	1.29133500
C	-3.60460400	-0.72251800	-0.35212600
H	-4.51369300	-1.27948300	-0.06393600
H	-3.54692900	-0.70949600	-1.45303000
H	5.49871300	0.01988700	-0.01256800
N	-0.54813400	-0.14046000	-2.09232500
N	-0.61878300	0.13133100	2.09399900
C	-0.45486700	-0.17553300	-3.25201400
C	-0.54730200	0.16597000	3.25526600
C	-0.45760200	0.21295900	4.69576500
H	-1.34671400	-0.25714400	5.14168200
H	0.44106200	-0.32648200	5.03078800
H	-0.39601400	1.25869600	5.03204700
C	-0.34016400	-0.22173700	-4.69076700
H	-1.22232300	0.24736000	-5.15134500
H	0.56325400	0.31913500	-5.01024100
H	-0.27152800	-1.26719500	-5.02655200

IrPPPY

0 1

C	3.32083300	-0.48603100	-1.06185000
H	2.29320700	-0.44090600	-1.42318400
C	3.96052900	0.72880800	-0.62858900
C	3.29674100	1.99255000	-0.60493600
C	6.05839200	1.84547200	0.11631400
C	5.34353900	0.65657900	-0.24496900
C	6.01981100	-0.60325700	-0.21932800
C	7.39389300	-0.67743500	0.18697200
C	3.97047500	-1.69117200	-1.04153300
H	3.45650300	-2.59628400	-1.37645000
C	5.33054000	-1.80041200	-0.59963900
C	5.38346300	3.08125300	0.07359900
H	5.92507200	3.99206800	0.34202900
C	4.03759300	3.14537100	-0.27715300
H	3.52471400	4.11010300	-0.26574100
C	7.43509400	1.74331600	0.51062200
H	7.96327000	2.66097800	0.78344900
C	8.07582700	0.53393700	0.54858400
H	9.12343800	0.46820700	0.85426800
C	6.01152200	-3.03395700	-0.55327800
H	5.47880900	-3.94283700	-0.84496700
C	8.03345400	-1.93375700	0.21872700
H	9.07889900	-1.98590700	0.53370900
C	7.34684000	-3.09590300	-0.14510700
H	7.85986000	-4.06003100	-0.11339000
C	-4.38643300	1.24431900	-1.29558200
H	-4.88568700	0.42858700	-0.76974000
C	-5.09215200	2.12858000	-2.10630200
H	-6.17021000	2.01263800	-2.22459600
C	-4.38576700	3.14880900	-2.75704700
H	-4.90477300	3.85942600	-3.40356200
C	-3.00885600	3.24666300	-2.57145100
H	-2.44104700	4.03263900	-3.07073400
C	-2.34386900	2.32916100	-1.73906500
C	-0.90783900	2.31415900	-1.45585500
C	-0.45570200	1.28935600	-0.56517400
C	0.92224200	1.25012000	-0.28623300
H	1.29586200	0.50380700	0.41727100
C	1.84826900	2.12230900	-0.88953600
C	1.36765000	3.12888400	-1.75809800
H	2.06983600	3.81511100	-2.23722700
C	0.00413200	3.22487800	-2.02737800
H	-0.34602800	4.00550200	-2.70808800
C	-2.88986600	2.16736900	2.10167800
H	-3.46572700	2.48259500	1.22996000
C	-2.98713100	2.83442400	3.31884500
H	-3.65353900	3.69249200	3.41477300
C	-2.21179500	2.38005800	4.39375200
H	-2.25900000	2.87936100	5.36365500
C	-1.37464000	1.28202100	4.21111600
H	-0.76106400	0.91760800	5.03573100
C	-1.31653900	0.64001800	2.96149800
C	-0.48621200	-0.52109100	2.62997400
C	0.20376600	-2.12470000	0.95208200

H	0.15167300	-2.53024200	-0.06170600
C	1.08262400	-2.70807400	1.87120400
H	1.69864800	-3.55991200	1.56744200
C	1.18350100	-2.20530800	3.17890600
H	1.87091900	-2.65941800	3.89628200
C	0.39631800	-1.11654200	3.55448800
H	0.47685800	-0.72647900	4.57260500
C	-4.28262200	-1.35119700	1.53749900
H	-4.12466300	-0.53181300	2.24043900
C	-5.29181400	-2.28963300	1.73054000
H	-5.94455600	-2.21194300	2.60085500
C	-5.43606500	-3.31930900	0.79169700
H	-6.21518100	-4.07517600	0.90999900
C	-4.56963000	-3.37020200	-0.29732300
H	-4.66364000	-4.16601200	-1.03714900
C	-3.56792000	-2.39570900	-0.44737700
C	-2.59442200	-2.33069600	-1.54083600
C	-2.55440700	-3.28045500	-2.58237600
H	-3.27198700	-4.10509200	-2.60389700
C	-1.59774900	-3.18024200	-3.59289100
H	-1.56837300	-3.91900900	-4.39714500
C	-0.67665100	-2.12022300	-3.56353700
H	0.07805300	-2.03375800	-4.35110800
C	-0.71478900	-1.17415100	-2.53318400
H	0.01837300	-0.36363600	-2.53487900
C	-1.66410200	-1.24312900	-1.48898600
Ir	-1.86230500	0.00522800	0.08831500
N	-3.05267200	1.33622700	-1.11016800
N	-3.44323300	-1.39303500	0.48007700
C	-0.60144500	-1.01375400	1.28964500
N	-2.08449300	1.09856400	1.92181300

IrPPY

0 1

C	0.32549300	-2.20373100	-2.15625900
H	1.12371600	-1.53966200	-2.49224600
C	0.04290500	-3.39307500	-2.82104400
H	0.62647200	-3.67543800	-3.69818400
C	-0.99184200	-4.20186900	-2.33361500
H	-1.24079700	-5.14375100	-2.82684700
C	-1.70118200	-3.79159900	-1.20733900
H	-2.50742600	-4.40914500	-0.81042600
C	-1.37888800	-2.57798400	-0.57524100
C	-2.04113800	-2.02508900	0.60939400
C	-1.53036500	-0.77530500	1.08905900
C	-2.15895900	-0.23355200	2.23225200
H	-1.80197100	0.71827400	2.63433900
C	-3.22264200	-0.88207100	2.86966400
C	-3.70628400	-2.10753900	2.38234900
H	-4.53747600	-2.61304500	2.87937300
C	-3.11465200	-2.67391600	1.25298500
H	-3.49272400	-3.62704700	0.87299600
C	-2.06528500	0.80482200	-2.17569300
H	-1.88372700	-0.22088300	-2.50057600
C	-2.95053300	1.63712500	-2.85398000
H	-3.48027400	1.26449000	-3.73153900
C	-3.13742400	2.94234000	-2.38029900
H	-3.82454600	3.62436800	-2.88530600
C	-2.43599800	3.36225600	-1.25275300
H	-2.56883000	4.37456100	-0.86916100
C	-1.55179500	2.48183300	-0.60504500
C	-0.75042100	2.79179500	0.58191700
C	0.86877800	2.02816800	2.21074600
H	1.52363900	1.25350000	2.61833200
C	0.82211100	3.27645000	2.84144400
H	1.44023500	3.46117100	3.72522700
C	-0.01408800	4.29295900	2.35079900
H	-0.05291800	5.26647200	2.84493700
C	-0.79548400	4.04720400	1.22170600
H	-1.44635700	4.83800900	0.83924600
C	1.74067300	1.38772900	-2.17157400
H	0.76571700	1.76120200	-2.48886100
C	2.90552300	1.72373900	-2.85468400
H	2.85301400	2.37482200	-3.72815100
C	4.12409200	1.21171200	-2.39015100
H	5.05931800	1.45328400	-2.89945200
C	4.13041900	0.39006400	-1.26564600
H	5.06971300	-0.01429100	-0.88697700
C	2.92492900	0.08010100	-0.61229300
C	2.78698600	-0.76438500	0.57721200
C	3.89015400	-1.37364600	1.20924400
H	4.90012900	-1.23337800	0.81460500
C	3.70582500	-2.16136500	2.34568800
H	4.56279600	-2.63184400	2.83323200
C	2.40834600	-2.34284800	2.85252800
H	2.25473800	-2.95940700	3.74333800
C	1.31040100	-1.74050300	2.22815200
H	0.31239600	-1.89610600	2.64619600
C	1.45499900	-0.93303300	1.07783800
Ir	0.00443000	0.00630000	0.03497300
N	-0.36300800	-1.79976400	-1.06721300
N	1.74131800	0.58538400	-1.08558300
C	0.08774100	1.73697700	1.06979000
N	-1.37970200	1.20900400	-1.08505300

H	-3.68147400	-0.43050900	3.75446200

IrPPPY/IrPPPY

0 1

Ir	-8.45130300	0.00505300	-0.05154600
N	-9.14878100	-1.85100300	-0.88834600
N	-10.31837200	1.04509300	-0.22051500
C	-9.32077500	-0.49840900	1.69690900
N	-7.56968300	0.77167800	-1.85819100
C	-6.14777500	2.47112100	-2.78937600
H	-5.56112500	3.38077100	-2.65611600
C	-11.92028100	2.46388100	-1.31481400
H	-12.17854800	3.07040400	-2.18375000
C	-6.83304000	1.91624600	-1.69404800
C	0.42684300	2.39637600	1.26652200
C	-9.51161800	-1.59910600	3.88668700
H	-9.07095100	-2.24357700	4.65328900
C	-6.80568600	-1.14509100	0.13696000
C	-7.57067700	1.68470400	0.63909100
C	-12.41059400	1.53841400	0.85571200
H	-13.07701200	1.41827900	1.71063400
C	-0.58076300	-0.16131600	2.03194200
C	-10.39173100	-2.07605400	-1.36367700
H	-11.07655900	-1.22663600	-1.33148900
C	-6.88575000	3.34606000	2.31705200
H	-6.90720300	3.69360900	3.35432000
C	-1.32626200	0.64532700	1.11194600
C	-10.78767800	-3.31120100	-1.86877700
H	-11.80359900	-3.44291500	-2.24310700
C	-8.57526100	-4.13377500	-1.38363400
H	-7.83930200	-4.93872700	-1.37902300
C	-5.54521400	-0.74222700	0.61352200
H	-5.43191300	0.26706800	1.01430800
C	-6.16257800	4.06943900	1.35447100
H	-5.61948700	4.97537800	1.63314900
C	-12.79495900	2.32284000	-0.22931400
H	-13.76619800	2.82178900	-0.22953100
C	-11.15931000	0.89760400	0.85270500
C	-8.79713700	-1.32585500	2.71478600
H	-7.80285600	-1.76159200	2.58604900
C	-0.80958700	1.91882900	0.71869900
C	-10.62777000	0.04460100	1.91951500
C	-10.78936900	-1.05076400	4.08679600
H	-11.34673400	-1.26315700	5.00206300
C	-5.80435700	-3.35190700	-0.33440400
H	-5.89439500	-4.37687100	-0.70413100
C	-7.57567800	2.18146200	1.96149800
H	-8.12360100	1.63424800	2.73310200
C	-6.91572300	-2.48449100	-0.35432100
C	-2.57984600	0.17870600	0.58703400
C	-3.23321600	0.99053000	-0.40624400
H	-4.14567200	0.62000000	-0.87376500
C	-2.31505000	-1.86441000	1.89976500
H	-2.70489200	-2.83430400	2.21754800
C	-8.22454100	-2.86557600	-0.88805500
C	-4.41421100	-1.58039700	0.58302100
C	-6.21711500	1.85849700	-4.03854000
H	-5.68410500	2.28643900	-4.89005600
C	-6.83638200	2.44514600	-0.32793300
C	-1.09079100	-1.42386200	2.39341800
H	-0.51830800	-2.05082300	3.08162500
C	-9.85825300	-4.35955600	-1.87660000
H	-10.13324800	-5.34348500	-2.26205100
C	-1.52017500	2.72591800	-0.22782200
C	1.14474900	1.56246100	2.18815900
H	2.09549300	1.92735700	2.58575300
C	-7.63190800	0.17964600	-3.06940600
H	-8.22975800	-0.73180100	-3.12405800
C	-2.73071600	2.20401900	-0.79195100
H	-3.24677500	2.79310600	-1.55492200
C	-3.09146000	-1.07957000	1.02389100
C	-11.34320500	-0.23206500	3.10217400
H	-12.33855400	0.19287200	3.25792500
C	0.66235800	0.33328100	2.55075100
H	1.22160100	-0.30120900	3.24319700
C	-4.56622300	-2.90940300	0.12576100
H	-3.70277900	-3.57736300	0.10224700
C	0.19893100	4.44592900	-0.04136200
H	0.58954400	5.42360400	-0.33367000
C	-6.14319600	3.61774900	0.03477300
H	-5.57992300	4.18062800	-0.71390900
C	-0.99769900	3.98214400	-0.59566100
H	-1.54227100	4.58875200	-1.32404600
C	-6.97507700	0.68948500	-4.18559100
H	-7.05552500	0.17538300	-5.14407000
C	-10.69280700	1.81020500	-1.26789700
H	-9.97238000	1.88661100	-2.08436500
C	0.90399500	3.66557200	0.87904000
H	1.84183700	4.02880200	1.30658900
Ir	8.45160600	-0.00504200	0.05147400
N	9.14904500	1.85077600	0.88847300
N	10.31849800	-1.04534100	0.22017200
C	9.32083300	0.49834700	-1.69711100
N	7.57014000	-0.77168200	1.85816300

C	6.14803700	-2.47099600	2.78933400
H	5.56125900	-3.38055500	2.65599600
C	11.92000400	-2.46509300	1.31381300
H	12.17828700	-3.07172700	2.18266600
C	6.83333900	-1.91615500	1.69401100
C	-0.42756400	-2.39522200	-1.26667600
C	9.51162300	1.59912200	-3.88685400
H	9.07101700	2.24381400	-4.65330900
C	6.80585300	1.14504800	-0.13674700
C	7.57093300	-1.68472300	-0.63916000
C	12.40991900	-1.54007200	-0.85699200
H	13.07600400	-1.42045200	-1.71223300
C	0.58075300	0.16224200	-2.03185800
C	10.39205700	2.07577800	1.36373300
H	11.07697300	1.22645600	1.33106800
C	6.88605600	-3.34619800	-2.31700800
H	6.90759400	-3.69387500	-3.35423100
C	1.32604700	-0.64472000	-1.11197800
C	10.78792000	3.31074400	1.86931300
H	11.80386900	3.44240500	2.24358600
C	8.57537400	4.13330700	1.38479100
H	7.83933000	4.93818200	1.38056800
C	5.54539400	0.74230800	-0.61339800
H	5.43210200	-0.26681700	-1.01465200
C	6.16279200	-4.06945900	-1.35441100
H	5.61966500	-4.97538800	-1.63305600
C	12.79425100	-2.32473000	0.22787700
H	13.76511400	-2.82441100	0.22763000
C	11.15907000	-0.89841500	-0.85342400
C	8.79729900	1.32606200	-2.71480300
H	7.80322100	1.76217700	-2.58580800
C	0.80905100	-1.91813100	-0.71889300
C	10.62759600	-0.04514800	-1.92004700
C	10.78911800	1.05032700	-4.08727900
H	11.34637100	1.26259500	-5.00264300
C	5.80438000	3.35153400	0.33589500
H	5.89431000	4.37629100	0.70622600
C	7.57597600	-2.18157000	-1.96152400
H	8.12391700	-1.63442300	-2.73315700
C	6.91584800	2.48426200	0.35509800
C	2.57971600	-0.17846800	-0.58695100
C	3.23292500	-0.99061900	0.40616500
H	4.14550400	-0.62039300	0.87370200
C	2.31534200	1.86499300	-1.89921900
H	2.70537200	2.83489900	-2.21672900
C	8.22471800	2.86527200	0.88875600
C	4.41431800	1.58038600	-0.58234800
C	6.21749700	-1.85846500	4.03853300
H	5.68447000	-2.28637800	4.89005000
C	6.83658200	-2.44506600	0.32789400
C	1.09104700	1.42475900	-2.39306400
H	0.51870700	2.05197800	-3.08115500
C	9.85839600	4.35901200	1.87770100
H	10.13335100	5.34280200	2.26353700
C	1.51942200	-2.72553500	0.22752500
C	-1.14523600	-1.56103000	-2.18824300
H	-2.09610100	-1.92561900	-2.58582900
C	7.63247800	-0.17974800	3.06942700
H	8.23045200	0.73161400	3.12413200
C	2.73013000	-2.20405100	0.79168400
H	3.24608100	-2.79339900	1.55453100
C	3.09155800	1.07980800	-1.02347200
C	11.34287600	0.23135100	-3.10282900
H	12.33805600	-0.19388800	-3.25883600
C	-0.66248500	-0.33197400	-2.55075500
H	-1.22154100	0.30274800	-3.24313900
C	4.56621500	2.90910300	-0.12429000
H	3.70259200	3.57677200	-0.09980800
C	-0.20034800	-4.44490800	0.04110600
H	-0.59133400	-5.42241800	0.33346600
C	6.14334200	-3.61765600	-0.03475200
H	5.57994500	-4.18041500	0.71393200
C	0.99654200	-3.98162000	0.59527300
H	1.54096500	-4.58848200	1.32355700
C	6.97563300	-0.68956800	4.18560700
H	7.05620500	-0.17551700	5.14410400
C	10.69294700	-1.81060500	1.26744500
H	9.97287500	-1.88643500	2.08427900
C	-0.90520500	-3.66422200	-0.87917000
H	-1.84346600	-4.02678900	-1.30638300

CoPYN5/CoPYN5

4 7

Co	-9.02322400	-0.02050300	0.06448700
N	-6.90973900	-0.13025400	-0.43566400
N	-8.19590000	2.06804700	-0.10040100
N	-9.53536800	-0.03244800	-1.97192400
N	-8.41252100	-2.19181900	-0.09917700
N	-8.53635900	-0.02012600	2.11353900
N	-10.82955900	-1.28753300	0.61541700
H	-10.66323400	-1.45540700	1.61578000
N	-10.75145700	1.44263800	0.35522500
H	-11.03288700	1.63148200	-0.61509600
C	-6.96826200	2.24944000	-0.45670100

C	-6.19028900	0.99626200	-0.61403300
C	-2.18739000	-2.08845300	0.40742700
H	-3.18513200	-1.99451700	0.83702800
C	-6.29653900	-1.32408400	-0.55649500
C	-4.81987900	0.96389300	-0.88973100
H	-4.26232000	1.89294100	-0.99470900
C	-4.15362600	-0.27100900	-0.99695400
C	-1.81504800	-1.24133000	-0.69373000
C	-2.71029400	-0.31953700	-1.31305000
C	-4.93263400	-1.43403300	-0.84905200
H	-4.48498600	-2.41782900	-0.98262800
C	-7.18254100	-2.49590300	-0.34495500
C	0.00227100	-0.41449500	-2.18368000
C	-6.31265800	3.57623900	-0.69169500
H	-7.04857200	4.35439200	-0.92666700
H	-5.58983400	3.51828500	-1.51710400
H	-5.75821800	3.88696100	0.21039100
C	-0.45592700	-1.30076700	-1.15477100
C	0.45112200	-2.25424600	-0.59873800
C	-9.75852700	-0.01011300	-3.11405600
C	1.79178900	-2.34865900	-1.09620200
C	-1.31126200	-2.99004200	0.95123000
H	-1.61951200	-3.61559100	1.79292100
C	-8.12215800	-0.04687500	3.20102900
C	0.02519900	-3.12726600	0.45292600
C	-0.89637600	0.52884500	-2.71758700
H	-0.54857400	1.21818600	-3.49045500
C	-2.21938200	0.56740100	-2.29233900
H	-2.90837800	1.27707300	-2.75524500
C	-9.09875500	3.18992300	0.12560500
H	-9.46633700	3.54650300	-0.85489200
H	-8.60252700	4.03656600	0.62505500
C	-9.43046200	-3.20395000	0.15220700
H	-9.35213200	-3.52000400	1.20903800
H	-9.30511100	-4.09956200	-0.47541400
C	-7.60517200	-0.07505500	4.54921300
H	-7.44043900	-1.11604800	4.86490100
H	-8.32231100	0.40008500	5.23507300
H	-6.65033000	0.47014700	4.59249200
C	-6.59111300	-3.87001000	-0.40708500
H	-7.31993700	-4.65504700	-0.18031400
H	-5.75789300	-3.94819900	0.30999000
H	-6.16824700	-4.05586000	-1.40773800
C	1.35950900	-0.50837800	-2.63959700
H	1.69120200	0.18509800	-3.41629200
C	2.21756500	-1.44271500	-2.12474200
H	3.24761300	-1.51278100	-2.48440400
C	-10.26616600	2.68843200	0.96050700
H	-9.93276900	2.45649100	1.98380500
H	-11.05542300	3.45934200	1.01936300
C	-10.79157900	-2.57289200	-0.09320200
H	-10.93291800	-2.37117700	-1.16635200
H	-11.59500700	-3.25447700	0.23920700
C	0.93219900	-4.06998800	0.97890700
H	0.60373700	-4.72519600	1.78964200
C	2.65416700	-3.32648400	-0.56119000
H	3.66674100	-3.41349800	-0.96231400
C	2.22908700	-4.17154900	0.46853800
H	2.91696900	-4.91583300	0.87670900
C	-11.88785200	0.82993700	1.05982600
H	-12.80328900	1.44193600	0.97775300
H	-11.62379600	0.75332700	2.12744000
C	-12.09565900	-0.55231400	0.47203900
H	-12.93442600	-1.07516800	0.96448000
H	-12.32447800	-0.47781200	-0.60369100
C	-10.03846300	0.01285100	-4.53056400
H	-9.76331000	-0.95242000	-4.98128300
H	-9.45886900	0.81387900	-5.01316800
H	-11.11094800	0.19469500	-4.69596900
Co	9.02096300	0.11483500	-0.12213700
N	6.92312700	0.14282900	0.45088900
N	8.24051000	-1.99719900	-0.07628500
N	9.61907400	-0.03871500	1.88566400
N	8.38510700	2.25333200	0.25362000
N	8.45065500	0.26299100	-2.14106300
N	10.77967500	1.46746200	-0.63839500
H	10.55864400	1.73414900	-1.60609800
N	10.75754900	-1.27547800	-0.60675300
H	11.09508100	-1.51822200	0.33340600
C	7.02918600	-2.23033000	0.30465200
C	6.22741000	-1.00754400	0.55731000
C	2.16863600	1.98441800	-0.33547600
H	3.16689600	1.91227900	-0.76862500
C	6.28884500	1.31369500	0.65740600
C	4.85833900	-1.02137100	0.84565500
H	4.31736300	-1.96524400	0.89811400
C	4.16948600	0.19137800	1.02833800
C	1.81512900	1.11910700	0.75763600
C	2.72790200	0.20242000	1.35611400
C	4.92241700	1.37744200	0.94974600
H	4.45193600	2.34143100	1.13910500
C	7.15486100	2.51356600	0.54450600
C	0.02744500	0.25556400	2.26041600
C	6.41392700	-3.58557700	0.47873700

H	7.17540200	-4.35134900	0.66935200
H	5.69720600	-3.59099500	1.31141800
H	5.86241900	-3.86868000	-0.43429700
C	0.46086400	1.15566200	1.23294400
C	-0.46398500	2.10204800	0.69445400
C	9.87806900	-0.16078800	3.01387200
C	-1.79993100	2.17569200	1.20792000
C	1.27465800	2.87737800	-0.86415800
H	1.56724900	3.51775700	-1.70028500
C	8.00489000	0.35847400	-3.21207200
C	-0.05987300	2.98937700	-0.35381100
C	0.94371100	-0.68326600	2.77281800
H	0.61370600	-1.38438700	3.54286500
C	2.26191300	-0.70317300	2.32949900
H	2.96759200	-1.40911200	2.77299900
C	9.16168900	-3.07768200	-0.40538200
H	9.58705700	-3.47526100	0.53522700
H	8.66548400	-3.91058100	-0.92738400
C	9.38220700	3.30280800	0.08201100
H	9.25981400	3.73676400	-0.92769600
H	9.27015500	4.11881800	0.81279800
C	7.44948800	0.47253900	-4.54023500
H	7.36737700	1.53315400	-4.82108200
H	8.09952100	-0.04226800	-5.26342000
H	6.44903800	0.01509000	-4.56541400
C	6.54610200	3.86588000	0.75091700
H	7.27240900	4.67753700	0.63964200
H	5.73339400	4.02180100	0.02252900
H	6.09210900	3.92790700	1.75311200
C	-1.32470000	0.33151000	2.73497300
H	-1.63818300	-0.37063500	3.51145700
C	-2.20045600	1.25857400	2.23688900
H	-3.22643900	1.31395500	2.61049500
C	10.27185100	-2.49401500	-1.26528700
H	9.87945800	-2.20977200	-2.25416200
H	11.07562900	-3.23766300	-1.41178700
C	10.75879600	2.66935600	0.20363100
H	10.94575600	2.35540200	1.24204800
H	11.54066000	3.39430100	-0.08552400
C	-0.98460800	3.92442500	-0.86223000
H	-0.67320200	4.59110900	-1.67034500
C	-2.68069500	3.14614300	0.68976300
H	-3.69145900	3.21389000	1.09969900
C	-2.27732600	4.00469900	-0.33757600
H	-2.97921500	4.74281500	-0.73301600
C	11.83885100	-0.58476800	-1.32573400
H	12.76632500	-1.18366800	-1.35132400
H	11.50747500	-0.42398200	-2.36480800
C	12.06256300	0.74673800	-0.63513800
H	12.86263200	1.32538100	-1.12928600
H	12.35766600	0.58431500	0.41456300
C	10.20215400	-0.30648000	4.41354200
H	9.94867700	0.61824900	4.95332200
H	9.63165200	-1.14286400	4.84425800
H	11.27780700	-0.50732100	4.52883400
